# A tunable azine covalent organic framework platform for visible light-induced hydrogen generation

**DOI:** 10.1038/ncomms9508

**Published:** 2015-09-30

**Authors:** Vijay S. Vyas, Frederik Haase, Linus Stegbauer, Gökcen Savasci, Filip Podjaski, Christian Ochsenfeld, Bettina V. Lotsch

**Affiliations:** 1 Max Planck Institute for Solid State Research, Heisenbergstr. 1, 70569 Stuttgart, Germany; 2 Department of Chemistry, University of Munich (LMU), Butenandtstr. 5-13, 81377 Munich, Germany; 3 Department of Chemistry, Center for Integrated Protein Science (CIPSM), University of Munich (LMU), Butenandtstrasse 5–13, 81377 Munich, Germany; 4 Nanosystems Initiative Munich (NIM) and Center for Nanoscience, Schellingstr. 4, 80799 Munich, Germany

## Abstract

Hydrogen evolution from photocatalytic reduction of water holds promise as a sustainable source of carbon-free energy. Covalent organic frameworks (COFs) present an interesting new class of photoactive materials, which combine three key features relevant to the photocatalytic process, namely crystallinity, porosity and tunability. Here we synthesize a series of water- and photostable 2D azine-linked COFs from hydrazine and triphenylarene aldehydes with varying number of nitrogen atoms. The electronic and steric variations in the precursors are transferred to the resulting frameworks, thus leading to a progressively enhanced light-induced hydrogen evolution with increasing nitrogen content in the frameworks. Our results demonstrate that by the rational design of COFs on a molecular level, it is possible to precisely adjust their structural and optoelectronic properties, thus resulting in enhanced photocatalytic activities. This is expected to spur further interest in these photofunctional frameworks where rational supramolecular engineering may lead to new material applications.

## Abstract (web summary)

Production of hydrogen via the photocatalytic reduction of water is an attractive source of energy, but the catalysts are often expensive and possess little room for modification. Here, the authors show that covalent organic frameworks can be tuned for optimal photocatalytic performance.

## Graphical abstract

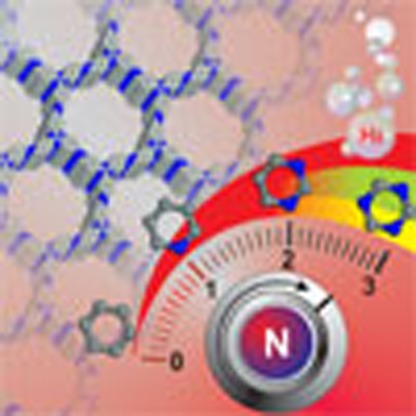

The past decade has witnessed the synthesis of a diverse collection of covalent organic frameworks (COFs) resulting from reversible condensation reactions^1^,^2^,^3^. By virtue of their light weight, high porosity and presence of organic chromophores, these COFs find applications in areas such as gas storage^4^,^5^,^6^,^7^, catalysis^8^ and sensing^9^. The structural regularity in COFs leads to long-range order, which has also encouraged the exploration of these materials as supramolecularly engineered organic semiconductors with a diverse set of optoelectronic properties^10^,^11^,^12^. As the optical and electronic properties of the resulting framework materials can readily be tuned by tailoring the organic precursors^13^,^14^,^15^,^16^,^17^, photofunctional COFs present an interesting area of research with a wide scope of potential applications.

Photocatalytic water splitting presents a promising method of producing clean energy from water by generating hydrogen using sunlight. Although continuing efforts are on to develop new materials as catalysts, the majority of them contain transition metals^18^ or rely on extended solids that offer little room for active-site engineering. New catalysts are usually investigated for their potential for photocatalytic water splitting by looking at hydrogen or oxygen evolution with a sacrificial electron donor or acceptor. This is widely regarded as the first step towards full water splitting^19^,^20^. Recent success in the synthesis of COFs that are not only air stable but also possess very good stability in acids and bases^9^,^21^,^22^ allows the exploration of these materials for applications such as photocatalytic water splitting where water and photostability are key requisites.

Here, we show that as a direct consequence of molecular engineering, a triphenylarene platform can readily be tuned for photocatalytic water reduction. Using a series of triphenylarylaldehydes with the central aryl ring containing 0–3 nitrogen atoms as building blocks, two-dimensional (2D) azine-linked COFs were synthesized, which reflect the structural variations of the triphenylarene platform. Investigation of these COFs as a new generation of polymeric photocatalysts show progressively enhanced hydrogen evolution with increasing nitrogen content in the frameworks. This work demonstrates the potential of organic materials in solar energy conversion where a vast array of organic building blocks and bond-forming reactions provide an extensive toolbox for the systematic fine-tuning of their structural and physical properties^23^, thus making way for the application of COFs in photocatalytic water splitting^24^.

## Results

### Synthesis and characterization of COFs

**Figure 1:**
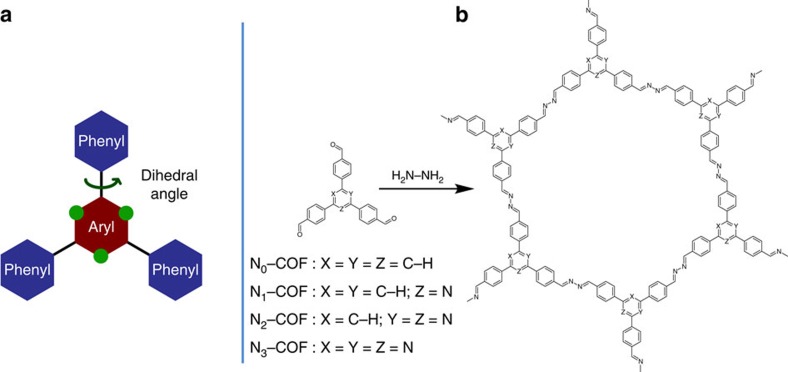
Design and synthesis of the N_*x*_–COFs. (**a**) A tunable triphenylarene platform for photocatalytic hydrogen evolution. Replacement of ‘C–H' by ‘nitrogen atoms' at the green dots changes the angle between central aryl and peripheral phenyl rings, which leads to varied planarity in the platform. (**b**) Synthesis of N_*x*_–COFs from N_*x*_–aldehydes and hydrazine.

A progressive substitution of alternate carbons in the central aryl ring of the triphenylaryl platform (Fig. 1a, green dots) by nitrogen atoms leads to a change in the electronic and steric properties of the central ring, that is, *N*=0 (phenyl), *N*=1 (pyridyl), *N*=2 (pyrimidyl) and *N*=3 (triazine). As a consequence of the substitution of the C–H moiety with nitrogen atoms, a change in dihedral angle between the central aryl ring and the peripheral phenyl rings is expected, which in turn leads to varied degrees of planarity in the platform. This was corroborated by density functional theory (DFT) calculations at the PBE0–D3/Def2–SVP level as evident by the decreasing dihedral angles in the energy-minimized structures of precursor aldehydes N_*x*_–Alds (Table 1). In addition, this results in a progressive decrease in electron density in the central aryl ring of the COF platform (Fig. 1a) as the number of nitrogen atoms increase from 0 to 3.

Encouraged by the theoretical calculations, we decided to synthesize N_*x*_–COFs (*x*=0, 1, 2 and 3) by an azine formation reaction^9^,^25^ of the aldehydes with hydrazine and investigate the translation of chemical and structural variation in the precursors to the overall order and optoelectronic properties of the resulting COFs, with the consequent influence on photocatalytic hydrogen production.

**Figure 2:**
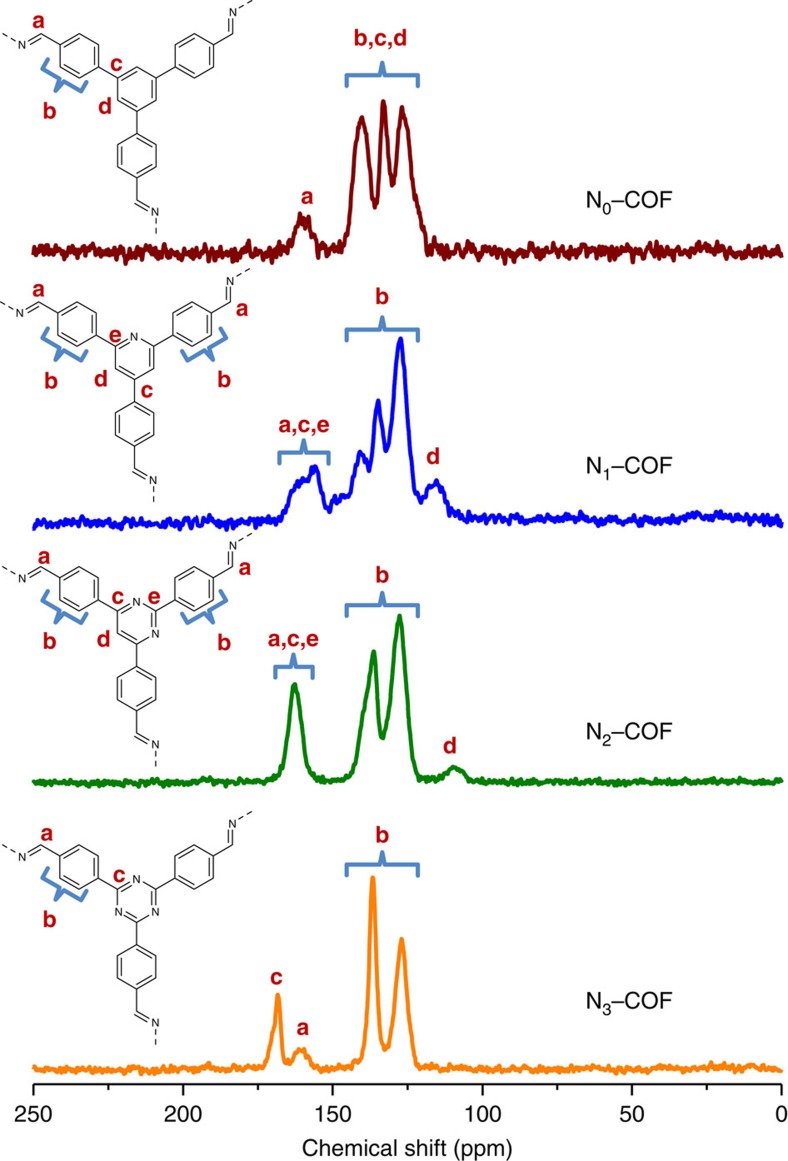
^13^C cross-polarization magic angle spinning solid-state NMR of the N_*x*_–COFs. The azine C=N peak (marked **a**) appears at ≈160 p.p.m. while the phenyl peaks (marked **b**) and characteristic central aryl peaks (marked **c**,**d**,**e**) show minimal changes with respect to their precursor aldehydes.

The precursor aldehydes (N_*x*_–Alds) were synthesized as described in the Supplementary Methods. N_*x*_–Alds as well as their precursors were characterized using ^1^H and ^13^C spectroscopy (Supplementary Figs 2–15). N_*x*_–COFs were synthesized in quantitative yields by a condensation reaction between the corresponding trialdehydes with hydrazine in the presence of 6 M acetic acid using 1:1 mesitylene/dioxane as solvent at 120 °C for 72 h (Fig. 1b; Supplementary Fig. 1). Fourier transform infrared (FTIR) spectra of the N_*x*_–COFs were compared with the corresponding aldehydes and hydrazine (Supplementary Figs 16–19), and show the disappearance of aldehydic C−H and C=O stretches and the appearance of the azine C=N stretch at 1,622 cm^−1^. These observations were further corroborated by the complementary Raman spectra (Supplementary Fig. 20), which showed characteristic Raman signals at 1,000–1,010 cm^−1^ for the ν(N–N) stretch, 1,540–1,560 cm^−1^ for the ν_sym_(C=N) stretch and at 1,600–1,625 cm^−1^ for ν_asym_ (C=N) stretch^26^. The composition and local structures of the N_*x*_–COFs were further confirmed by X-ray photoelectron spectroscopy (XPS; Supplementary Fig. 80) and ^13^C cross-polarization magic angle spinning (CP–MAS) solid-state NMR (ssNMR) spectroscopy. As seen in Fig. 2 and Supplementary Figs 50, 53, 56 and 59, the characteristic aldehyde carbonyl ^13^C resonance located at ≈190 p.p.m. in the precursor aldehydes disappears with the concomitant appearance of the azine C=N peak at ≈160 p.p.m., thereby attesting the conversion of the precursors into the respective COFs. The molecular structure of the building blocks remains intact during COF formation as evident by the largely unchanged chemical shifts of the peripheral phenyl rings as well as the central aryl ring that shows characteristic peaks in the NMR. Thus, for example, the C3 symmetric carbon in the central triazine ring in the N_3_–Ald at 171 p.p.m. shows minimal shift and appears at ∼168 p.p.m. in N_3_–COF. Similarly, the characteristic ^13^C NMR peak of the central pyridine/pyrimidine ring in N_1_–Ald and N_2_–Ald at 119 and 112 p.p.m., respectively, can easily be spotted in the corresponding COFs due to their upfield nature (Fig. 2). The assignment of these peaks was done using 2D heteronuclear multiple quantum coherence (HMQC) NMR of N_1_ and N_2_ aldehydes (Supplementary Figs 8 and 11).

### Structural and morphological characterization

**Figure 3:**
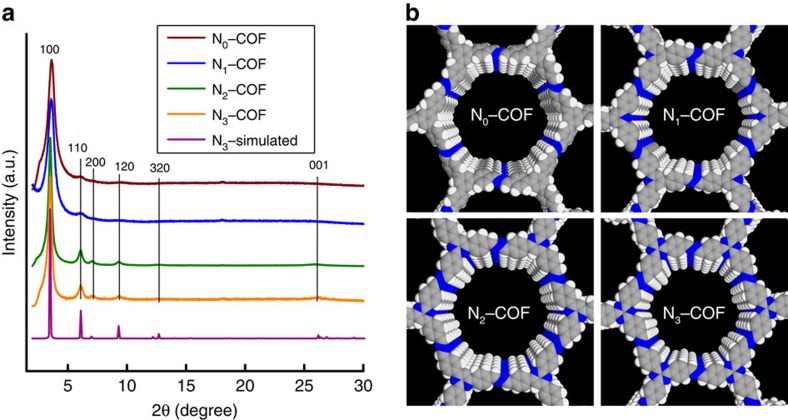
Structure and stacking analysis of the N_*x*_–COFs. (**a**) PXRD patterns of the N_*x*_–COFs compared with the simulated pattern calculated for the representative N_3_–COF. (**b**) View of extended stacks of N_*x*_–COFs in space filling model along the stacking direction (nitrogen, blue; carbon, grey; hydrogen, white). Note that an eclipsed stacking arrangement was assumed for simplicity.

To gain insight into the structural details and morphology of the COFs, powder X-ray diffraction (PXRD), gas sorption, scanning electron microscopy (SEM) and transmission electron microscopy (TEM) analyses were performed. PXRD of N_*x*_–COFs indicated the formation of crystalline networks with 2D honeycomb-type lattices as evident by the presence of an intense 100 reflection at 2θ=3.52° and reflections at 6.0, 7.1 and 9.5° corresponding to the 110, 200 and 120 reflections, respectively (Fig. 3). The observed reflections match well with the calculated patterns (Supplementary Figs 42–45) obtained from structural simulations performed for an AA eclipsed layer stacking (Supplementary Figs 34–41), using the Materials Studio v6.0.0 program (Accelrys). It should be noted that a slight lateral offset of the layers is expected for the stacked structures^27^, which however cannot be distinguished from the eclipsed topology by PXRD due to substantial peak broadening. Thus, for practical reasons, the eclipsed structure is used as a simplified working model^28^. Interestingly, as we traverse the series from N_0_ to N_3_–COF, the PXRD peaks become sharper with the appearance of prominent stacking peaks for N_2_ and N_3_–COF at 2θ=26° arising from the *d*-spacing between the 001 lattice planes. Thus, the variation in planarity in the precursor aldehydes as a function of the dihedral angles translates well into the crystallinity of the resulting COFs. The unit-cell parameters were obtained by Pawley refinements for all COFs and the results are included in Supplementary Table 1.

The permanent porosities evaluated by measuring the argon adsorption isotherm at 87 K (Supplementary Fig. 46) reveal that the Brunauer–Emmett–Teller (BET) surface area of symmetrical and unsymmetrical N_*x*_–COFs show a deviation from the trend expected from the increasing level of planarity from N_0_ to N_3_–Ald. Thus, while PXRD shows a similar yet lower degree of crystallinity for N_0_ and N_1_–COF in comparison to that observed for N_2_ and N_3_–COF, a further demarcation is seen in the BET area of the symmetrical and unsymmetrical COFs. Interestingly, the BET surface area of the symmetrical N_0_–COF was found to be 702 m^2^ g^−1^, which is significantly higher than that of the unsymmetrical N_1_–COF with a BET area of 326 m^2^ g^−1^. Likewise, in case of comparatively planar N_2_ and N_3_–COF, the symmetrical N_3_–COF showed a higher BET area of 1,537 m^2^ g^−1^ as compared with the unsymmetrical N_2_–COF with a BET area of 1,046 m^2^ g^−1^. Thus, the lowering of symmetry leads to a lower degree of order and hence lower BET surface area. Accordingly, a narrow pore-size distribution for the most crystalline N_3_–COF was calculated by the non-local DFT method with a peak maximum at ≈24 Å, which is in excellent agreement with the pore size established from the structural analysis and simulations. The maximum at 24 Å is accompanied by a minor peak in the pore size distribution (PSD) at ≈16 Å, which becomes more prominent in the other N_*x*_–COFs (*x*=0–2; Supplementary Fig. 47), likely resulting from an increasing degree of lateral layer offsets and stacking disorder in the less crystalline materials.

**Figure 4:**
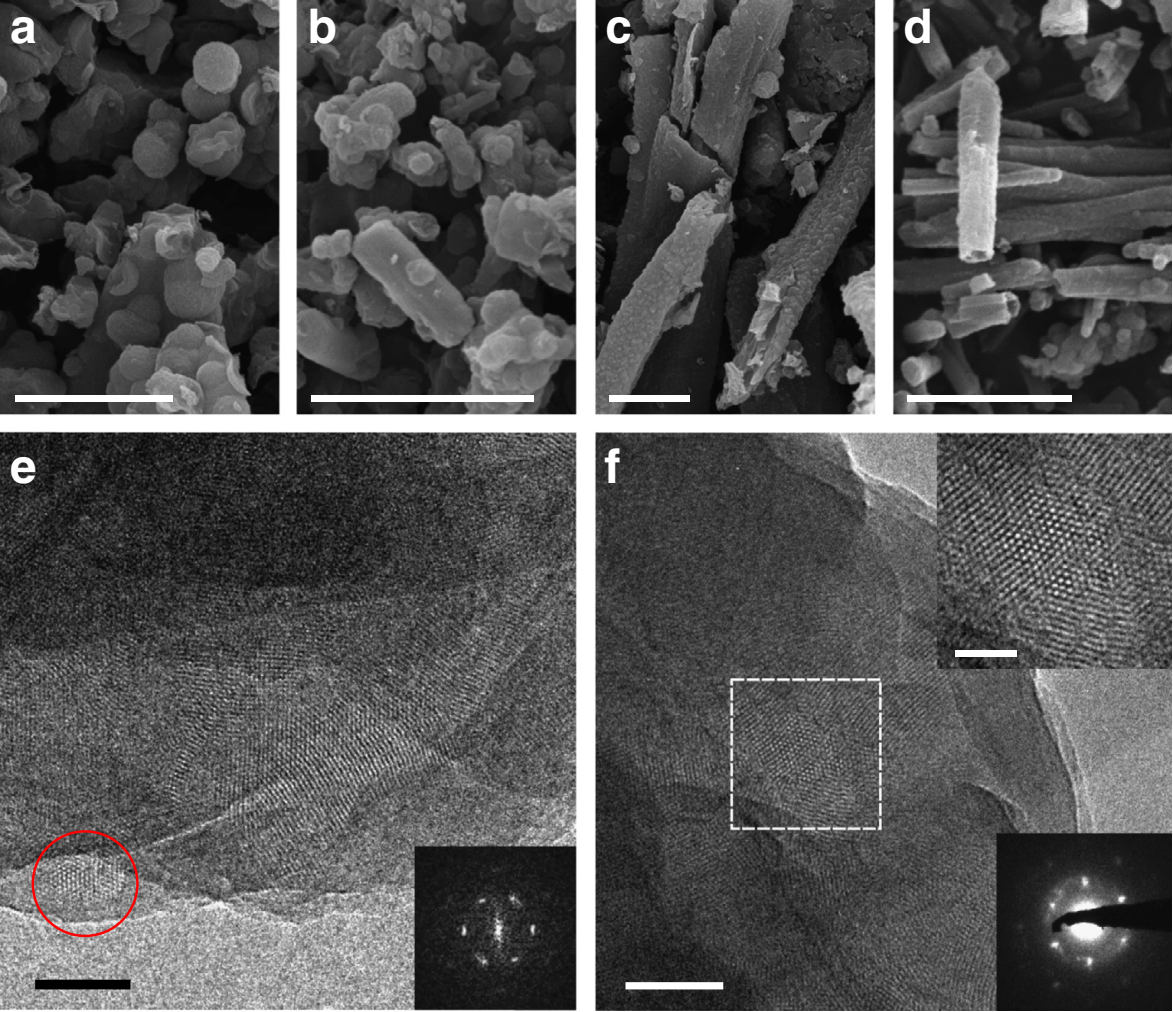
SEM and TEM images of N_*x*_–COFs. (**a**) SEM images of N_0_–COF, (**b**) N_1_–COF, (**c**) N_2_–COF and (**d**) N_3_–COF indicating morphological variation along the series. (**e**) TEM image of N_2_–COF showing hexagonal pores, with fast Fourier transform (FFT) of the marked area (red circle) in the inset. (**f**) TEM image of N_3_–COF with enlarged Fourier-filtered image (upper inset) of the marked area and representative selected area electron diffraction pattern (lower inset). Scale bars, 5 μm (**a**,**b**,**c**,**d**); 50 nm (**e** and **f**); 20 nm (**f**, upper inset).

SEM images show a change in morphology from purely ball-like agglomerates in N_0_–COF to elongated ones in N_1_–COF followed by transformation to rod-like morphology in N_2_ and N_3_–COF (Fig. 4a–d; Supplementary Fig. 21). The structure and morphology of N_*x*_–COFs was further investigated by TEM analysis where hexagonal pores are clearly visible in N_2_ and N_3_–COF (Fig. 4e,f; Supplementary Figs 24 and 25). Selected area electron diffraction along the [001] zone axis of the multilayers of N_3_–COF is consistent with a nanocrystalline, hexagonally ordered in-plane structure composed of crystalline domains with sizes ∼50–100 nm. TEM analysis of N_0_ and N_1_–COF, however, did not show any crystalline domains, possibly due to instability in the electron beam (Supplementary Figs 22 and 23).

The thermal stability of the COFs was investigated by thermogravimetric analysis (TGA), suggesting that the COFs are stable up to ≈350 °C in argon. TGA analysis in air shows total decomposition of all the COFs at temperatures >550 °C, thus indicating the absence of any metal residues (Supplementary Figs 26–33).

### Optical properties and photocatalysis

**Figure 5:**
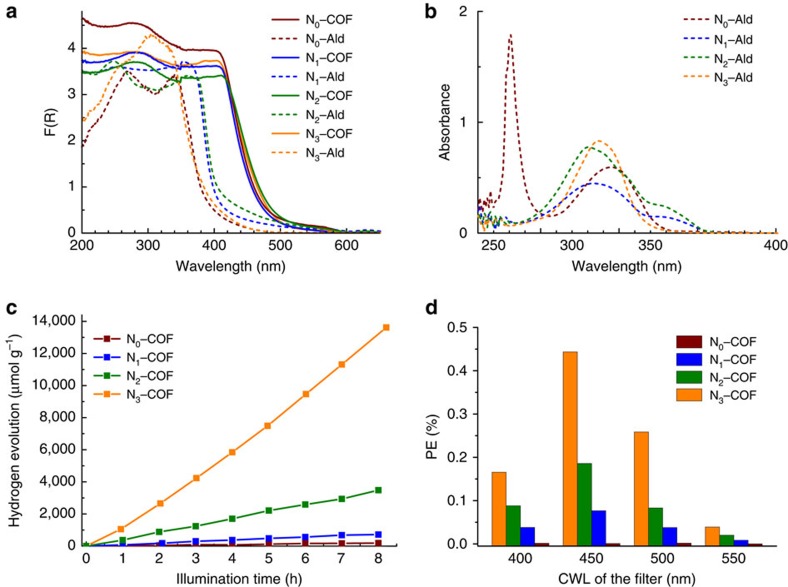
Optical and photocatalytic properties of N_*x*_–COFs. (**a**) Diffuse reflectance spectra of N_*x*_–Alds and N_*x*_–COFs recorded in the solid state. (**b**) Absorption spectra of precursor aldehydes N_*x*_–Alds in dichloromethane at 22 °C. (**c**) Hydrogen production monitored over 8 h using N_*x*_–COFs as photocatalyst in the presence of triethanolamine as sacrificial electron donor. (**d**) Photonic efficiency (PE) measured with four different band-pass filters with central wavelengths (CWLs) at 400, 450, 500 and 550 nm.

The diffuse reflectance spectrum reveals that all the COFs absorb light in the ultraviolet and blue parts of the visible region and show similar absorption profiles with an absorption edge at ∼465–475 nm (Fig. 5a), thereby suggesting an optical band gap of ≈2.6–2.7 eV as determined by the Kubelka-Munk function. These values are red shifted by 40–60 nm in comparison to the solid-state absorption spectra of the precursor aldehydes and can be attributed to the introduction of the azine group and a higher degree of conjugation resulting from delocalization along as well as across the plane in the extended frameworks^29^. As seen in Fig. 5a, unsymmetrical N_1_ and N_2_–Ald show a slightly red-shifted absorption in comparison to the symmetrical N_0_ and N_3_–Ald. DFT calculations performed on the precursor aldehydes also indicate a similar trend in the optical band gaps of symmetrical and unsymmetrical aldehydes (Supplementary Table 4). To understand the differences in absorption spectra of symmetrical and unsymmetrical aldehydes in the solid state, the absorption spectra of the precursors were additionally recorded in dilute (8 μM) dichloromethane solutions. The absorption profiles (Fig. 5b) are marked by a clear difference between the symmetrical N_3_–Ald with one absorption band and the unsymmetrical N_1_ and N_2_–Ald with twin absorption bands arising from the non-planar and hence non-fully conjugated phenyl–aryl ring systems. The two bands in the absorption spectrum of N_0_–Ald are rationalized by absorptions from the two types of ring systems that are essentially decoupled due to strong out-of-plane torsion of the peripheral phenyl rings. The increase in planarity leading to a higher degree of conjugation (hence red shift) along the series N_0_ to N_3_ is partly compensated by the increase in electron-deficient character of the central aryl ring (hence blue shift), thereby resulting in minimal changes in the optical band gap on network formation. The similar optical gap makes the N_*x*_–COF ensemble an ideal model platform for photocatalysis experiments, as their relative activities will not be governed by differences in their light-harvesting capability.

The N_*x*_–COFs were next evaluated as photocatalysts for visible light-induced hydrogen evolution. The hydrogen evolution experiments were performed by taking a suspension of the COFs in PBS at pH 7 and irradiating with visible light (≥420 nm) at 25 °C. Hexachloroplatinic acid was added for the *in situ* formation of the platinum (Pt) co-catalyst^30^ to reduce the overpotential for hydrogen evolution, and triethanolamine (TEoA) was used as sacrificial electron donor^31^,^32^.

Notably, all COFs evolve hydrogen in the test period of 8 h (Fig. 5c). Interestingly though, the N_*x*_–COFs show about fourfold increase in hydrogen evolution with each substitution of C–H by N in the central aryl ring of the COF platform. Thus, at the end of 8 h the average amount of hydrogen produced by N_0_, N_1_, N_2_ and N_3_–COF was 23, 90, 438 and 1,703 μmol h^−1^ g^−1^, respectively. The amount of hydrogen evolved from the most active N_3_–COF is competitive with carbon nitride photocatalysts and outperforms benchmark systems such as Pt-modified amorphous melon (720 μmol h^−1^ g^−1^)^33^, ‘g–C_3_N_4_' (840 μmol h^−1^ g^−1^)^34^ or crystalline poly(triazine imide) (864 μmol h^−1^ g^−1^)^33^. Photocatalysis lasting up to 48 h using N_3_–COF showed that the amount of hydrogen evolved was about four times higher than the amount of hydrogen present in the COF (Supplementary Fig. 62), thus ascertaining that the primary hydrogen source is water rather than decomposition products of the COF. After photocatalysis, the COFs were isolated and checked for stability. The FTIR (Supplementary Figs 48, 51, 54 and 57) and ssNMR (Supplementary Figs 50, 53, 56 and 59) spectra of the N_*x*_–COFs obtained after photocatalysis did not show any significant structural change in the material, thus indicating the retention of molecular connectivity of the framework during photocatalysis. The framework crystallinity was preserved to a large extent in the post-photocatalysis recovered COFs, although a partial loss of the long-range order was observed by PXRD (Supplementary Figs 49, 52, 55 and 58). Such a decrease in long-range order has been attributed to the delamination of framework layers^24^,^35^. In addition, the TEM images obtained from the post-photocatalysis sample (Supplementary Fig. 61) clearly demonstrate the retention of hexagonally ordered crystalline domains in addition to the uniform distribution of platinum nanoparticles that are formed *in situ* during photocatalysis as observed in the SEM images (Supplementary Fig. 60). Photocatalysis experiments performed in the absence of hexachloroplatinic acid did not show measurable amounts of hydrogen, thus underlining the role of platinum as electrocatalyst and microelectrode to mediate the electron transfer process^30^. Long-term studies performed with ascorbic acid as sacrificial electron donor revealed that the N_3_–COF is stable in light for over 120 h, showing sustained hydrogen evolution (Supplementary Fig. 63). To quantify the spectral distribution of the photocatalytic activity of the four COFs, the photonic efficiency (PE) was calculated using four different band-pass filters with central wavelengths at 400, 450, 500 and 550 nm (Fig. 5d; Supplementary Table 2; Supplementary Figs 64 and 65). These measurements clearly indicate that N_3_–COF shows the best PE over the entire spectral range, with the maximum of 0.44% with a 450-nm band-pass filter.

### Theoretical calculations

**Figure 6:**
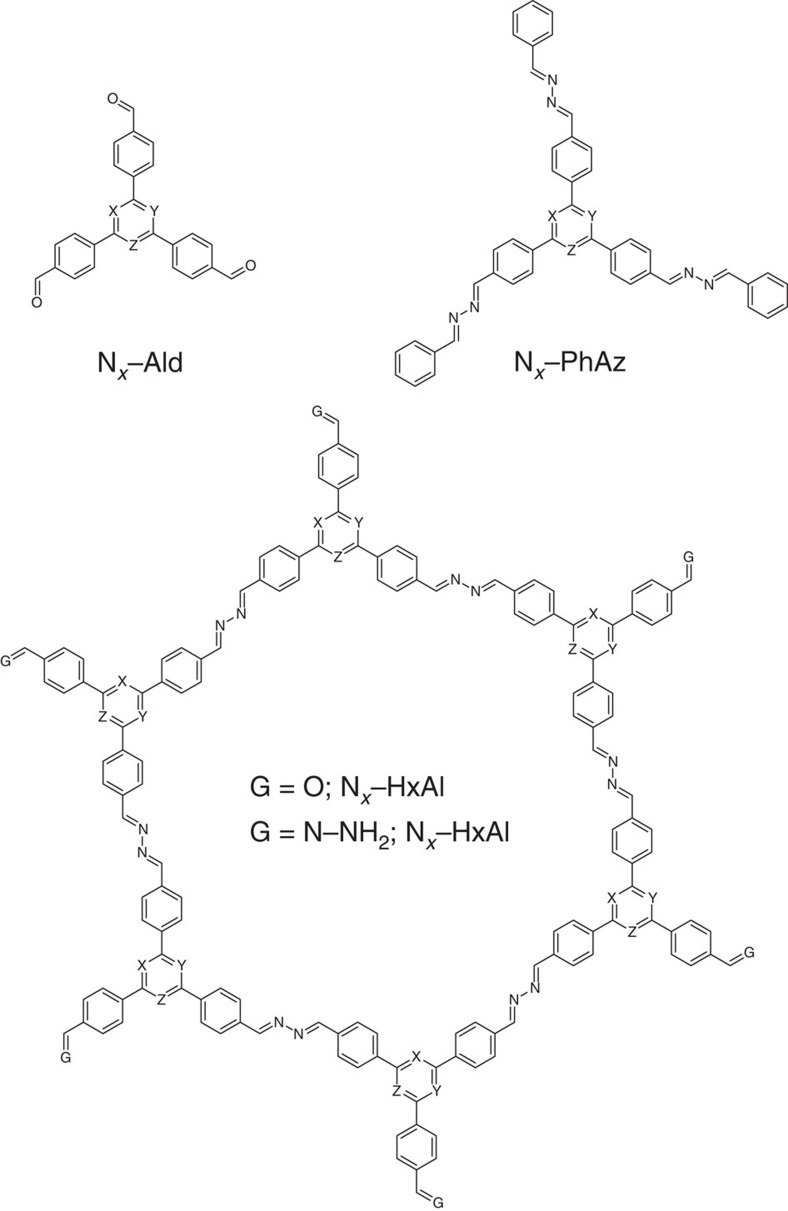
General structure of the model systems used for theoretical calculations. N_0_–: X=Y=Z=C–H. N_1_–: X=Y=C–H; Z=N. N_2_–: X=C–H; Y=Z=N. N_3_–: X=Y=Z=N.

To rationalize the observed trend and to provide insights into the change in band gaps and band positions along the **N**_*x*_ series, Kohn-Sham band gaps were calculated at the PBE0–D3/Def2–SVP level for the precursor aldehydes (N_*x*_–Ald; Supplementary Figs 66 and 67; Supplementary Table 4), model phenylazines (N_*x*_–PhAz; Supplementary Figs 68 and 69; Supplementary Table 5) and two sets of hexagons with different terminations (aldehydes N_*x*_–HxAl and hydrazones N_*x*_–HxHz; Supplementary Figs 70–73; Supplementary Tables 6 and 7), serving as representative semi-extended model systems (Fig. 6). The hexagons (N_*x*_–HxAl and N_*x*_–HxHz) were stacked up to three layers for probing the role of stacking on the band gaps and positions (Supplementary Figs 75 and 76; Supplementary Tables 8 and 9).

**Figure 7:**
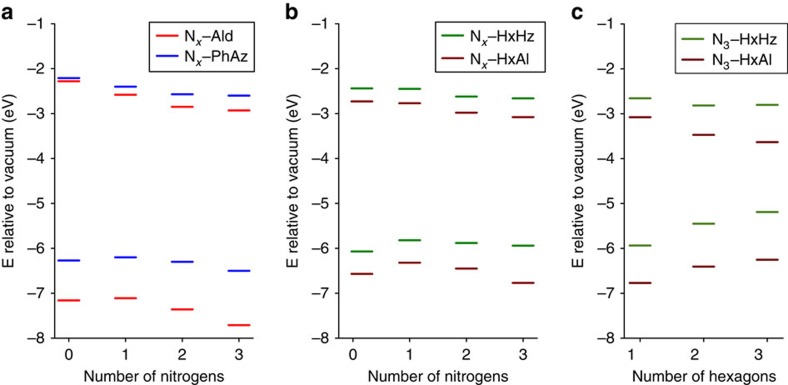
Kohn-Sham HOMO and LUMO energies of different model systems with N_*x*_ central core. (**a**) N_*x*_–Ald and N_*x*_–PhAz; (**b**) hexagons with hydrazone (N_*x*_–HxHz) and aldehyde terminations (N_*x*_–HxAl); and (**c**) stacked hexagon layers of N_3_–COF with hydrazone (N_3_–HxHz) and aldehyde terminations (N_3_–HxAl).

Kohn-Sham band gaps calculated for the oligomers (Fig. 7a,b) do not show significant variation on going from the N_0_− to the N_3_−model systems, which is in line with the experimentally observed optical spectra of the COFs that show minor differences in their absorption edge (Fig. 5a). Also, as observed by Zwijnenburg and co-workers^29^, the stacked hexagons of N_3_–COF both with hydrazone (N_3_–HxHz) and aldehyde terminations (N_3_–HxAl) show a decrease in the band gap with increasing numbers of layers (Fig. 7c).

The observed steady decrease in lowest unoccupied molecular orbital (LUMO) energy in these calculations for the N_*x*_–COFs with increasing nitrogen content suggests a decreased thermodynamic driving force for electrons to move to the platinum co-catalyst. This trend, however, does not account for the differences in the observed hydrogen evolution activities. Instead, the lowering of the highest occupied molecular orbital (HOMO) along the N_*x*_ series may lead to an increase in the oxidizing power of the hole^36^. This increase in thermodynamic driving force may therefore facilitate hole removal from the COF by the sacrificial electron donor most efficiently in N_3_–COF^37^.

In addition, molecular orbitals for the model systems were extracted and compared for the extended COF systems. Although HOMO–LUMO distributions are often invoked in the literature to rationalize exciton delocalization, charge separation and the location of potential charge-transfer sites^38^,^39^,^40^, it has to be stressed that orbitals are not observables and hence, such model considerations need to be taken with care. Also, since DFTB+ orbitals did not always agree with orbitals from DFT calculations in the case of our 7-hexagon model systems, orbital data from DFTB+ calculations may be of limited use here. Nevertheless, unit cells of the N_*x*_–COFs were optimized on the DFTB+/mio-1-0 level of theory using periodic single-point calculations, and molecular orbitals were subsequently calculated on the same level of theory. Across the different N_*x*_–COFs, the HOMO is localized solely on the azine linker unit (Supplementary Fig. 77), while the LUMO is delocalized across the conjugated -system of the framework (Supplementary Fig. 78). When associating the HOMO with the charge-transfer sites for holes, we may infer that efficient hole quenching is possible through hydrogen-bonding interactions with the sacrificial donor TEoA via the azine moiety for all COFs.

For smaller clusters calculated on a higher level of theory (PBE0–D3/Def2–SVP), we find that the HOMOs of hydrazone-terminated model hexagons are exclusively located at these terminations, while the LUMO is distributed along the hexagon with the maximum delocalization found for the planar N_3_–HxHz model (Supplementary Figs 72 and 73). To check the reliability of these model systems, even larger molecular clusters with 1,248 atoms were modelled for the N_3_ system to reduce the influence of terminating groups on the electronic structure of the innermost aryl core. However, the terminal hydrazone moieties still strongly localize the HOMO, whereas the LUMO in the 7-hexagon system is still centred on the innermost hexagon, despite the vicinal hexagons (Supplementary Fig. 74). This suggests an efficient HOMO–LUMO separation for oligomers with electron-rich terminal groups, which could assist the charge separation process on excitation. Note, however, that the HOMO and LUMO do not necessarily correspond to the orbitals participating in the brightest transitions and further calculations are necessary to pinpoint the orbital contributions to the optical absorptions involved in the photocatalytic process.

**Figure 8:**
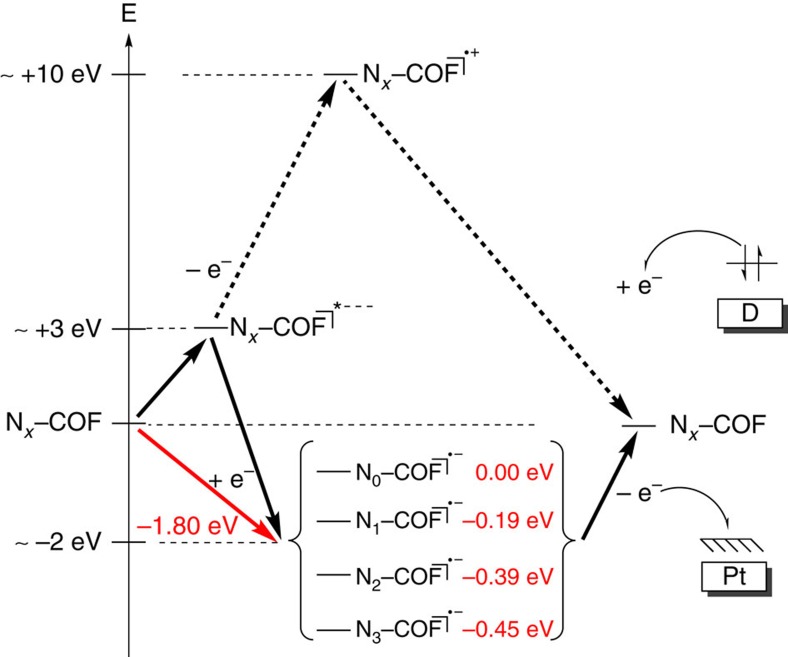
Schematic representation of two possible pathways after photoexcitation of N_*x*_–COFs. Quenching the hole on the COF by the sacrificial electron donor leads to a radical anionic state for the COF (radical anion pathway, red arrow). The opposite order leads to the radical cationic pathway (dotted black arrows). Energies in red depict calculated vertical electron affinities as differences in total energies between radical anionic and neutral states of N_*x*_–HxHz model systems at PBE0–D3/Def2–SVP level. Asterisk (*) denotes the excited state.

Since orbital considerations as non-observables are always difficult as discussed above, an alternative approach was pursued in considering the possible reaction intermediates. The stability of reactive intermediates formed during the photocatalytic process appears as a more reliable descriptor to rationalize the observed trend in the photocatalytic activity across the series of COFs. Hence, the energies and relative stabilities of radical anions of hydrazone-terminated N_*x*_–hexagons were calculated as outlined in Fig. 8.

Electron affinities of the N_*x*_-systems in the order of −2 eV were computed (Supplementary Table 10), whereas the ionization potential for N_3_–HxHz is estimated in the regime of roughly +10 eV in vacuum, thus rendering the formation of a radical cation during the photocatalytic process less likely. Considering the formation of a radical anion as the rate-determining step, which is most favoured for the N_3_ system and least facile for the N_0_ system, the observed energetics of the radical anion upholds the observed trend. On one hand, this finding emphasizes the role of the sacrificial electron donor that needs to swiftly remove the hole from the COF leading to the formation of a radical anion. On the other hand, it underlines the importance of the electron-poor character of the triazine building block, which is efficient at stabilizing the negative charge generated on the COF and at transferring it to the nearest platinum site. This trend is fully in line with the observed hydrogen evolution activity and we therefore reason that the formation of the radical anion is crucial for the photocatalytic process, as exemplified by N_*x*_–COFs.

## Discussion

Although the above calculations are fully in line with the observed exponential increase in hydrogen production across the N_*x*_-series, the latter cannot be pinpointed to a single property change of the COFs but rather has to be ascribed to a complex interplay of several factors. For example, an increase in surface area may play a key role in the photocatalytic activity by providing a greater number of exposed active sites. However, in the present case, only a weak correlation with the surface area of the N_*x*_–COFs could be established, thus suggesting that surface area is not the central factor determining the photocatalytic activity (Supplementary Table 3).

Further, as observed earlier^29^, stacking likely plays a key role in the observed red shift of absorption spectra of the COFs as compared with the spectra of the aldehyde building blocks. In addition, as a consequence of the decreased dihedral angle along the series and hence improved crystallinity, enhanced structural definition and layer registry, most facile exciton migration within the COF plane and also along the well-stacked aryl rings is expected for N_3_–COF, which is in line with the observed trend in photocatalytic activity^41^,^42^. Although the exciton dynamics in such materials is expected to be complicated and dependent on both structural and electronic factors, it is interesting that a logarithmic plot of dihedral angles obtained from the geometry optimized precursor aldehydes against hydrogen production of the respective COFs shows a linear relationship (Supplementary Fig. 79). Regarding electronic factors, the computed increase in stabilization of the radical anions in the most nitrogen-rich COFs nicely correlates with the observed trend. The stabilization of the anion radical likely enhances the charge separation, thereby increasing the probability of successful electron migration to a nearby Pt co-catalyst. The relevance of the anion radical for hydrogen evolution likewise shifts the focus to the sacrificial electron donor, whose interaction with the COF likely determines how quickly the hole can be quenched. Therefore, our results suggest that tuning the interfacial interactions between the COF and the electron donor may be a promising route to optimize the hydrogen evolution efficiencies further in such systems.

In summary, we have synthesized a new COF platform that can be tuned for visible light-induced hydrogen evolution from water. Systematic variation in the properties of our four structurally related COFs clearly indicates that tuning the electronic and structural properties of the precursors has a significant impact on the photocatalytic activity of the resulting COFs. We have thus shown that engineering the building blocks and, hence, the electronic properties of photofunctional COFs opens new avenues to tunable, tailor-made supramolecular photocatalysts. Hydrogen evolution resulting from a crystalline COF that retains its structure during photocatalysis is an important stepping stone on the way to full water splitting by organic frameworks with embedded photophysical functions, and at the same time holds much room for further improvement through rational band gap and catalytic site engineering by tailoring the molecular building blocks.

## Methods

### General synthesis of COFs

All COFs were prepared by a procedure identical to the one described here for the synthesis of N_3_–COF. In a Biotage 5-ml high precision glass vial, N_3_–Ald (50 mg, 0.13 mmol) was suspended in a mixture 1.0 ml mesitylene, 1.0 ml 1,4-dioxane and 100 μl aqueous 6 M acetic acid. To the suspension, hydrazine hydrate (10 μl, 50–60% solution, Sigma-Aldrich) was then added. The vial was then sealed and heated in an oil bath at 120 °C for 3 days at autogenous pressure. Thereafter, the vial was opened and the suspension was filtered and washed with chloroform (2 × 5 ml), acetone (2 × 5 ml) and tetrahydrofuran (2 × 5 ml). The solid was dried in an oven at 60 °C to afford N_3_–COF as a light yellow powder. Anal. Calcd. for (C_24_N_6_H_15_)_n_: C, 74.40; N, 21.69; H, 3.90. Found: C, 72.36; N, 21.32; H, 4.07. ^13^C CP–MAS NMR (100 MHz) *δ* p.p.m. 168.39, 159.55, 136.61 and 126.96.

### Photocatalysis

All photocatalysis experiments were performed in a double-walled glass reactor, where the outer compartment is circulated with water kept at a constant temperature (25 °C) through a thermostat. The reactor was top irradiated through a quartz window with a xenon lamp (Newport, 300 W) equipped with a water filter and a dichroic mirror (900 nm>*λ*>420 nm). For each experiment, the photocatalyst (COF; 5 mg) was suspended in PBS (10 ml of 0.1 M solution at pH 7) containing TEoA (100 μl; 0.738 mmol). For long-term experiments, ascorbic acid was used instead of TEoA. Hexachloroplatinic acid (5 μl, 8 wt% aqueous solution, Sigma-Aldrich) was added for the *in situ* formation of platinum as the co-catalyst. The actual loading with Pt as determined by the inductively coupled plasma optical emission spectroscopy (ICP-OES) analysis of COFs after photocatalysis was 2.14, 1.70, 0.94 and 0.68 wt% for N_0_, N_1_, N_2_ and N_3_–COF, respectively. The head space was subjected to several cycles of evacuation and argon backfill before the experiment. In the course of the experiment, the head space of the reactor was periodically sampled and the components were quantified by gas chromatography (Thermo Scientific TRACE GC Ultra) equipped with a thermal conductivity detector (TCD) detector using argon as the carrier gas. For long-term photocatalysis experiments, the head space of the reactor was evacuated and purged with argon every 24 h to avoid hydrogen buildup and the photocatalysis was resumed. After the photocatalysis experiment, the COFs were recovered by filtration, washed with water and then dried at 100 °C. The PE of the photocatalysts were determined under irradiation using band-pass filters with central wavelengths (400, 450, 500 and 550 nm; Thorlabs). For this purpose, 10 mg of COF was suspended in buffer (PBS, 10 ml of 0.1 M solution at pH 7) containing TEoA (1,000 μl; 7.38 mmol) and hexachloroplatinic acid (10 μl, 8 wt% aqueous solution, Sigma-Aldrich). The power of the incident light was obtained from a thermo power sensor (Thorlabs). The PE was then calculated using the equation PE=2·[H_2_]/*I*, where [H_2_] is the average hydrogen evolution rate and *I* is the incident photon flux.

### Characterization

Infrared spectra were recorded in attenuated total reflection (ATR) geometry on a PerkinElmer UATR Two equipped with a diamond crystal. The spectra were background corrected. Raman spectra were recorded with a Jobin Yvon-type V 010 labRAM single-grating spectrometer equipped with a double super razor edge filter and a peltier-cooled charge-coupled device (CCD) camera in quasi-backscattering geometry using the linearly polarized 632.817-nm He/Ne gas laser. Diffuse reflectance UV–visible absorption spectra were collected on a Cary 5000 spectrometer (referenced to barium sulphate). Absorption spectra were calculated from the reflectance data using the Kubelka-Munk function. The liquid state ^1^H and ^13^C NMR spectra were recorded on a Bruker 300-MHz NMR spectrometer, while the ssNMR was recorded on a Bruker 400- and 500-MHz spectrometer. For ssNMR spectroscopy, the sample was filled in a 2.5-mm ZrO_2_ rotor, which was mounted in a standard double resonance MAS probe (Bruker). The ^13^C chemical shift was referenced relative to tetramethylsilane. The ^1^H–^13^C CP–MAS spectra were recorded at a spinning speed of 20 kHz, using a ramped-amplitude CP pulse on ^1^H. Argon sorption measurements were performed at 87 K with a Quantachrome Instruments Autosorb-iQ. The pore-size distribution was calculated from Ar adsorption isotherms by non-local DFT using the ‘Ar–zeolite/silica cylindrical pores at 87 K' kernel (applicable pore diameters 3.5–1,000 Å). PXRD data were collected on a Huber G670 diffractometer in Debye-Scherrer geometry using Ge(111)-monochromatized Cu–K*α* radiation (*λ*=1.5406 Å). CHN elemental analyses were performed with a vario EL elemental analyser (Elementar Analysensysteme GmbH). SEM images were obtained either by a VEGA TS 5130MM (TESCAN) or with a Zeiss Merlin with EsB (energy and angle selective BSE) and SE (secondary electron) detector. TEM was performed with a Philips CM30 ST (300 kV, LaB_6_ cathode). The samples were suspended in *n*-butanol and drop-cast onto a lacey carbon film (Plano). TGA measurements were performed on NETZSCH STA 409 C/CD at a heating rate of 5 K min^−1^ under argon and in air. For XPS, samples were pressed onto indium foil and the spectra were collected on an Axis Ultra (Kratos Analytical, Manchester) X-ray photoelectron spectrometer with charge neutralization. The spectra were referenced with the adventitious carbon 1-s peak at 284.80 eV.

### Calculations

Structures for all investigated building blocks were optimized on the PBE0–D3/Def2–SVP level of theory. Kohn-Sham band gaps were obtained from single-point calculations on the same level of theory. Excitation energies for optimized building blocks were calculated on the TD–PBE0–D3/Def2–SVP level of theory. Excitations with the largest oscillator strength were selected for each optimized building block to compute the different densities. Calculations for precursor aldehydes and model compounds were done using the Turbomole program package in version 6.3.1 (ref. 43). Calculations for the hexagons with different terminations were done using the FermiONs++ program package^44^,^45^. Periodic calculations for optimizations and single points were performed using the DFTB+ program package^46^.

## Additional information

**How to cite this article:** Vyas, V. S. *et al*. A tunable azine covalent organic framework platform for visible light-induced hydrogen generation. *Nat. Commun.* 6:8508 doi: 10.1038/ncomms9508 (2015).

## Supplementary Material

Supplementary Information Supplementary Figures 1-80, Supplementary Tables 1-10, Supplementary Methods and Supplementary References

## Figures and Tables

**Table 1:**
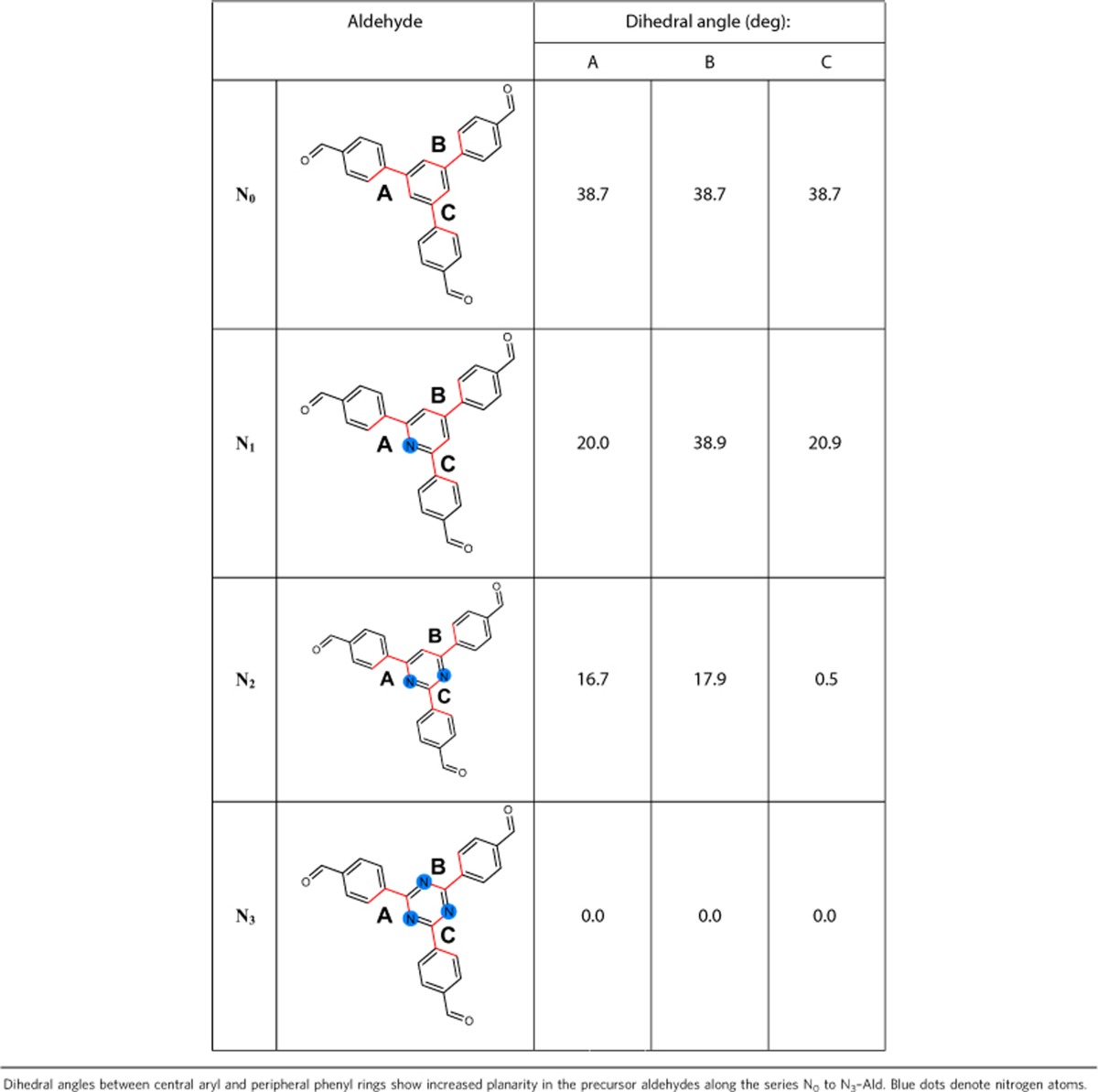
DFT geometry optimizations of precursor aldehydes at the PBE0–D3/Def2–SVP level.
