# Supplementary material for: A tunable azine covalent organic framework platform for visible light-induced hydrogen generation
Source: Nat Commun. 2015 Sep 30;6:8508. doi: 10.1038/ncomms9508 (PMC4598847; doi:10.1038/ncomms9508)
Supplement: Supplementary Information — Supplementary Figures 1-80, Supplementary Tables 1-10, Supplementary Methods and Supplementary References [file ncomms9508-s1.pdf]

1. Supplementary Figures

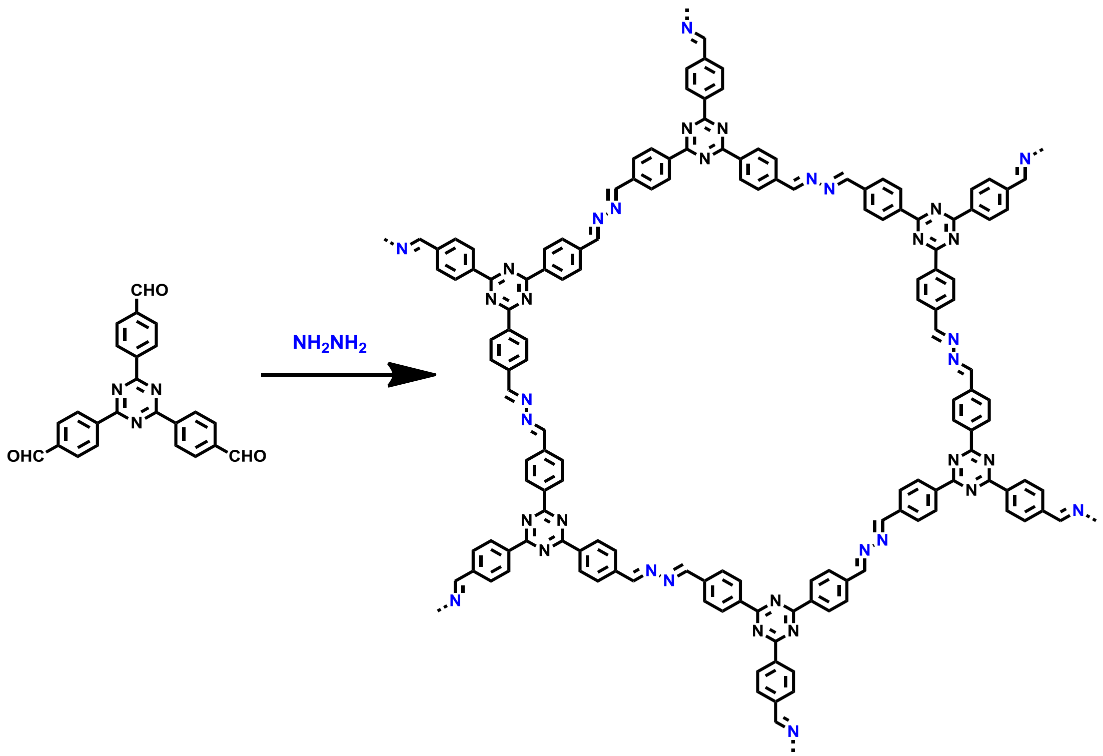

Supplementary Figure 1: Synthesis scheme for N_3-COF.

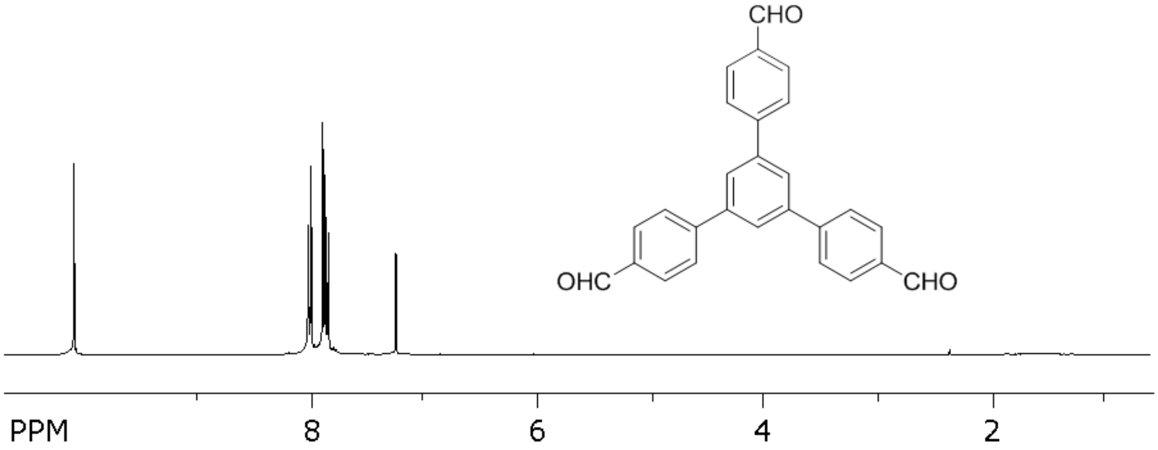

Supplementary Figure 2: ^{1}H NMR of 1,3,5-Tris(4-formylphenyl)benzene (**N_0-Ald**).

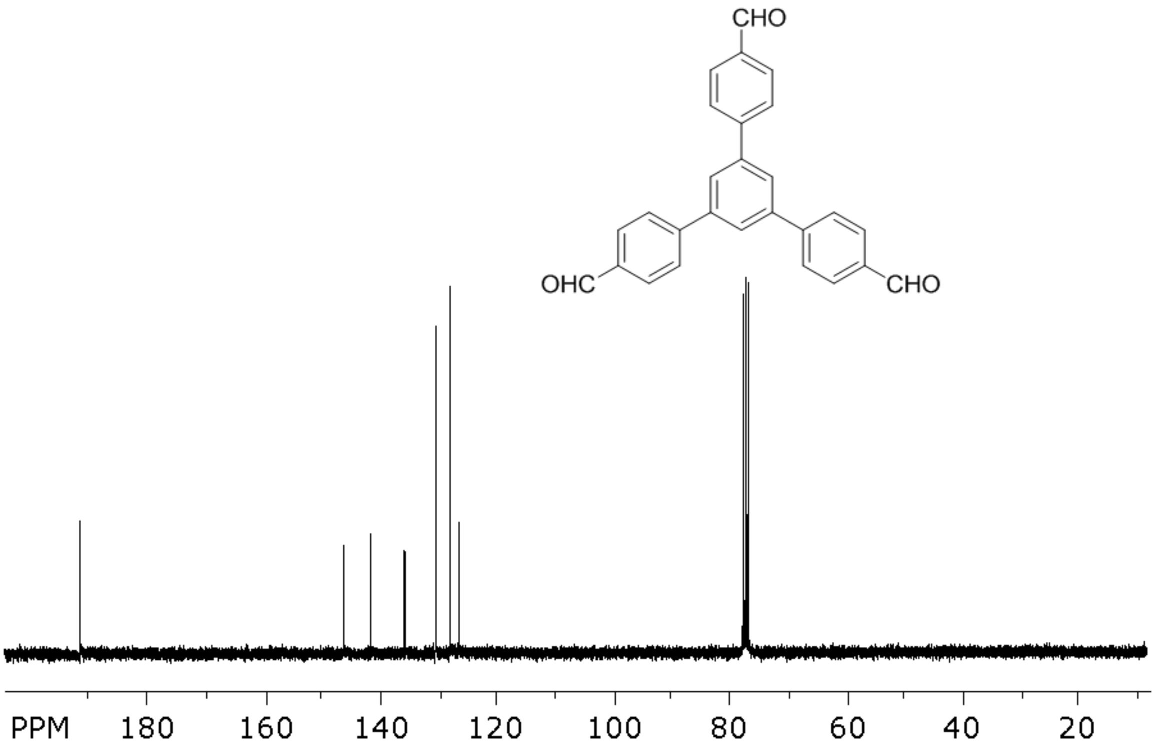

Supplementary Figure 3: ^{13}C NMR of 1,3,5-Tris(4-formylphenyl)benzene (**N_0-Ald**).

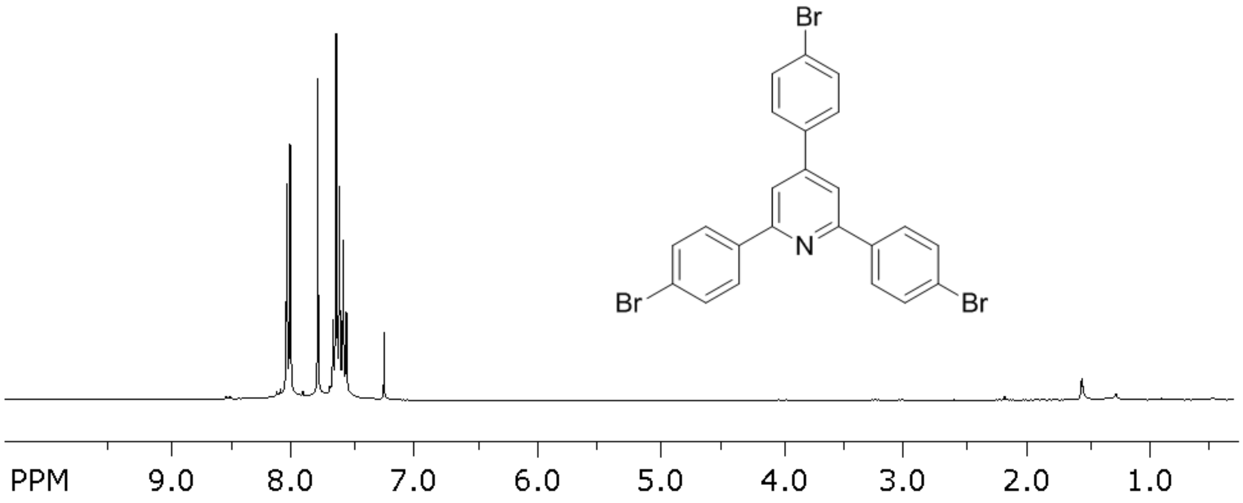

Supplementary Figure 4: ^{1}H NMR of 2,4,6-Tris(4-bromophenyl)pyridine (**1**).

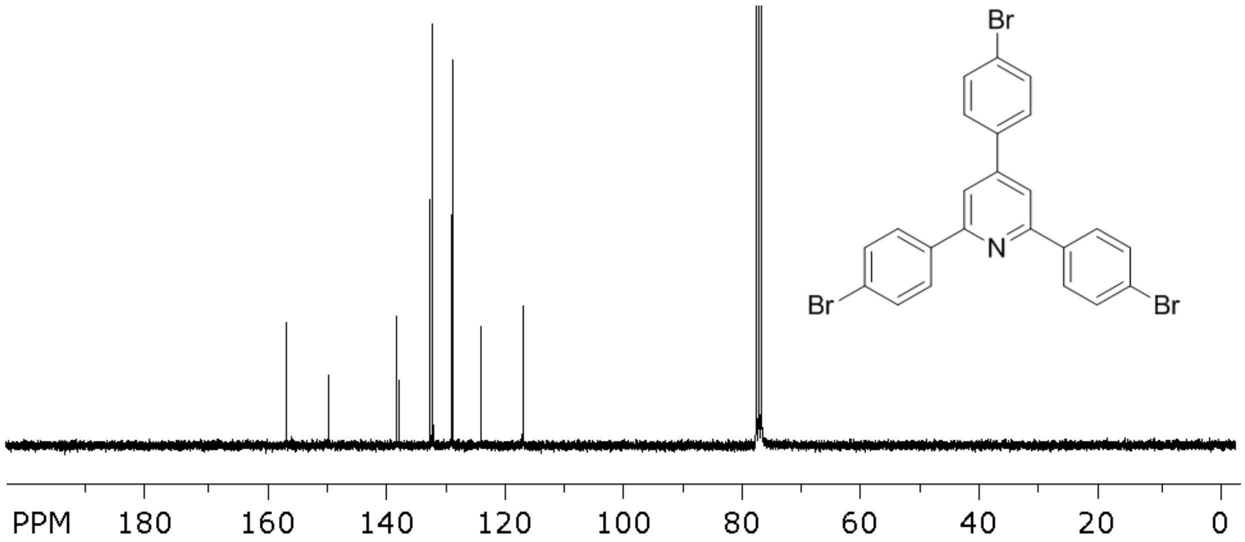

Supplementary Figure 5: ^{13}C NMR of 2,4,6-Tris(4-bromophenyl)pyridine (**1**).

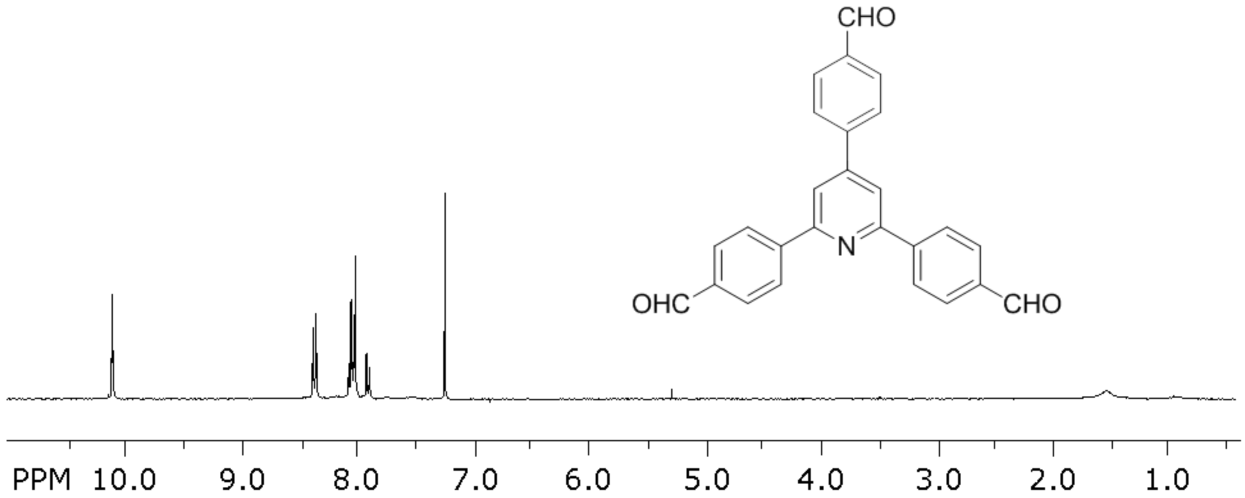

Supplementary Figure 6: ^{1}H NMR of 2,4,6-Tris(4-formylphenyl)pyridine ($\mathbf{N_1}$**-Ald**).

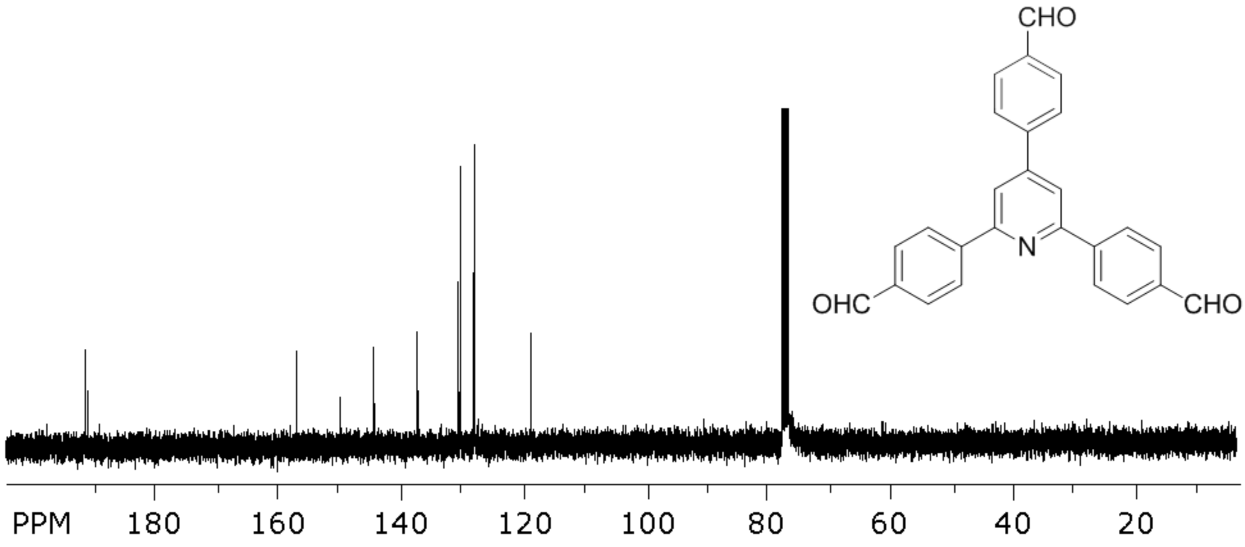

Supplementary Figure 7: ^{13}C NMR of 2,4,6-Tris(4-formylphenyl)pyridine (**N_1-Ald**).

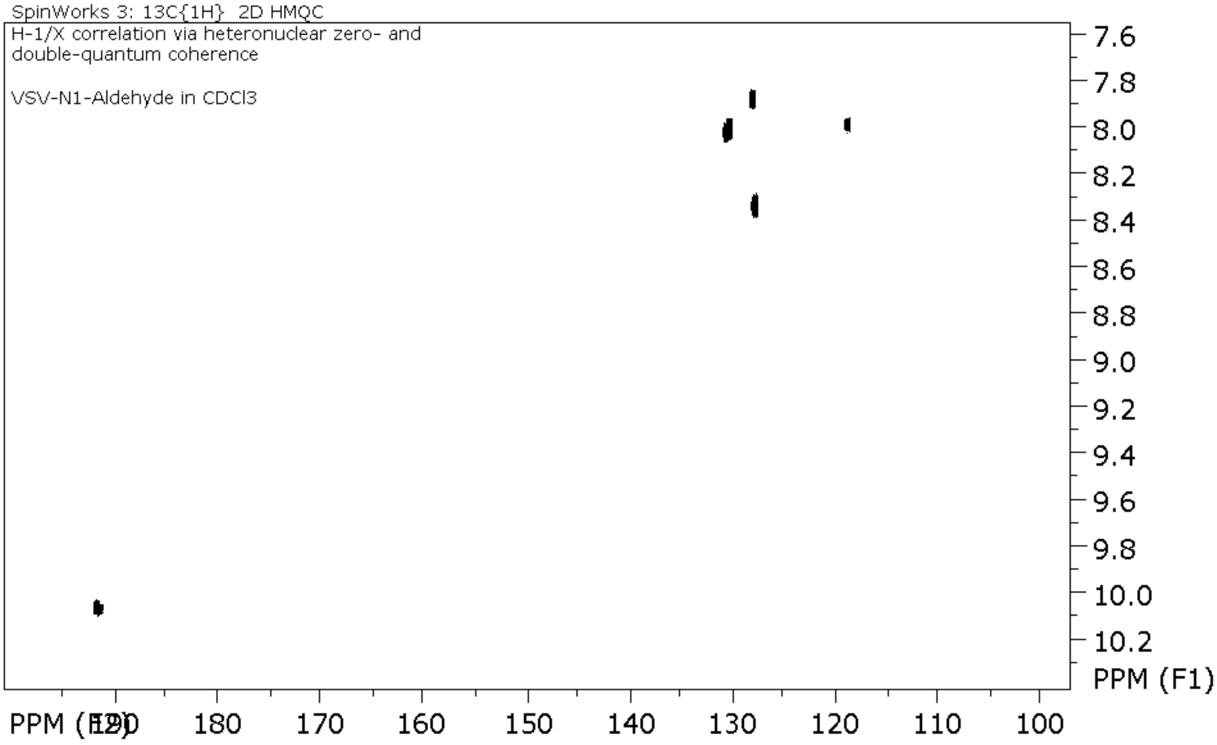

Supplementary Figure 8: 2D HMQC NMR of 2,4,6-Tris(4-formylphenyl)pyridine (**N_1-Ald**).

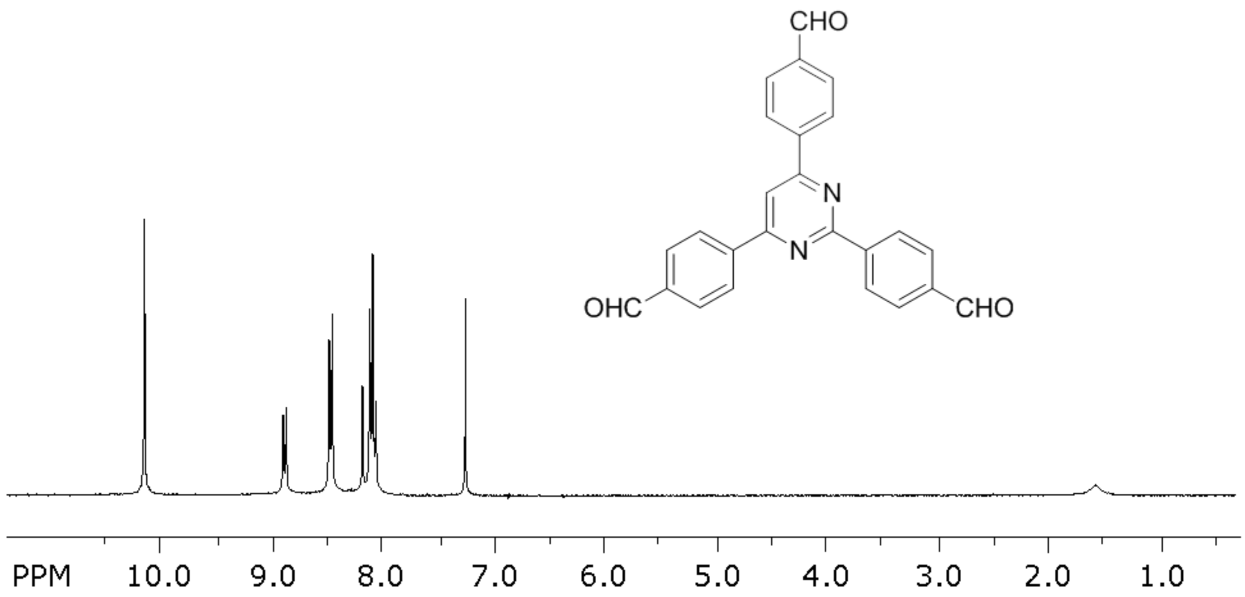

Supplementary Figure 9: ^{1}H NMR of 2,4,6-Tris(4-formylphenyl)pyrimidine (**N$_2$-Ald**).

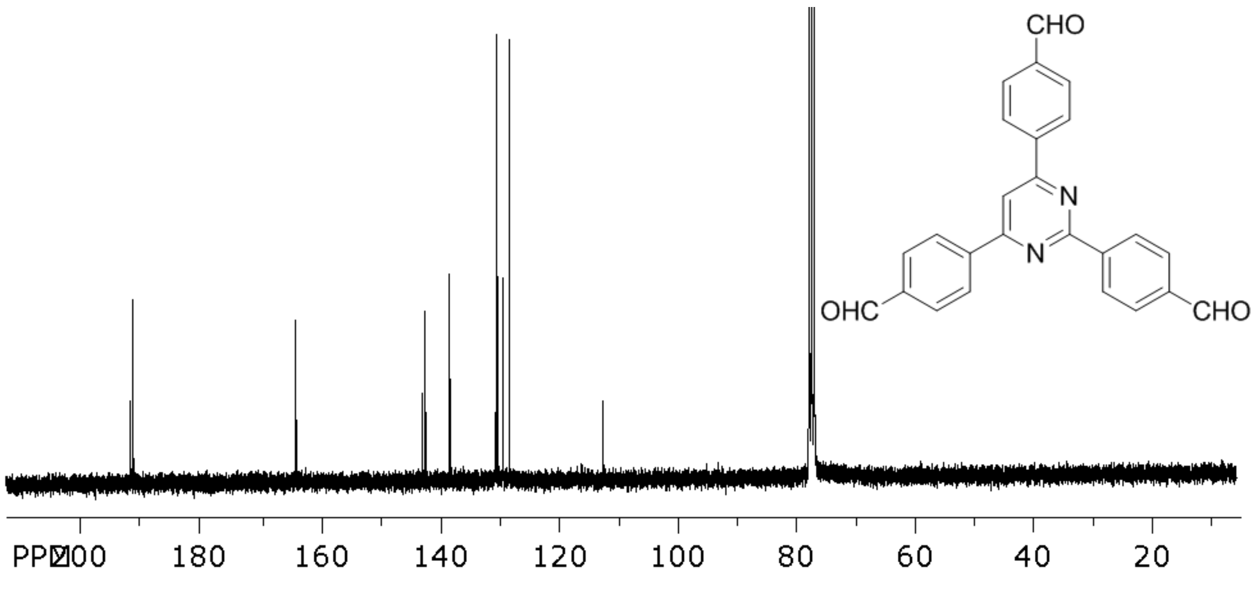

Supplementary Figure 10: ^{13}C NMR of 2,4,6-Tris(4-formylphenyl)pyrimidine (**N$_2$-Ald**).

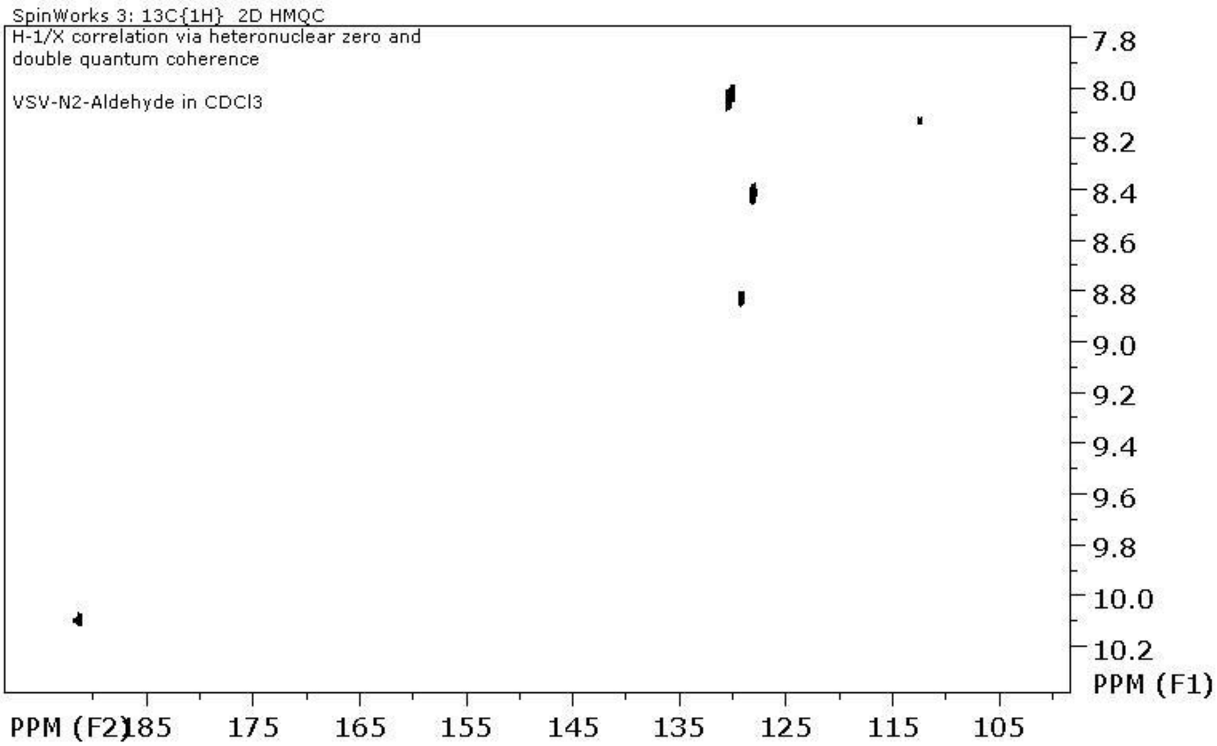

Supplementary Figure 11: 2D HMQC NMR of 2,4,6-Tris(4-formylphenyl)pyrimidine (**N_2-Ald**).

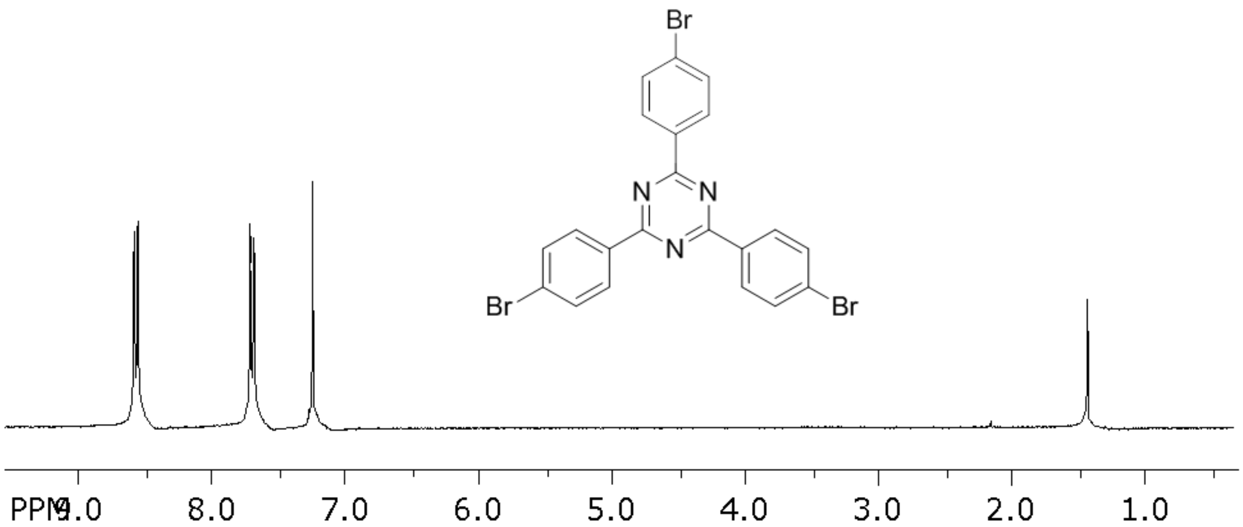

Supplementary Figure 12: ^{1}H NMR of 2,4,6-Tris(4-bromophenyl)-1,3,5-triazine (**2**).

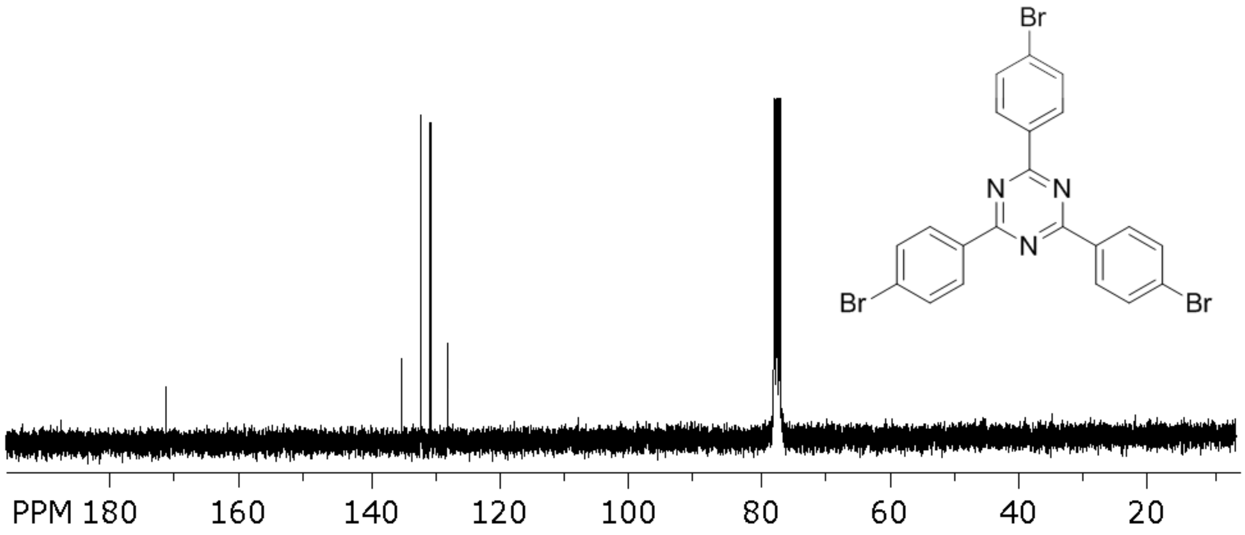

Supplementary Figure 13: ^{13}C NMR of 2,4,6-Tris(4-bromophenyl)-1,3,5-triazine (**2**).

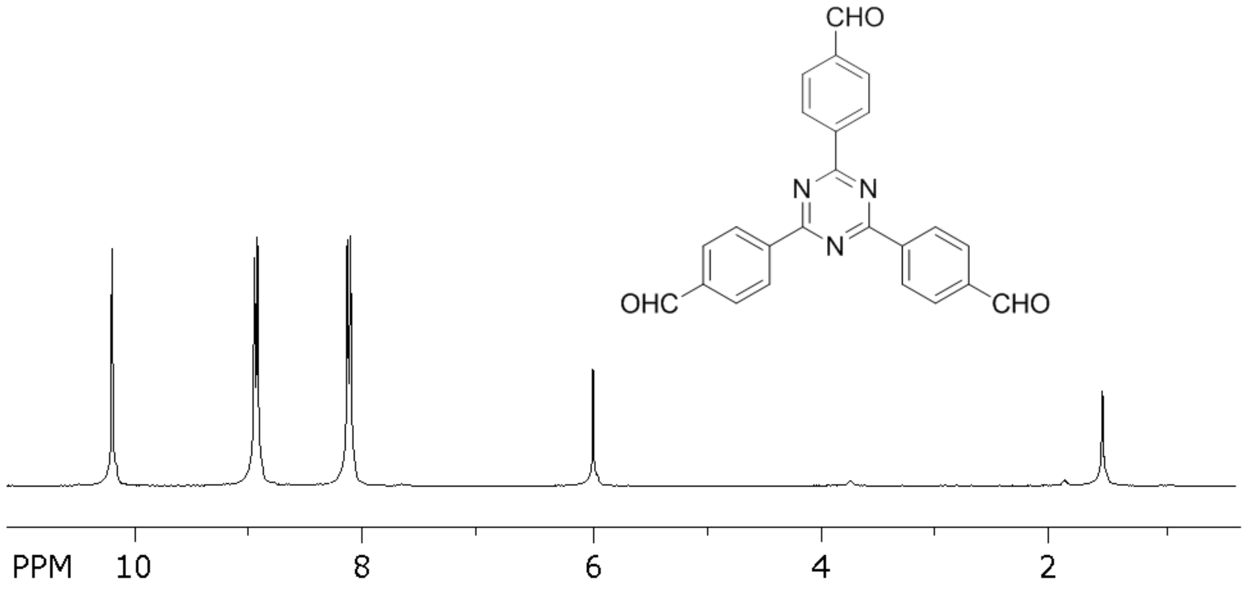

Supplementary Figure 14: ^{1}H NMR of 2,4,6-Tris(4-formylphenyl)-1,3,5-triazine (**N_3-Ald**).

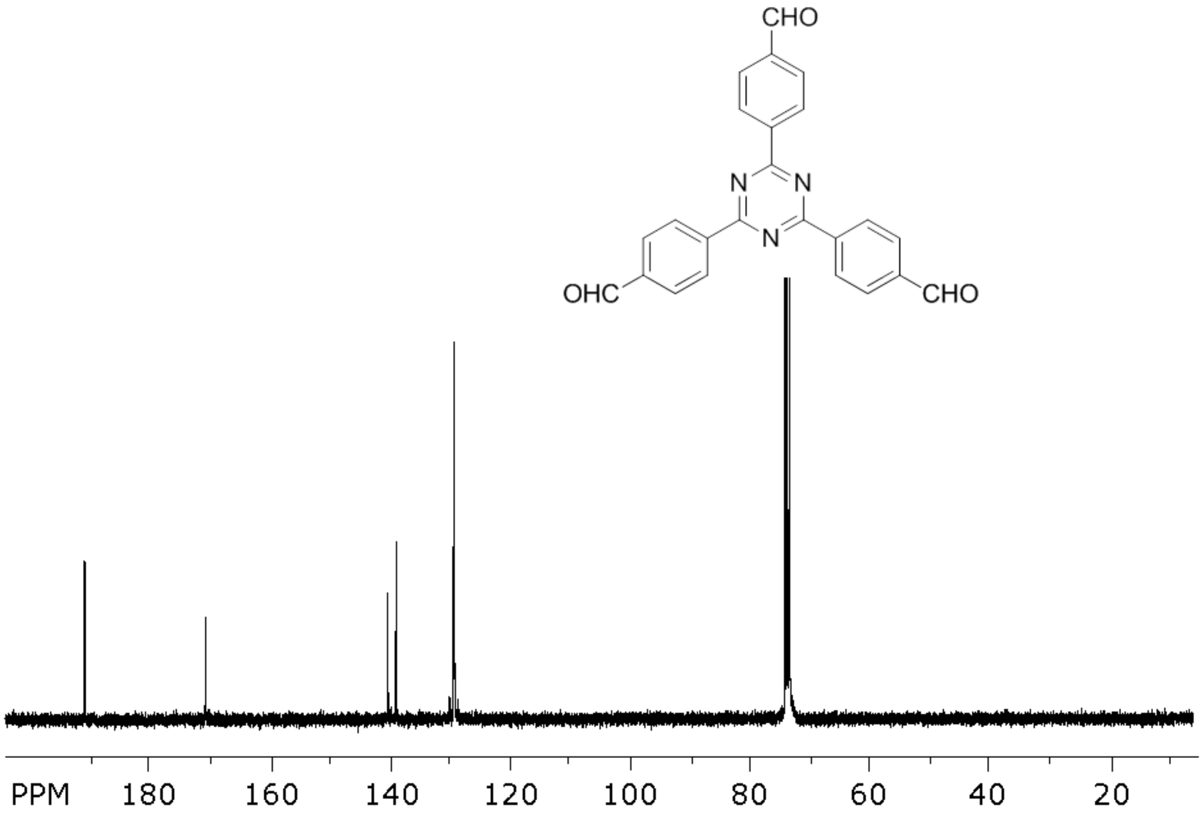

Supplementary Figure 15: ^{13}C NMR of 2,4,6-Tris(4-formylphenyl)-1,3,5-triazine (**N_3-Ald**).

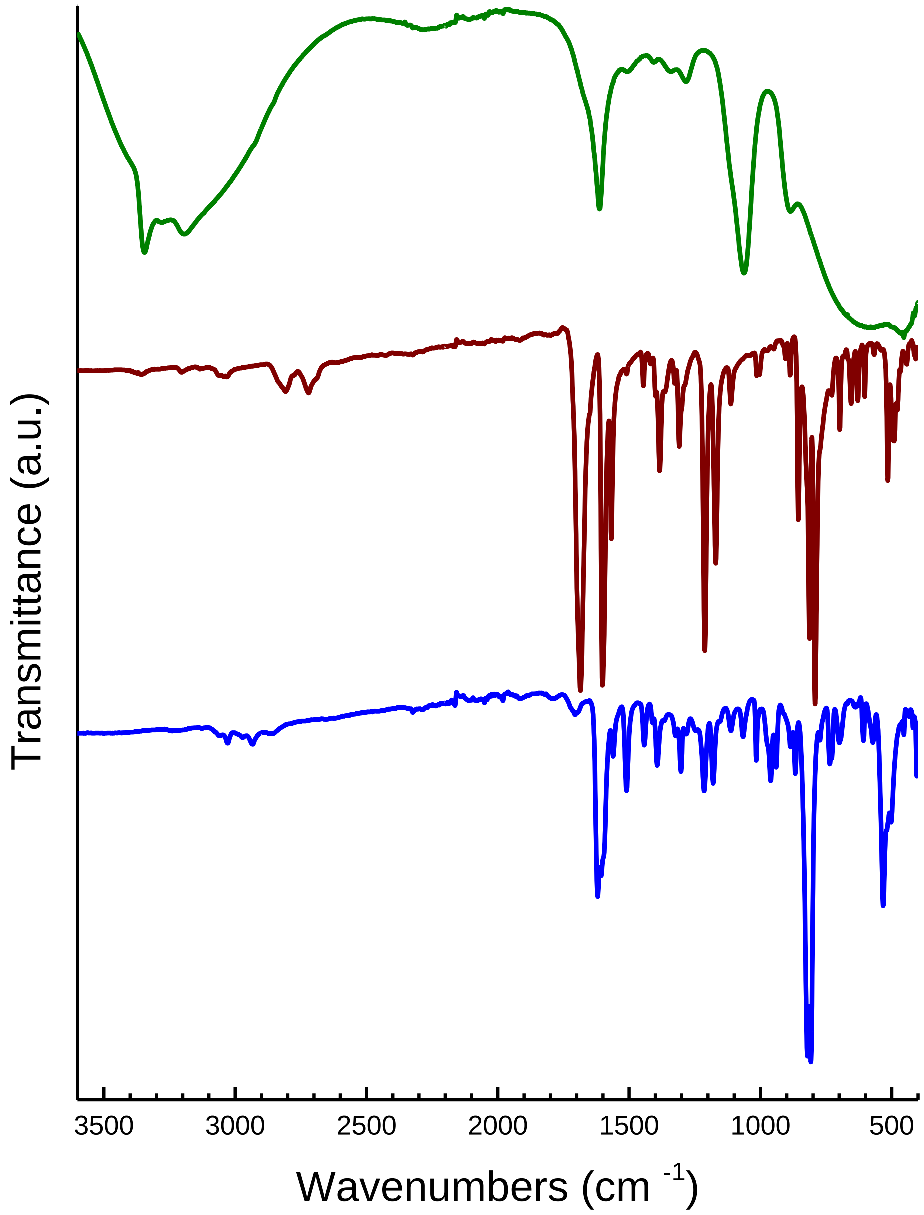

Supplementary Figure 16: FT-IR spectra of hydrazine hydrate (green), **N_0-Ald** (brown) and **N_0-COF** (blue).

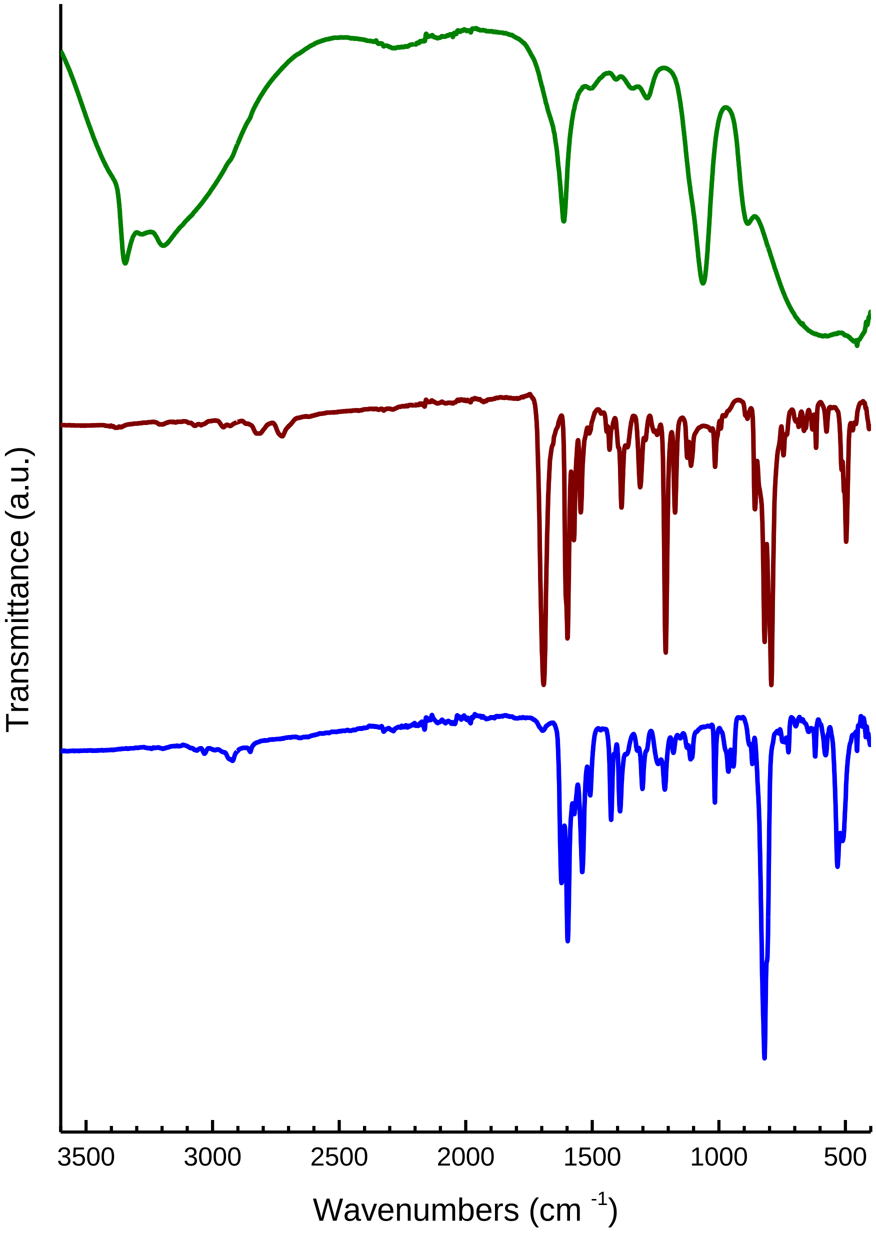

Supplementary Figure 17: FT-IR spectra of hydrazine hydrate (green), **N_1-Ald** (brown) and **N_1-COF** (blue).

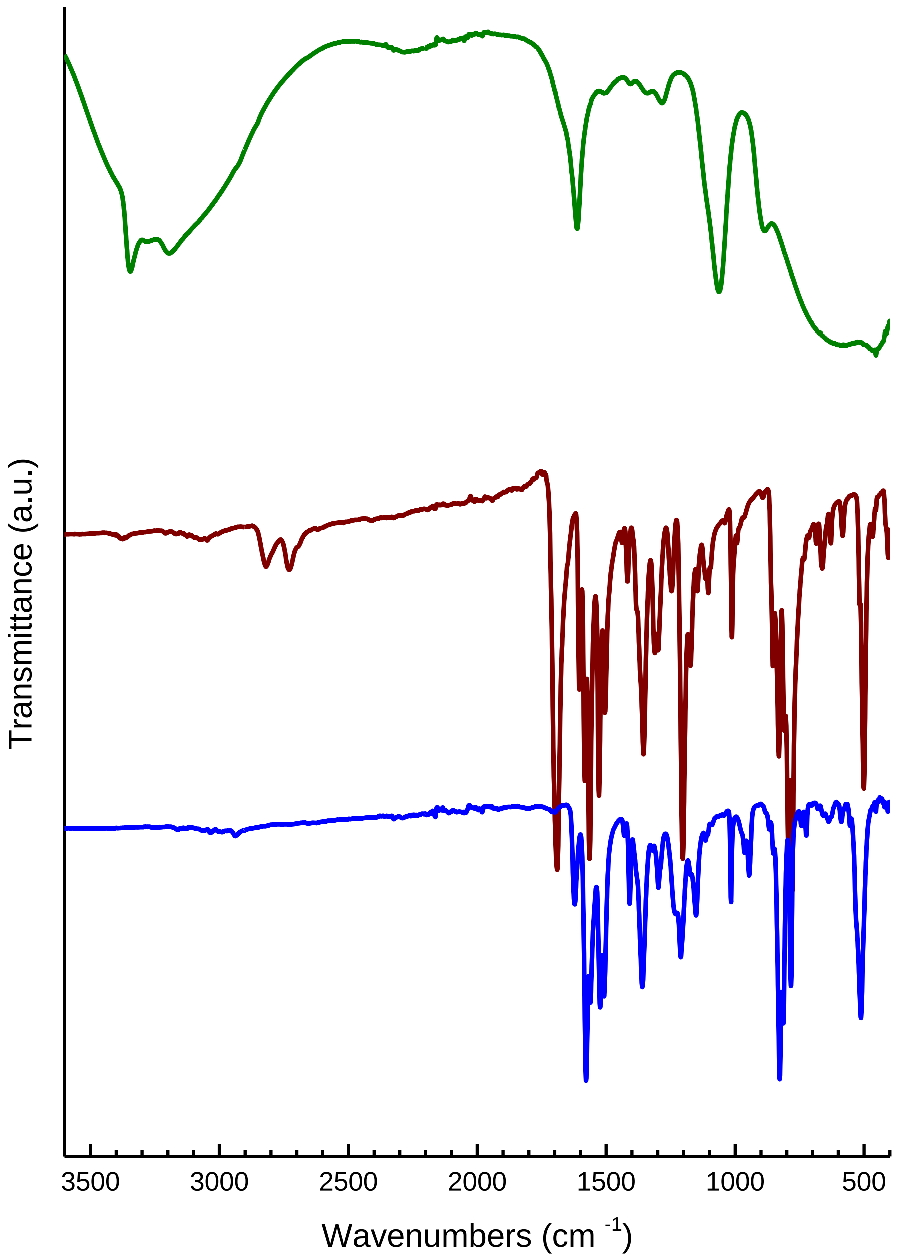

Supplementary Figure 18: FT-IR spectra of hydrazine hydrate (green), **N_2-Ald** (brown) and **N_2-COF** (blue).

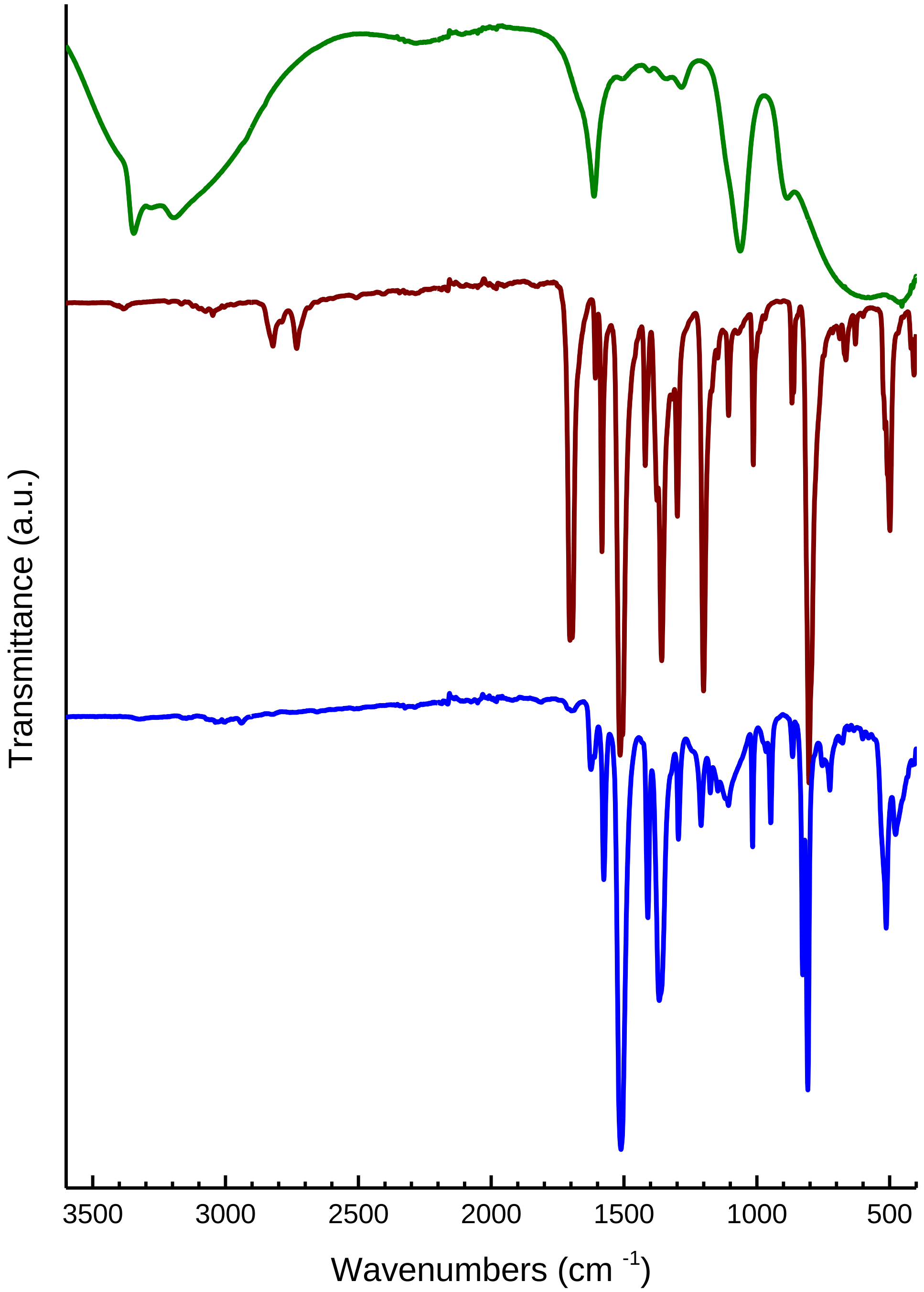

Supplementary Figure 19: FT-IR spectra of hydrazine hydrate (green), **N_3-Ald** (brown) and **N_3-COF** (blue).

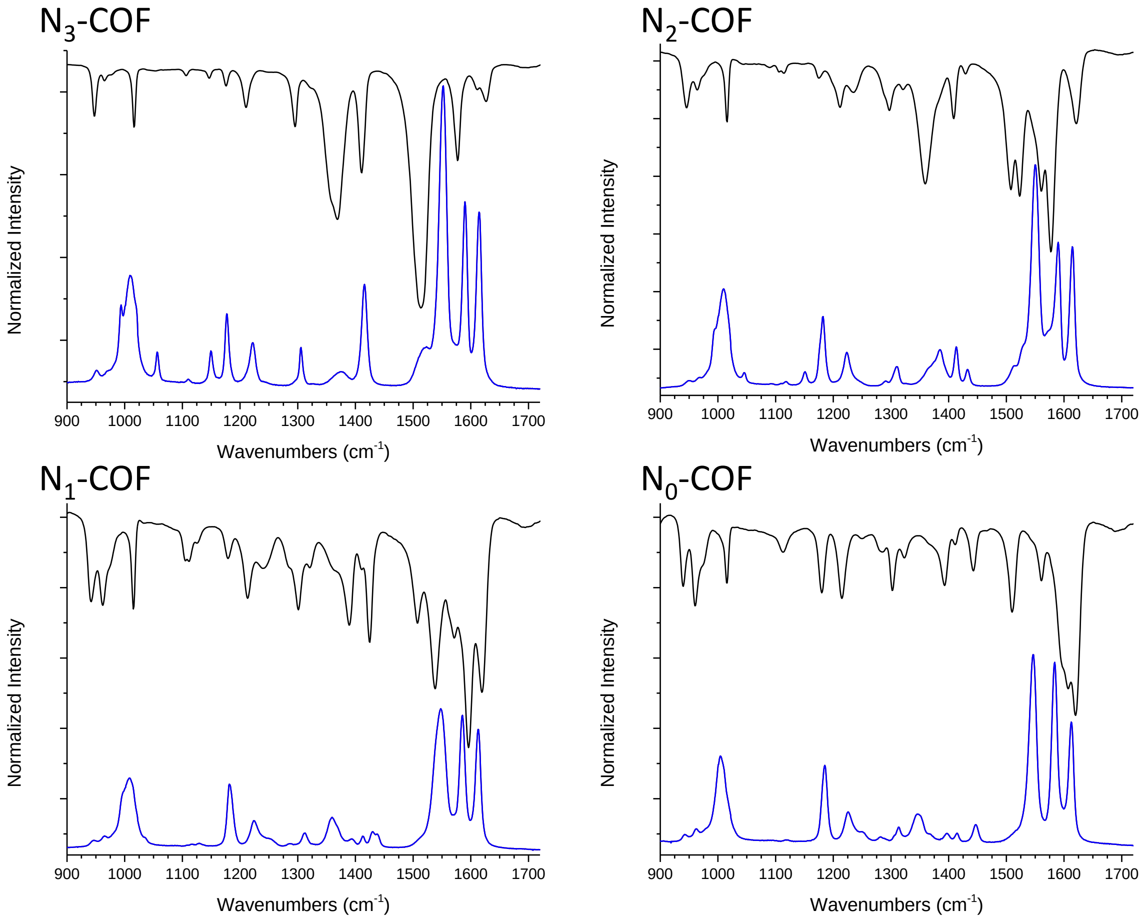

Supplementary Figure 20: A comparison of the Raman (blue) and FT-IR (black) spectra of **N$_x$-COFs**.

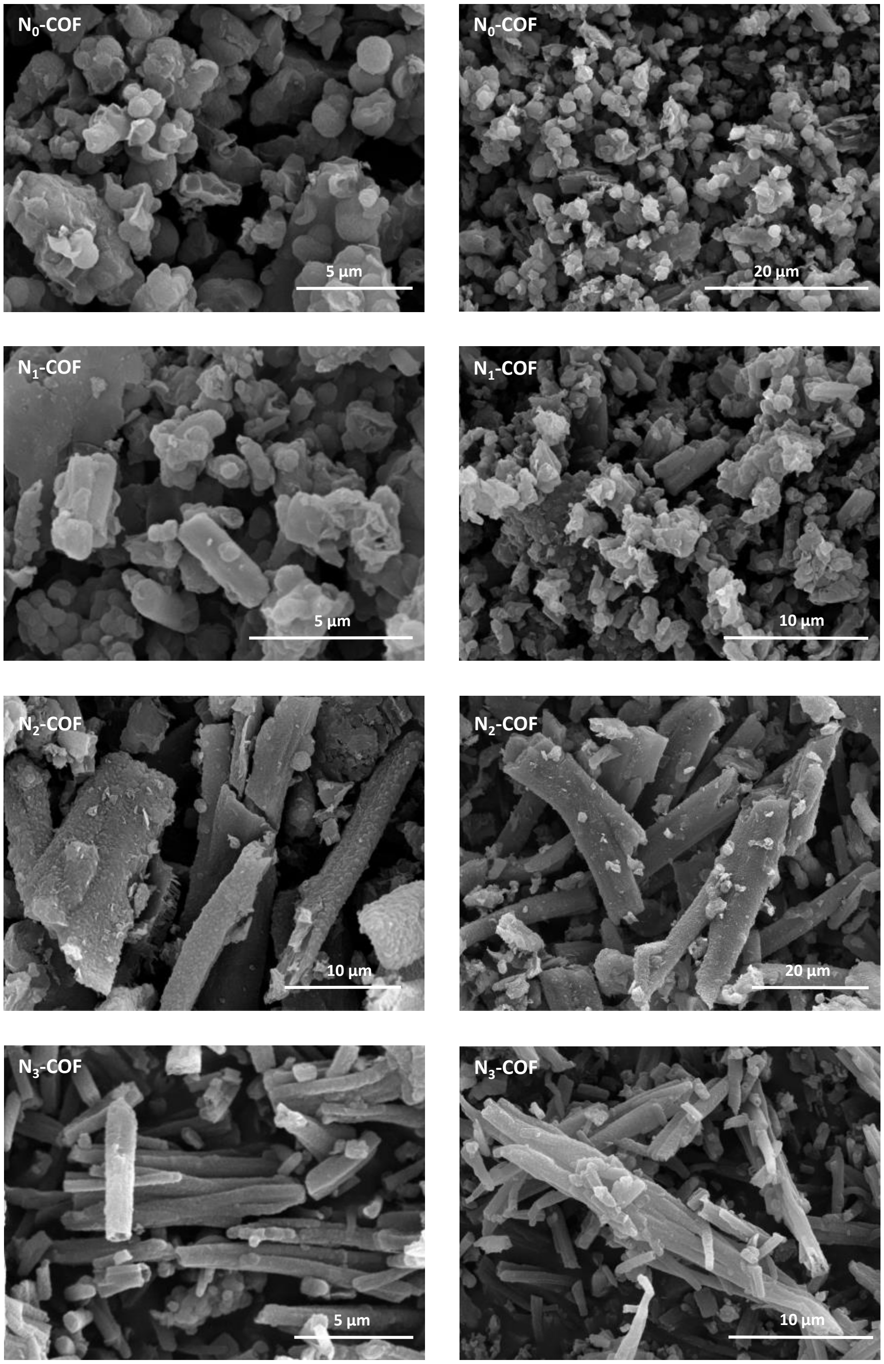

Supplementary Figure 21: SEM images of **N_x-COFs**.

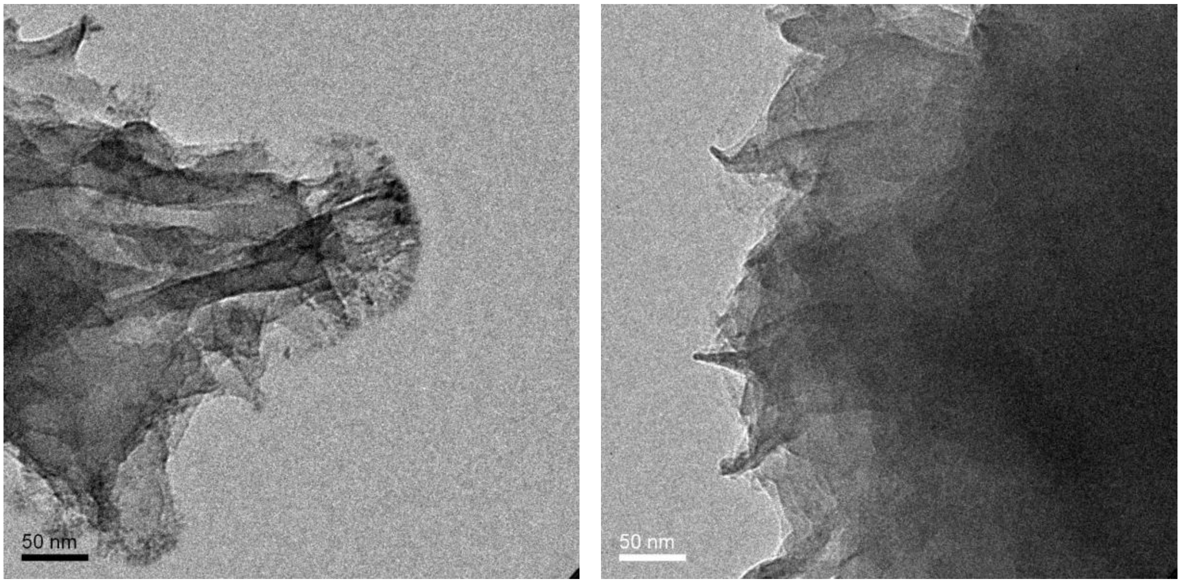

Supplementary Figure 22: TEM images of **N_0-COF**.

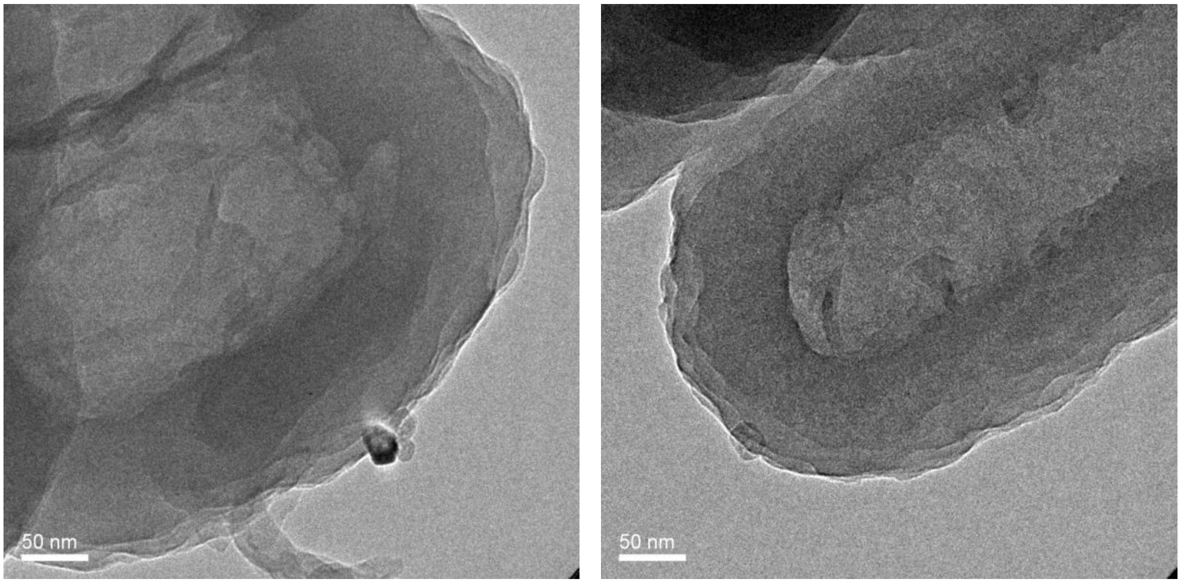

Supplementary Figure 23: TEM images of **N_1-COF**.

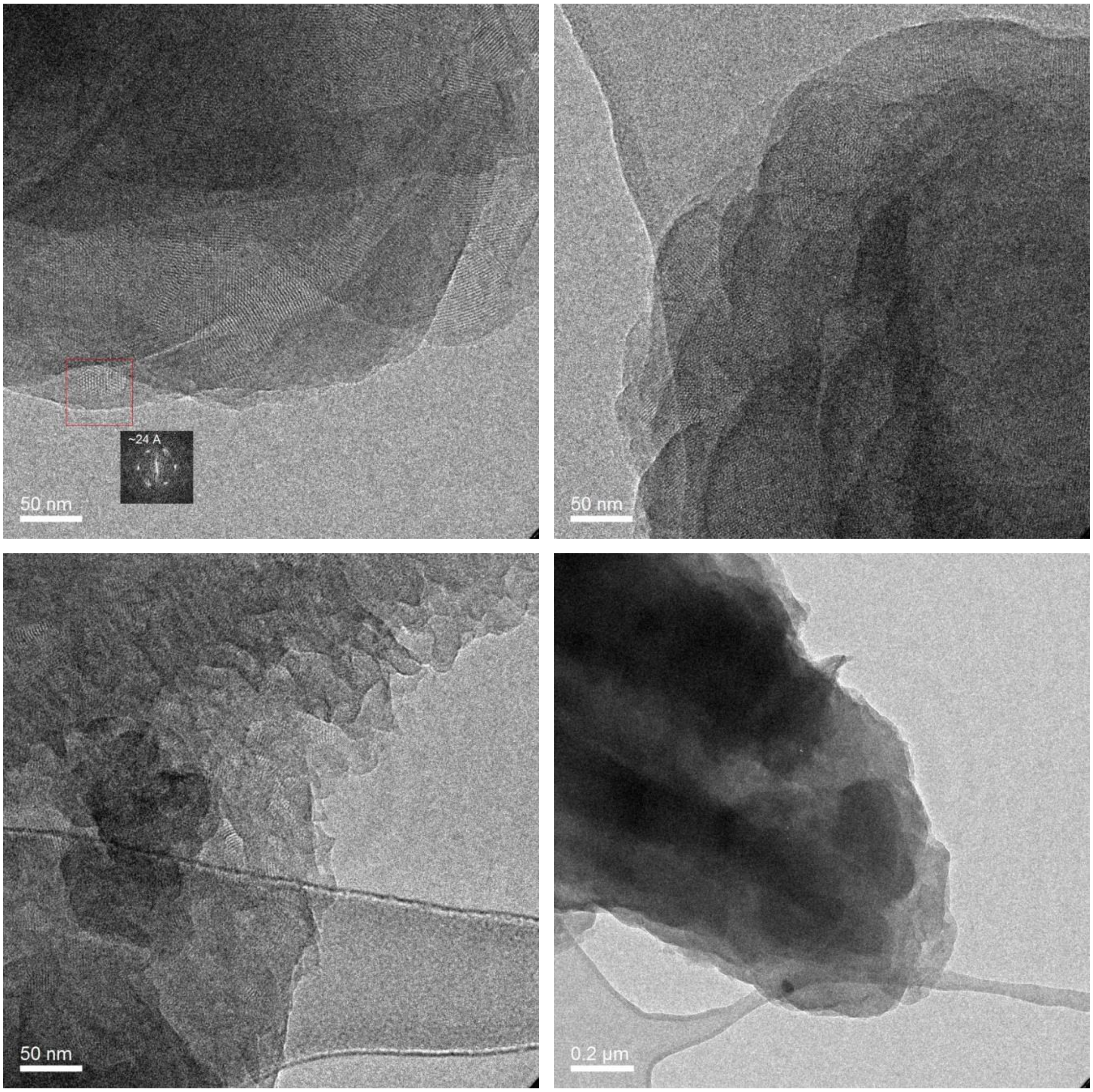

Supplementary Figure 24: TEM images of **N_2-COF**.

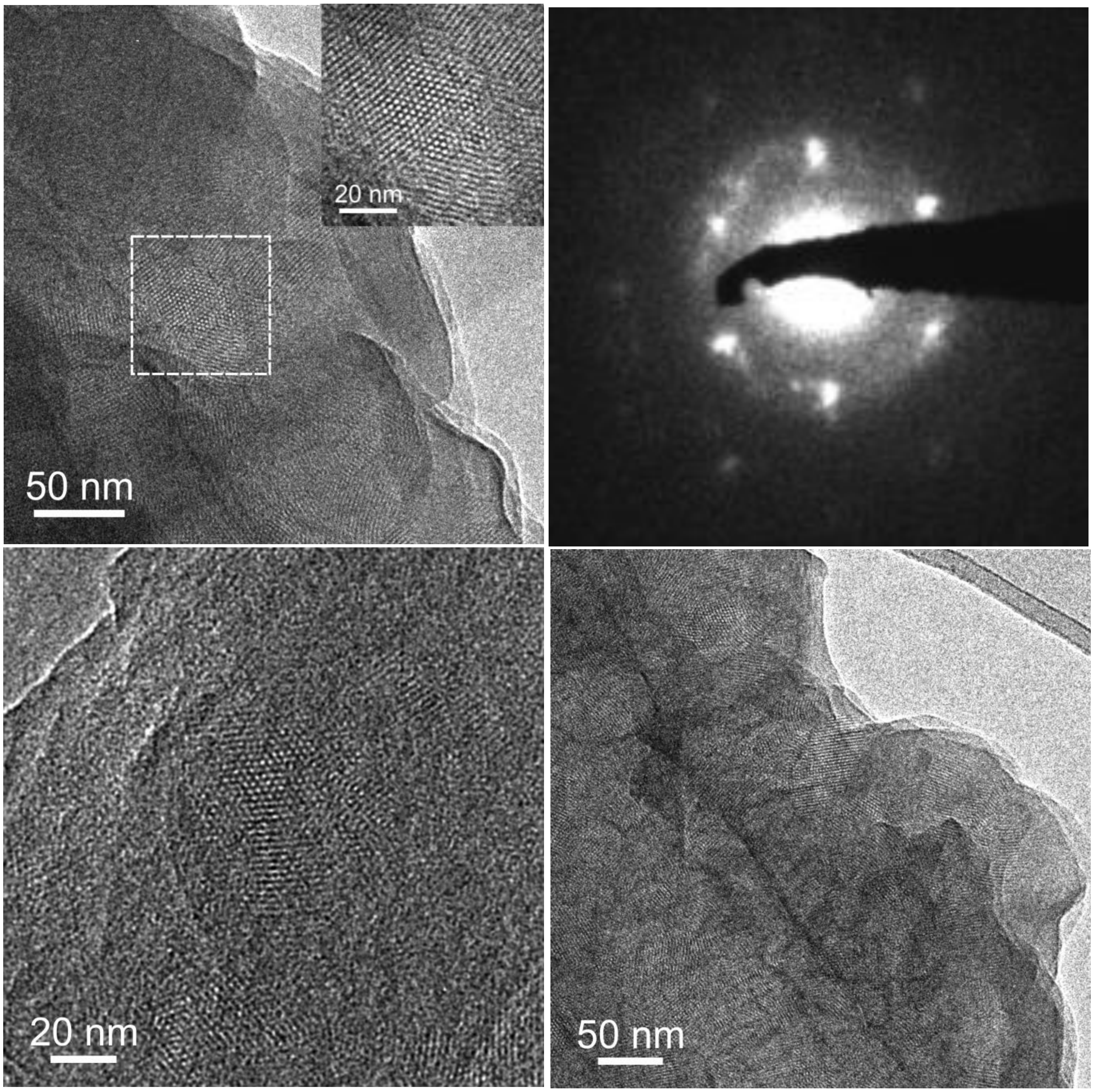

Supplementary Figure 25: TEM images of **N_3-COF**.

1.1. TGA in Argon

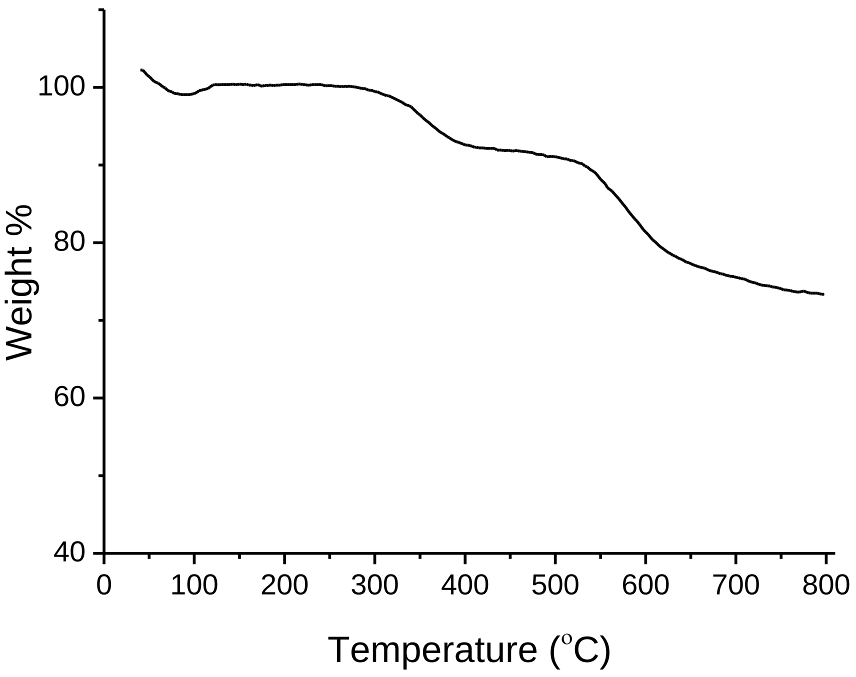

Supplementary Figure 26: TGA of **N_0-COF** in Argon.

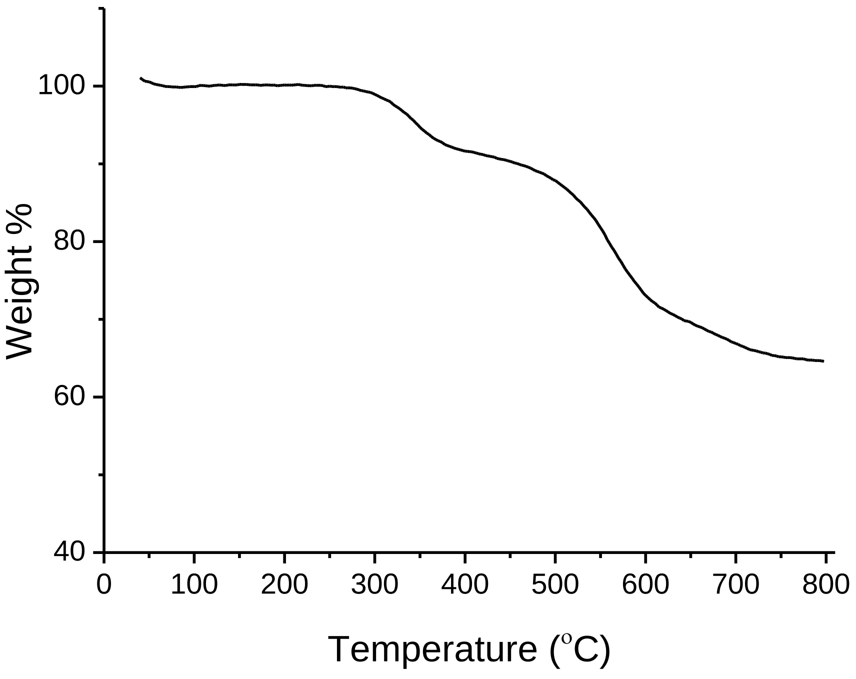

Supplementary Figure 27: TGA of **N_1-COF** in Argon.

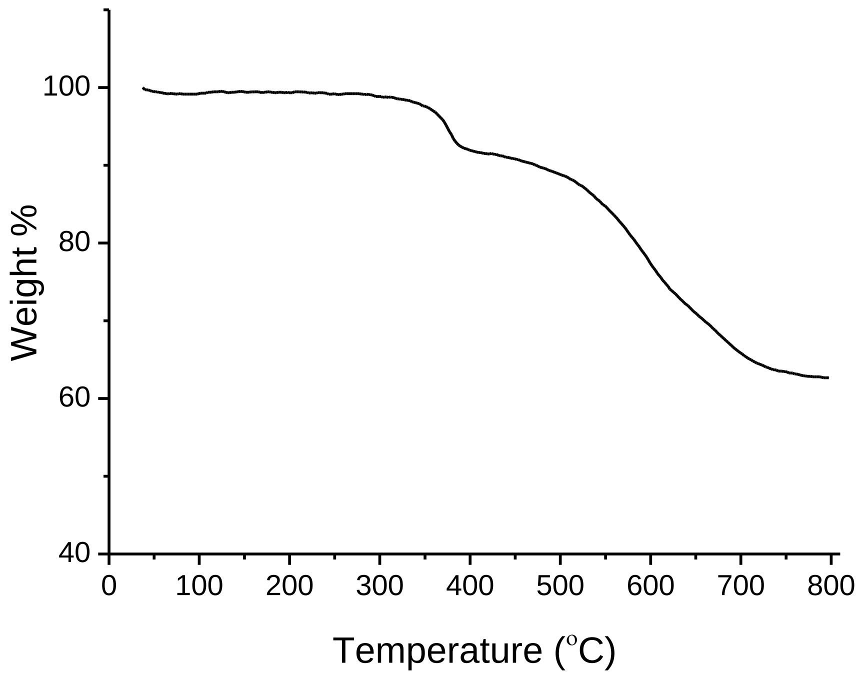

Supplementary Figure 28: TGA of **N_2-COF** in Argon.

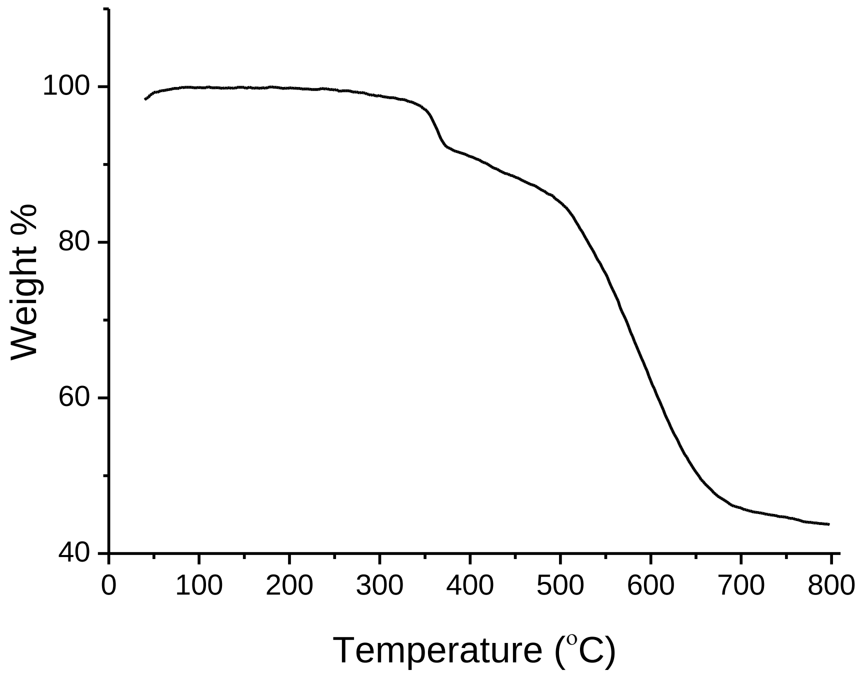

Supplementary Figure 29: TGA of **N_3-COF** in Argon.

1.2. TGA in Air

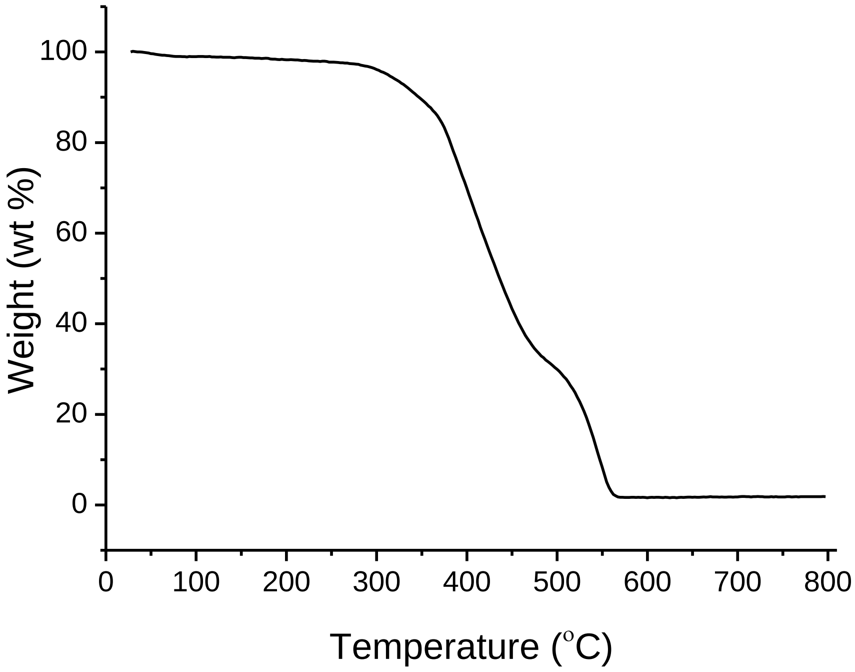

Supplementary Figure 30: TGA of $\mathbf{N_0}$**-COF** in Air.

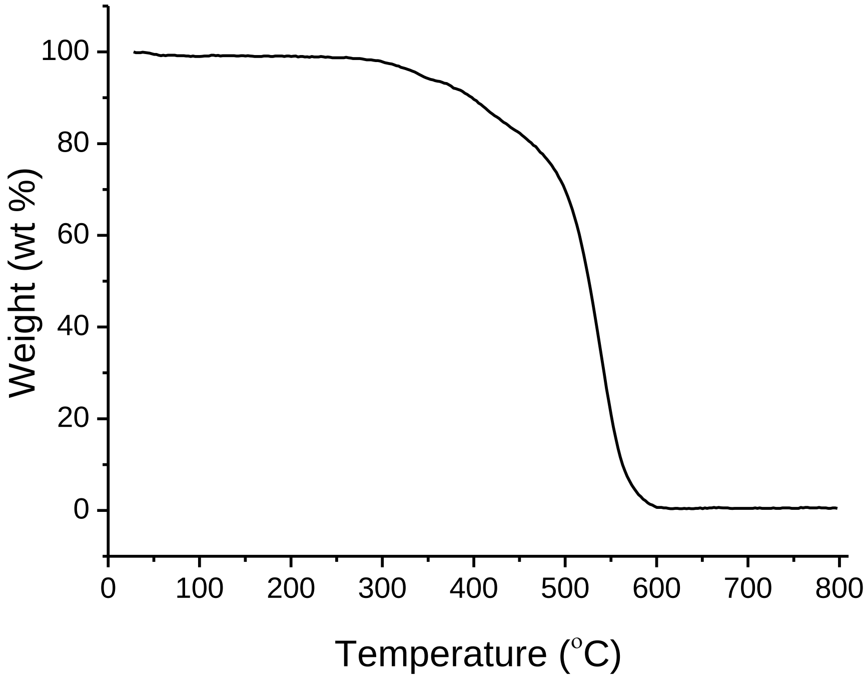

Supplementary Figure 31: TGA of $\mathbf{N_1}$**-COF** in Air.

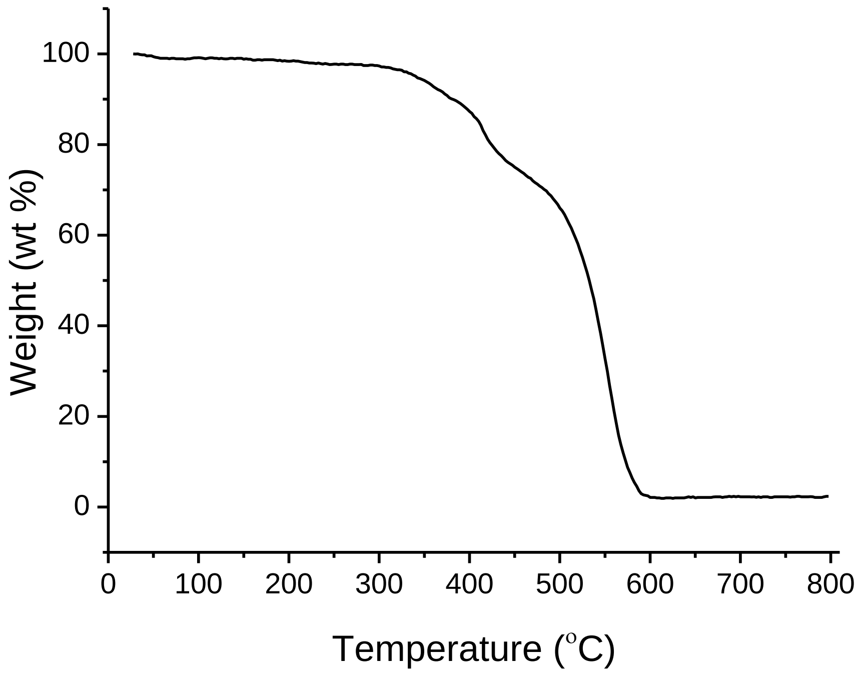

Supplementary Figure 32: TGA of $\mathbf{N_2}$**-COF** in Air.

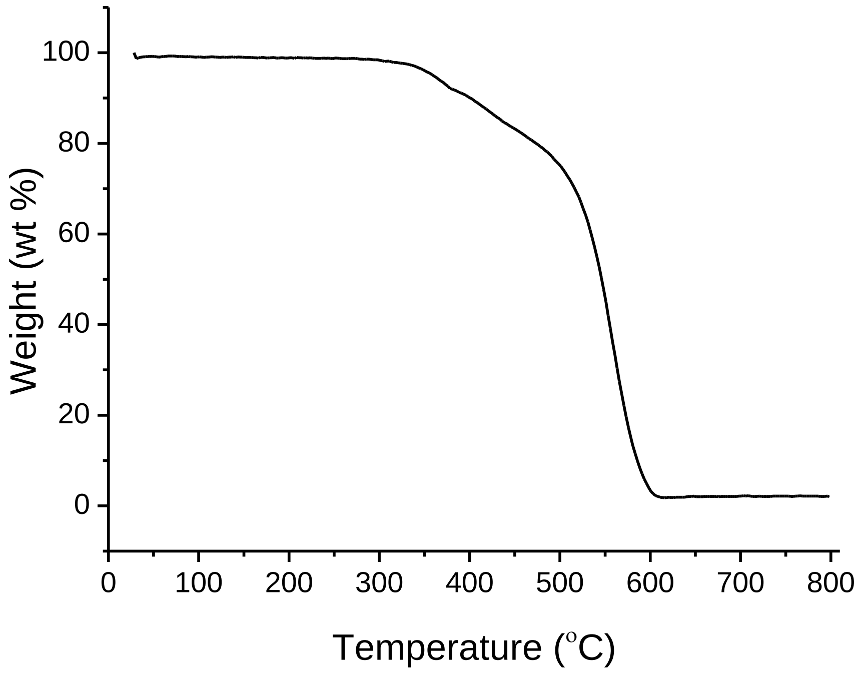

Supplementary Figure 33: TGA of $\mathbf{N_3}$**-COF** in Air.

1.3. Structure Simulation and PXRD

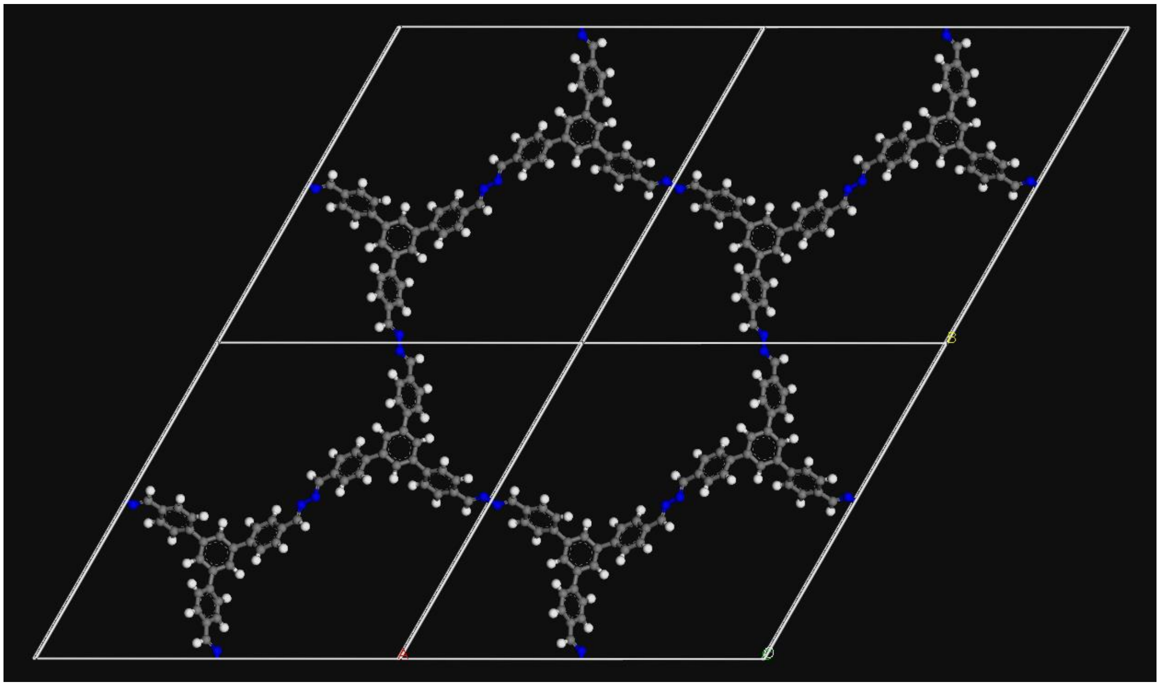

Supplementary Figure 34: View of the simulated structure of the **N_0-COF** along the *c* axis.

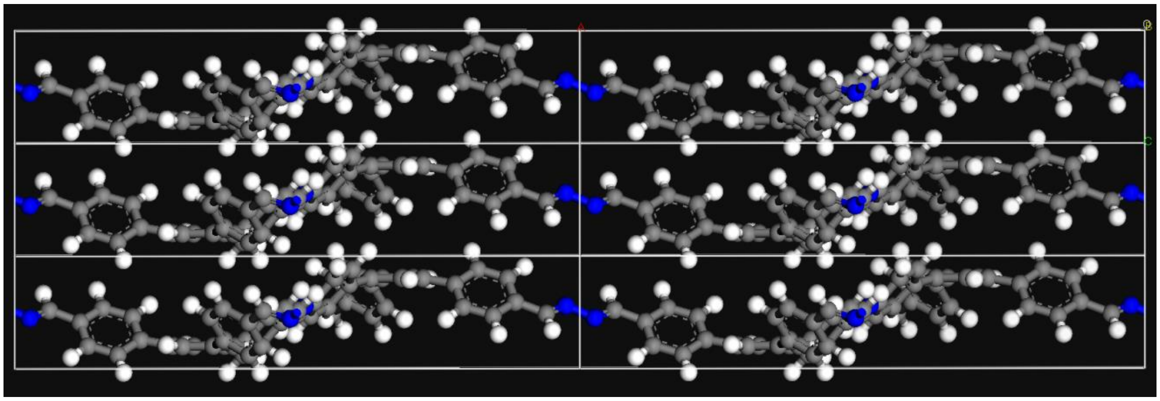

Supplementary Figure 35: View of the simulated structure of the **N_0-COF** along the *a* axis.

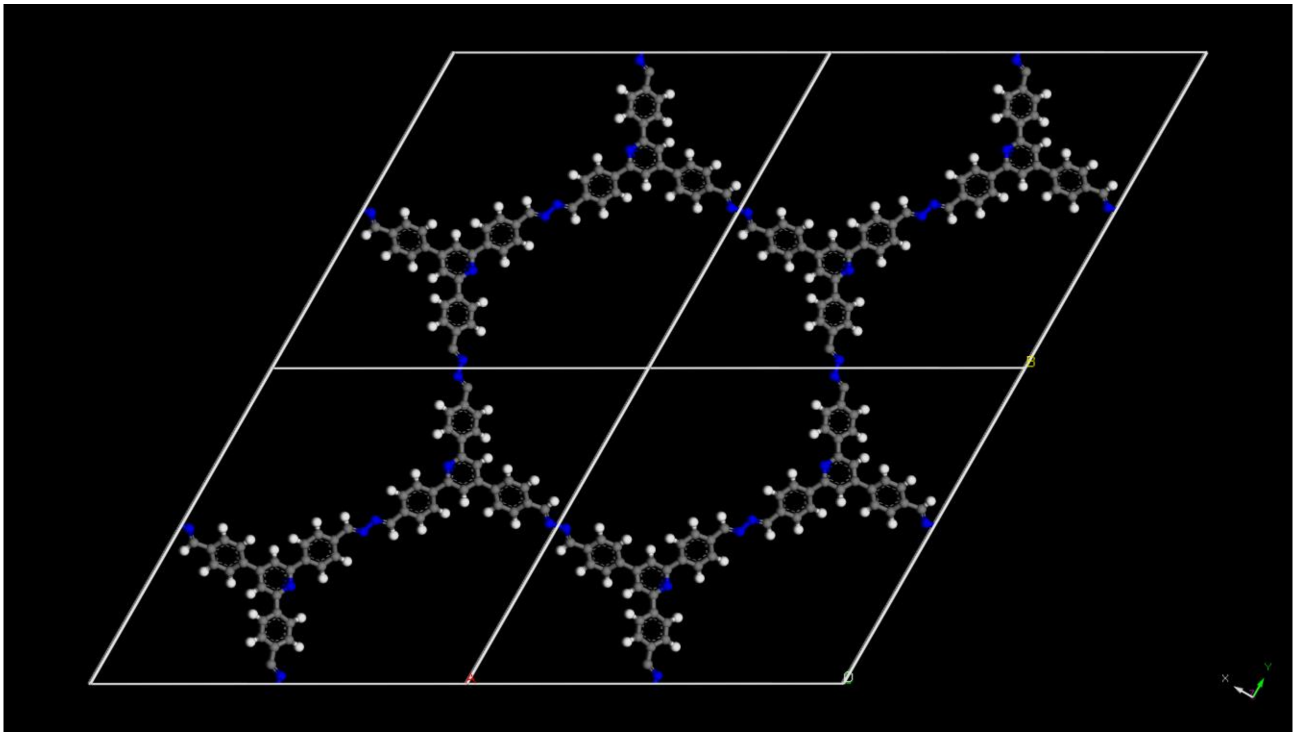

Supplementary Figure 36: View of the simulated structure of the **N_1-COF** along the *c* axis.

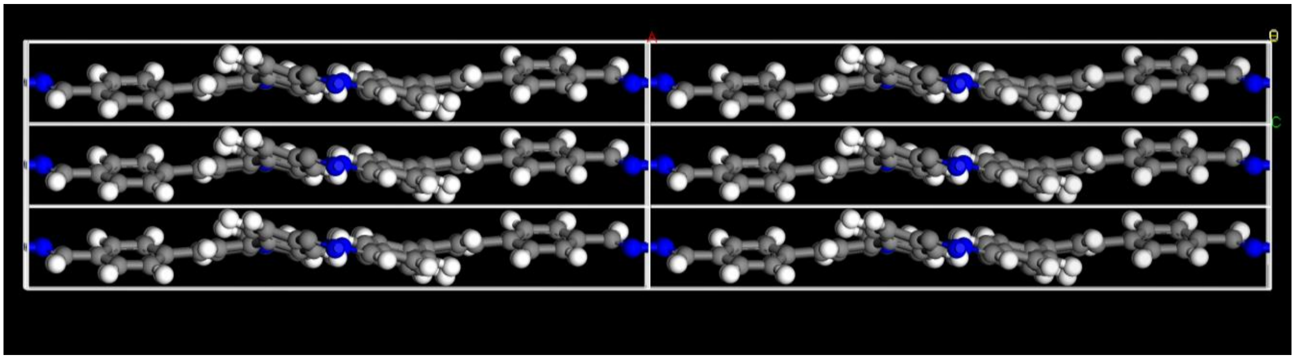

Supplementary Figure 37: View of the simulated structure of the **N_1-COF** along the *a* axis.

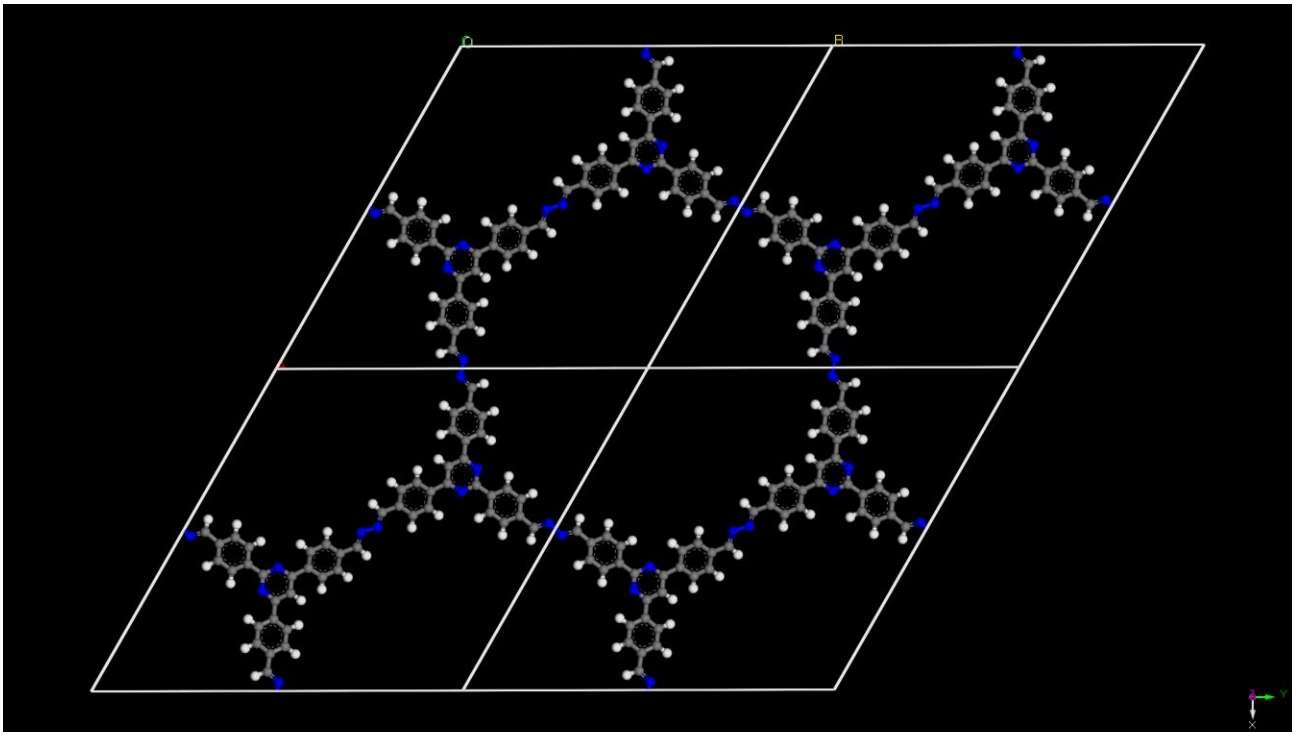

Supplementary Figure 38: View of the simulated structure of the $\mathbf{N_2}$**-COF** along the *c* axis.

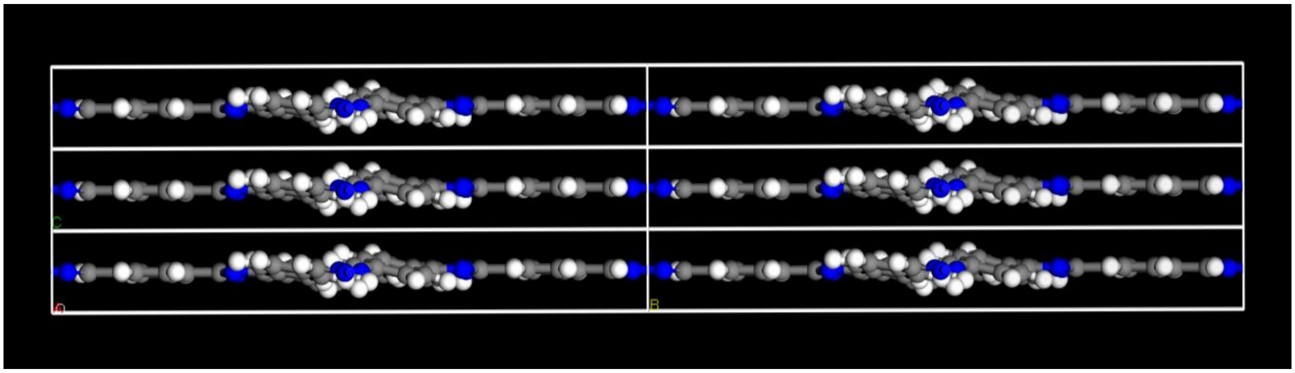

Supplementary Figure 39: View of the simulated structure of the $\mathbf{N_2}$**-COF** along the *a* axis.

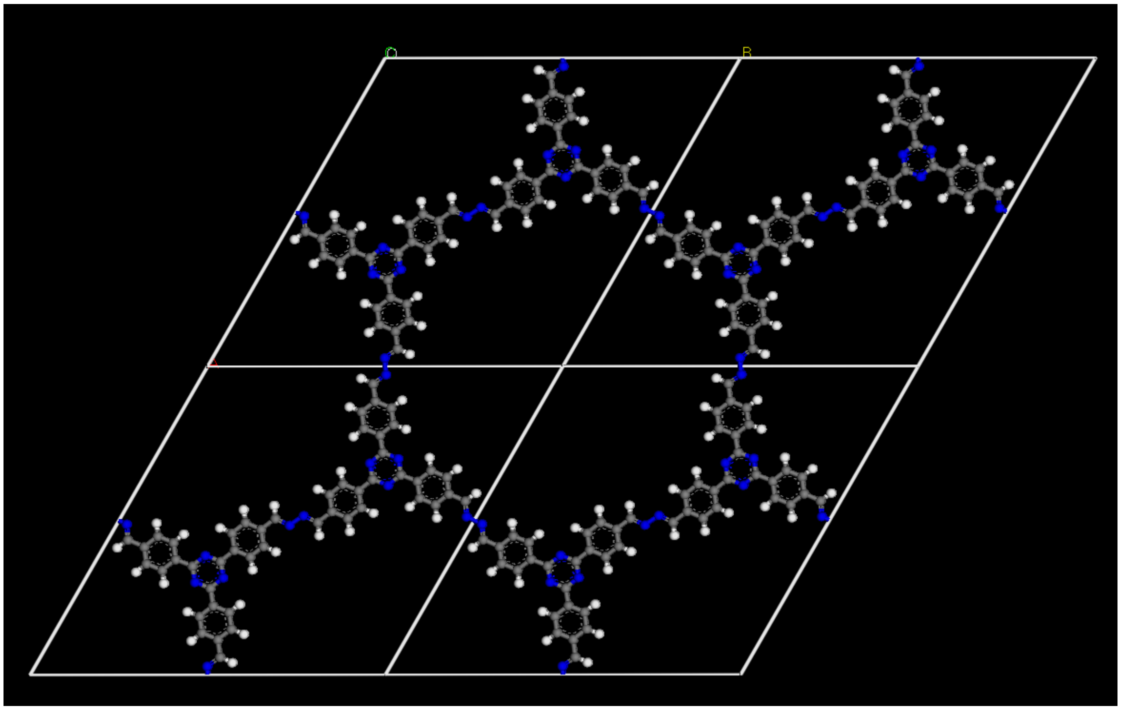

Supplementary Figure 40: View of the simulated structure of the **N_3-COF** along the *c* axis.

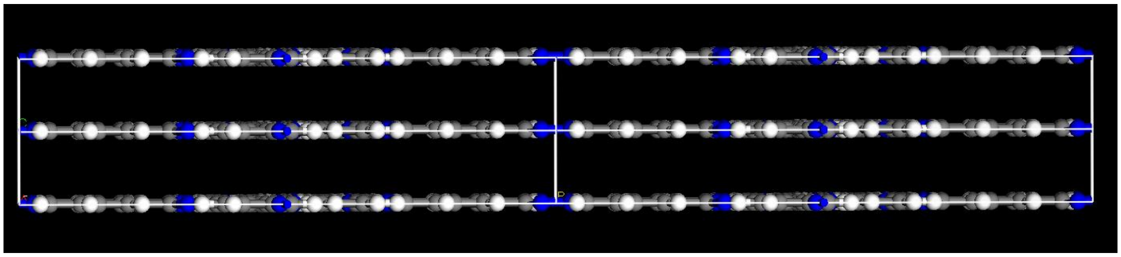

Supplementary Figure 41: View of the simulated structure of the **N_3-COF** along the *a* axis.

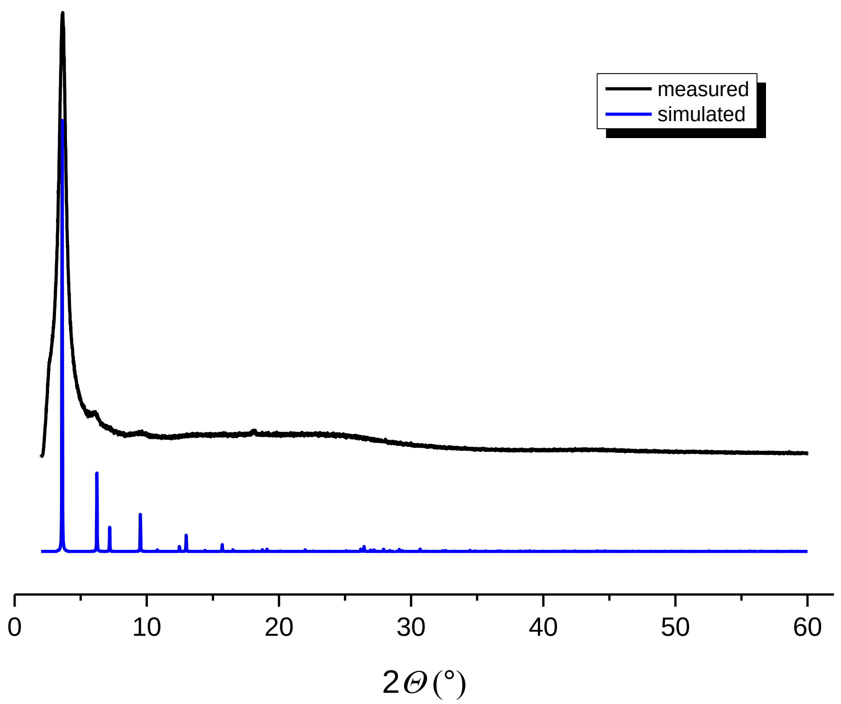

Supplementary Figure 42: Experimental powder X-ray diffraction pattern of **N_0-COF** (black), compared with the simulated PXRD pattern assuming $P\bar{3}$ symmetry (blue).

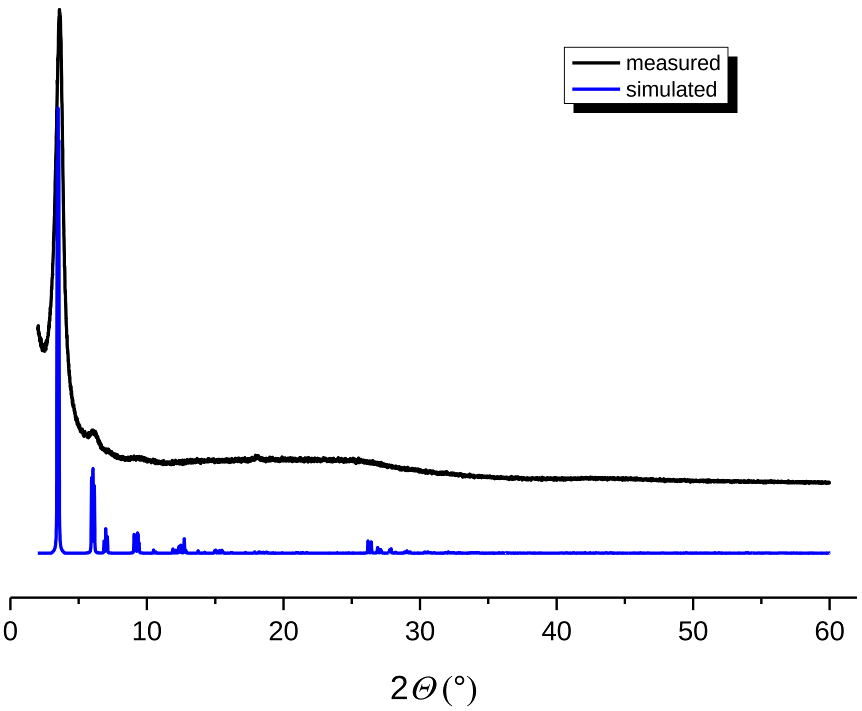

Supplementary Figure 43: Experimental powder X-ray diffraction pattern of **N_1-COF** (black), compared with the simulated PXRD pattern assuming $P\bar{1}$ symmetry (blue).

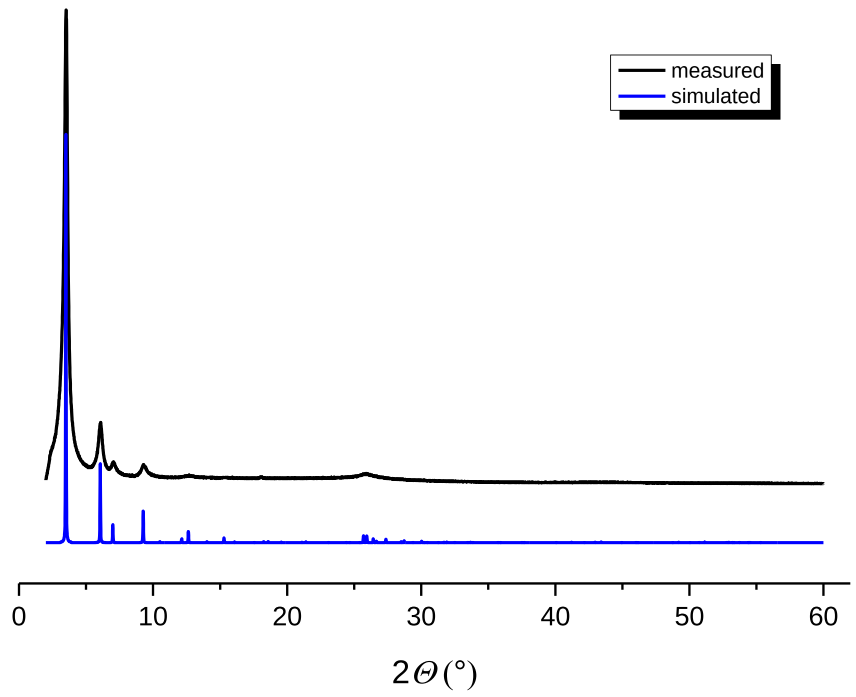

Supplementary Figure 44: Experimental powder X-ray diffraction pattern of **N_2-COF** (black), compared with the simulated PXRD pattern assuming $P\bar{1}$ symmetry (blue).

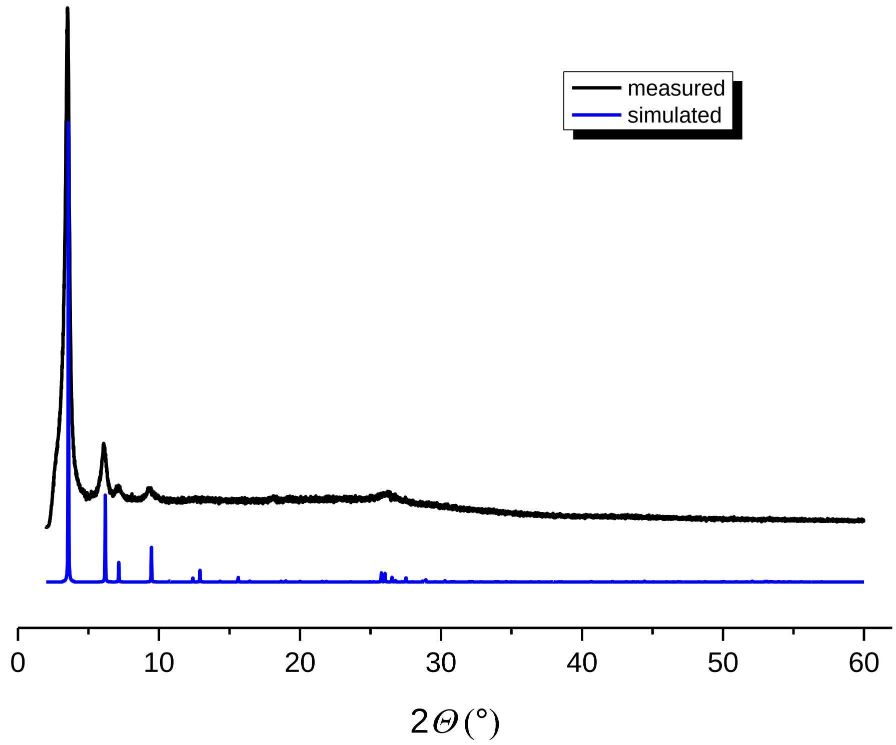

Supplementary Figure 45: Experimental powder X-ray diffraction pattern of **N_3-COF** (black), compared with the simulated PXRD pattern assuming $P6/m$ symmetry (blue).

1.4. Sorption

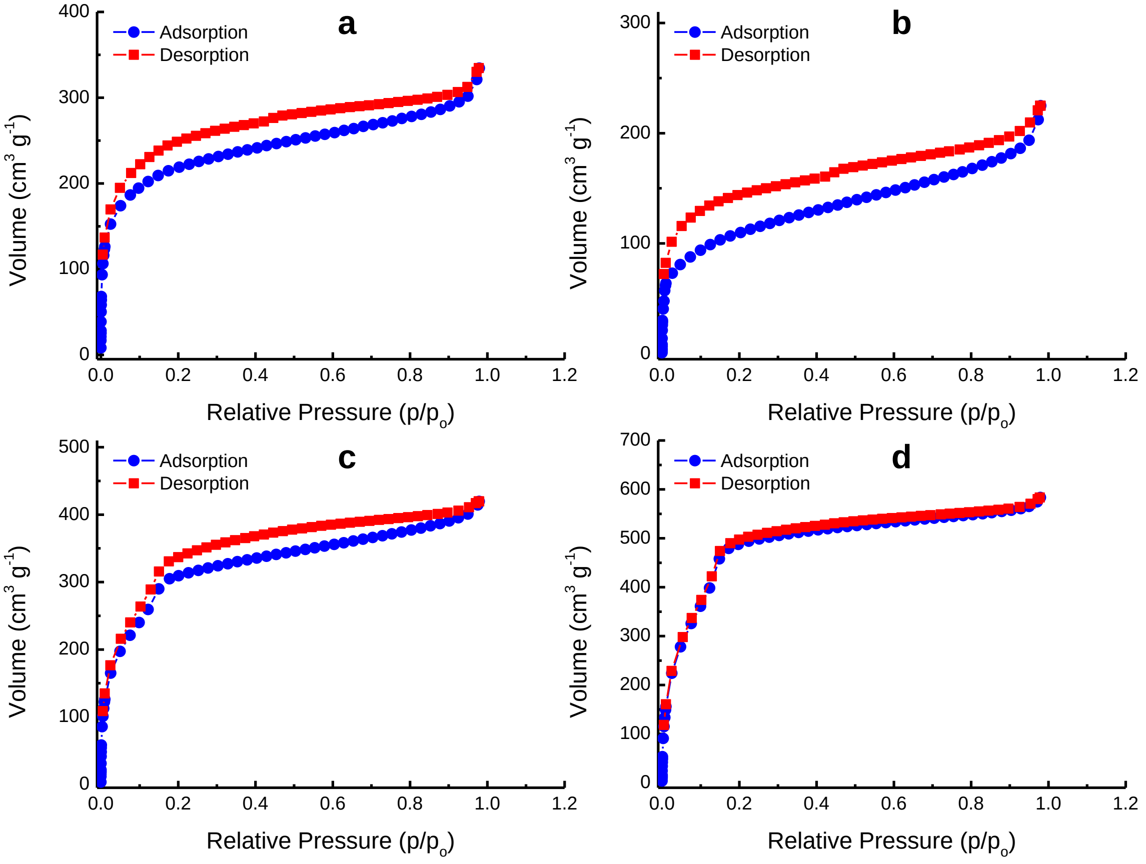

Supplementary Figure 46: Argon sorption isotherms of the N_x-COFs measured at 87 K. (a) **N_0-COF**, (b) **N_1-COF**, (c) **N_2-COF**, (d) **N_3-COF**.

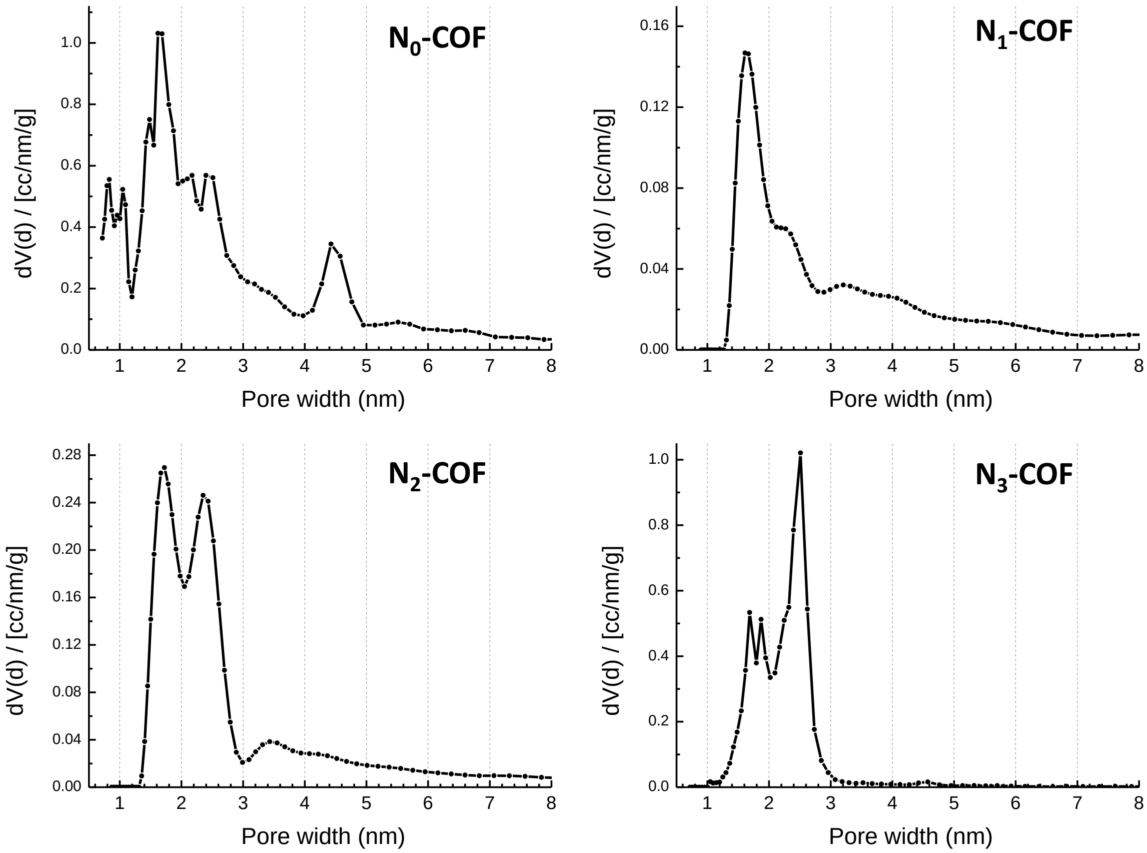

Supplementary Figure 47: Pore Size Distribution (PSD) of the COFs calculated by non-local density functional theory (NLDFT) using the "Ar-zeolite/silica cylindrical pores at 87 K" kernel accessible in the ASiQwin software.

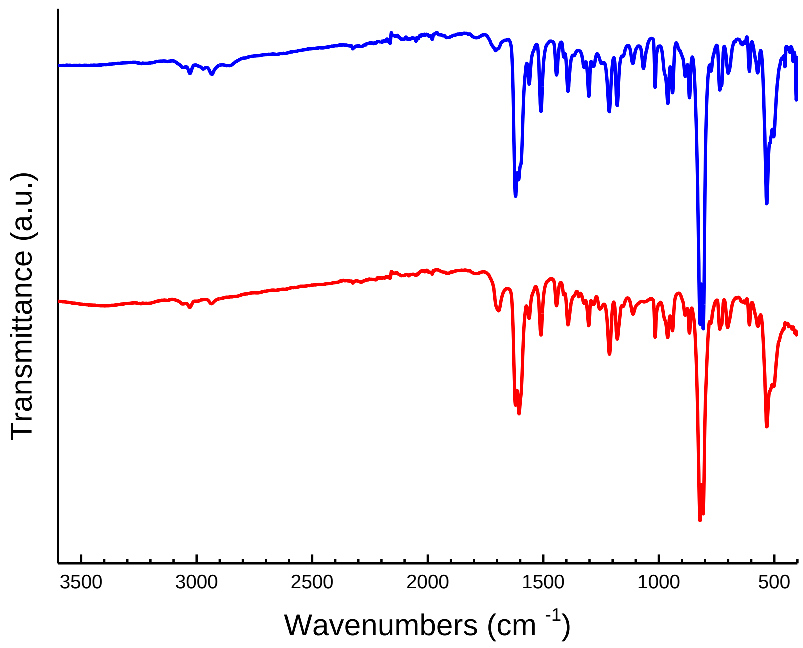

Supplementary Figure 48: FT-IR spectra of **N$_0$-COF** before (blue) and after (red) photocatalysis.

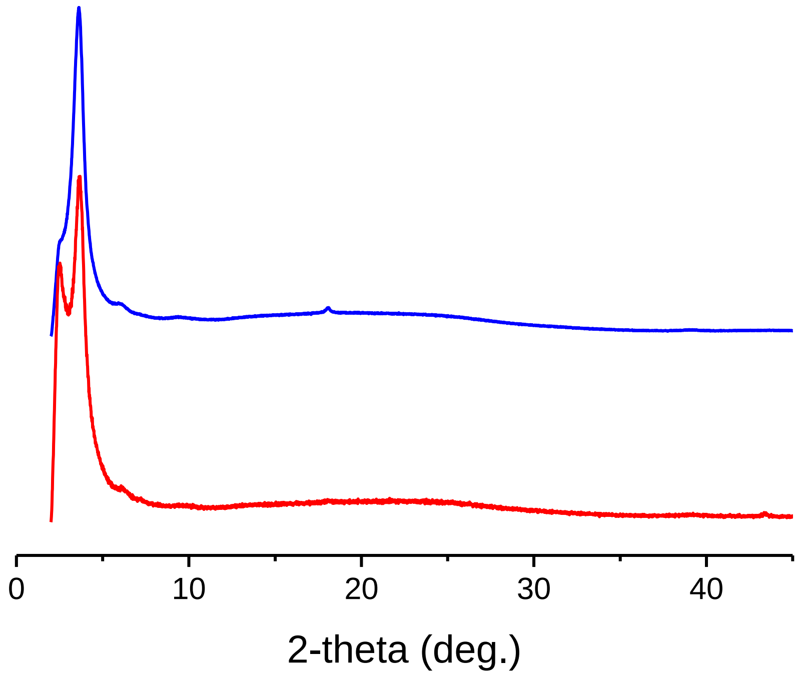

Supplementary Figure 49: PXRD of **N$_0$-COF** before (blue) and after (red) photocatalysis.

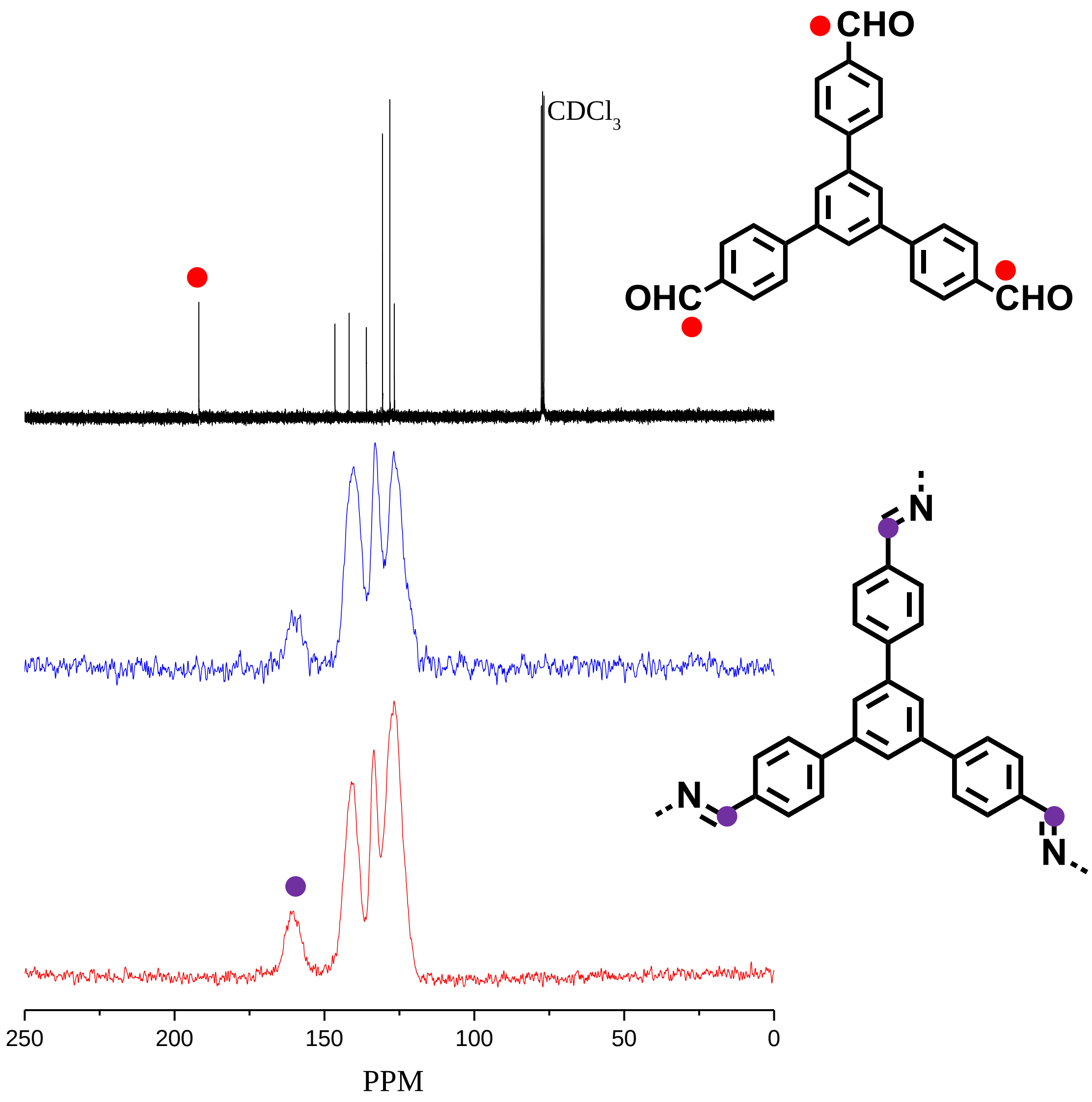

Supplementary Figure 50: Solution ^{13}C NMR of the **N_0-Ald** (black); ^{13}C ssNMR of **N_0-COF** before (blue) and after (red) photocatalysis. The peaks have been assigned to the carbons in the respective structures.

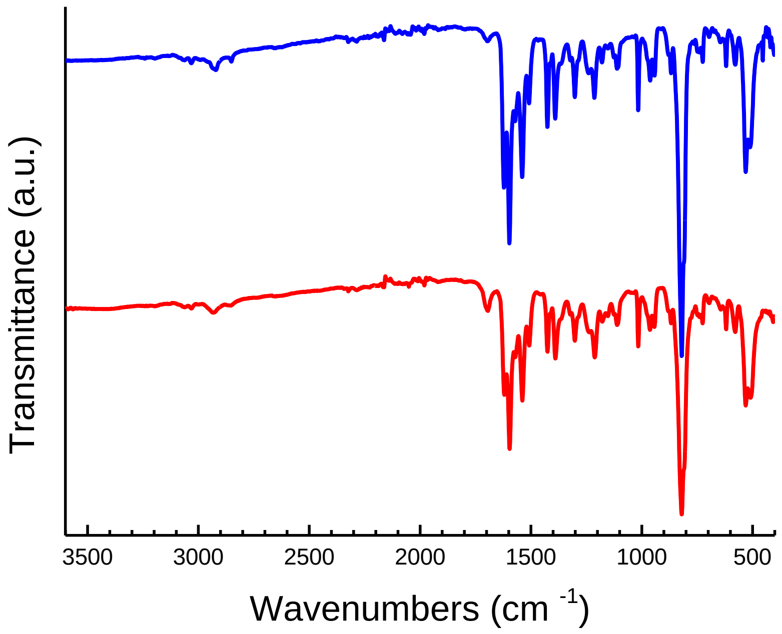

Supplementary Figure 51: FT-IR spectra of **N_1-COF** before (blue) and after (red) photocatalysis.

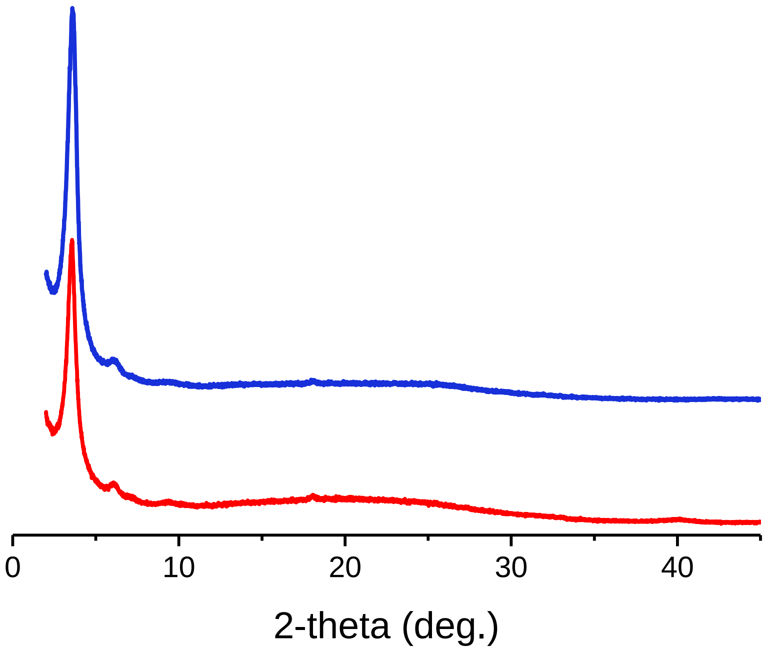

Supplementary Figure 52: PXRD **N_1-COF** before (blue) and after (red) photocatalysis.

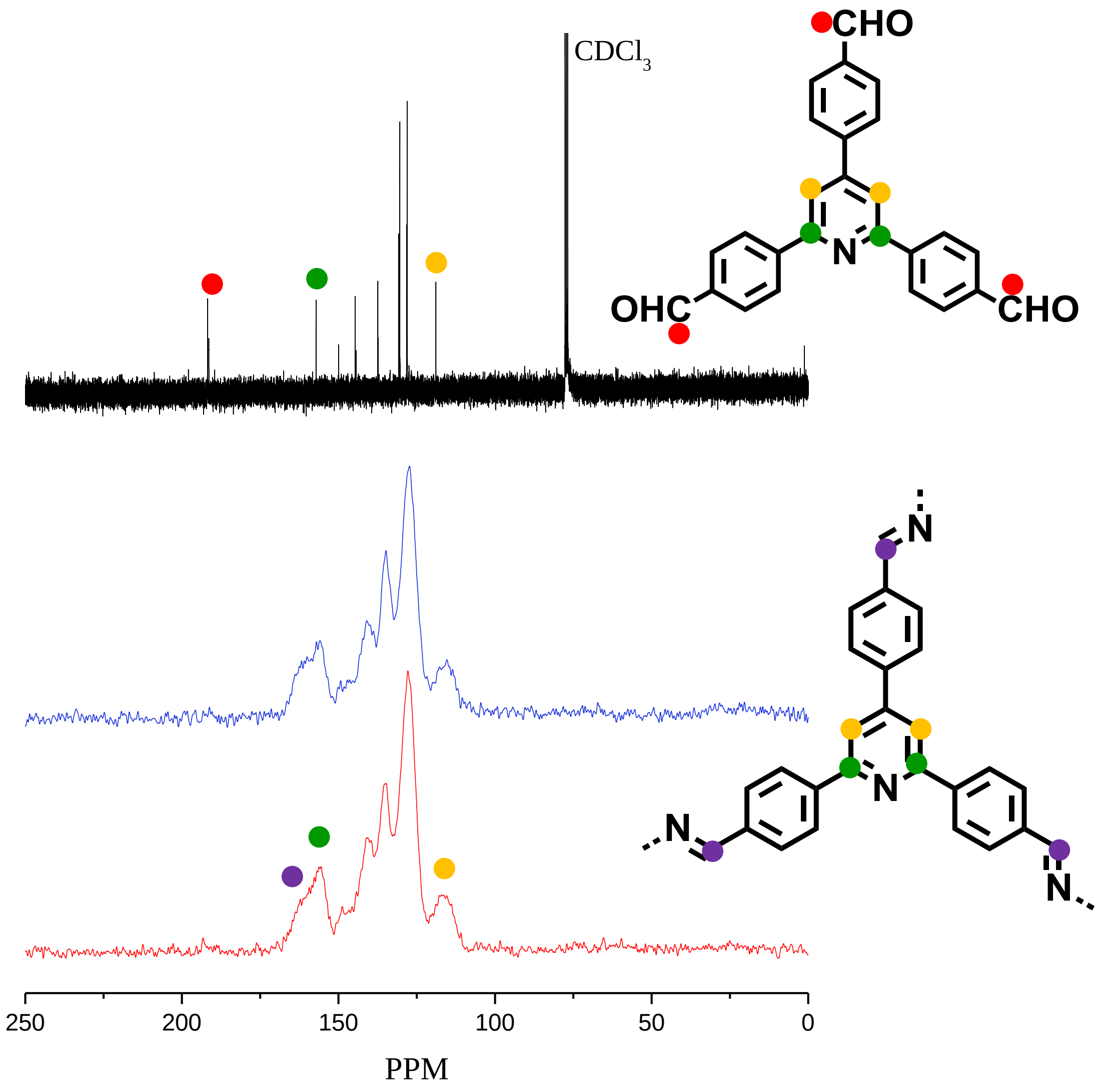

Supplementary Figure 53: Solution ^{13}C NMR of the **N_1-Ald** (black); ^{13}C ssNMR of **N_1-COF** before (blue) and after (red) photocatalysis. The peaks have been assigned to the carbons in the respective structures.

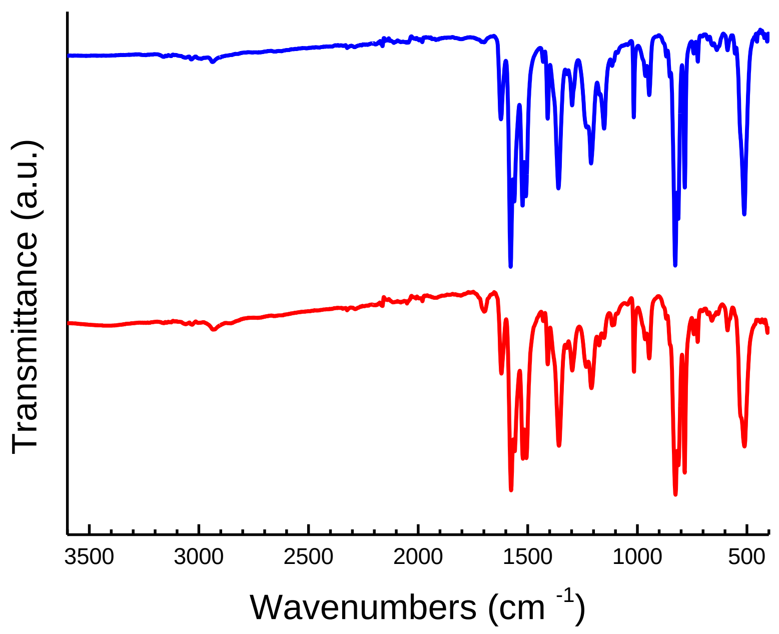

Supplementary Figure 54: FT-IR spectra of **N_2-COF** before (blue) and after (red) photocatalysis.

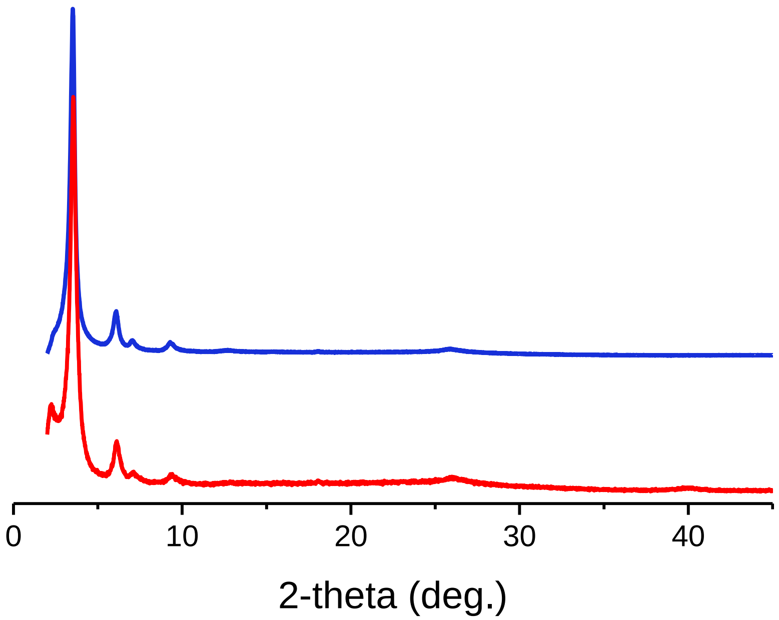

Supplementary Figure 55: PXRD **N_2-COF** before (blue) and after (red) photocatalysis.

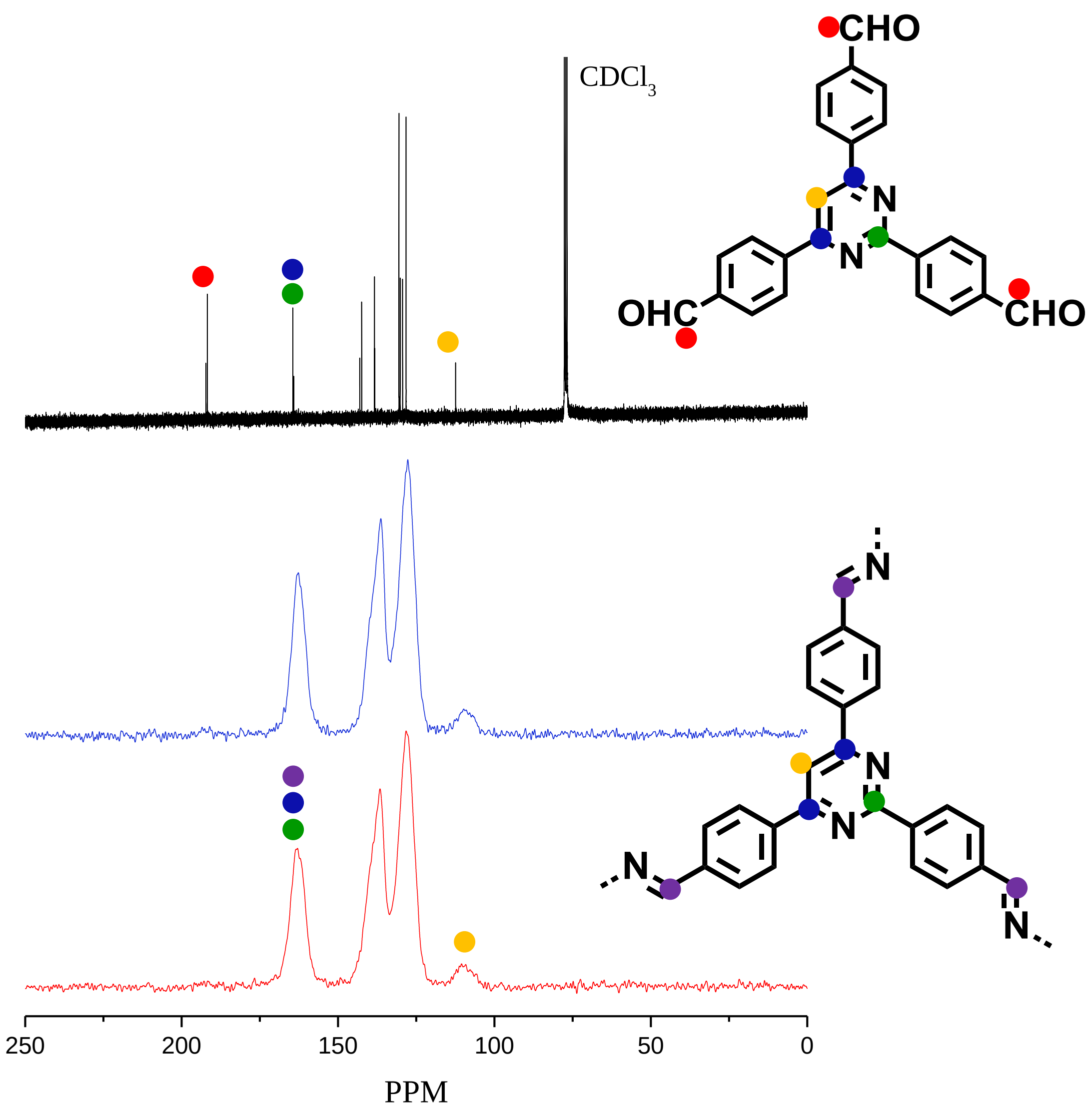

Supplementary Figure 56: Solution ^{13}C NMR of the **N_2-Ald** (black); ^{13}C ssNMR of **N_2-COF** before (blue) and after (red) photocatalysis. The peaks have been assigned to the carbons in the respective structures.

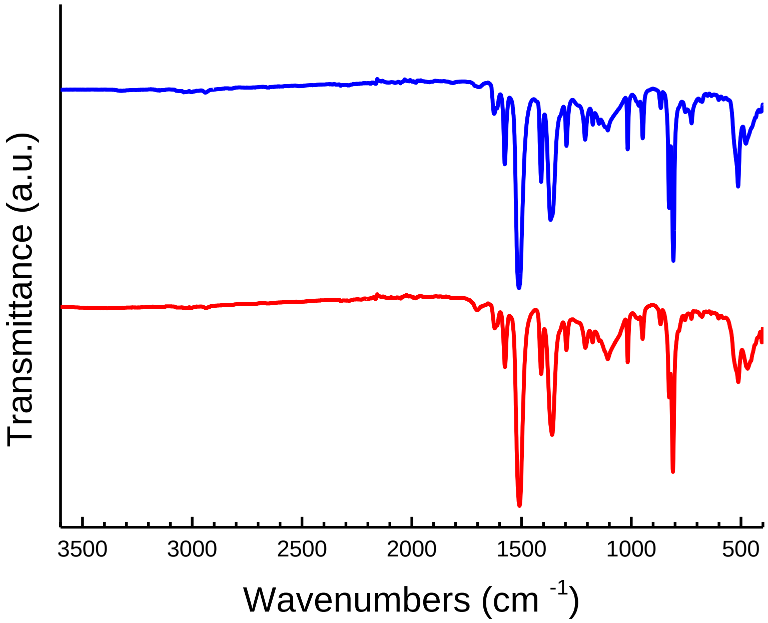

Supplementary Figure 57: FT-IR spectra of **N_3-COF** before (blue) and after (red) photocatalysis.

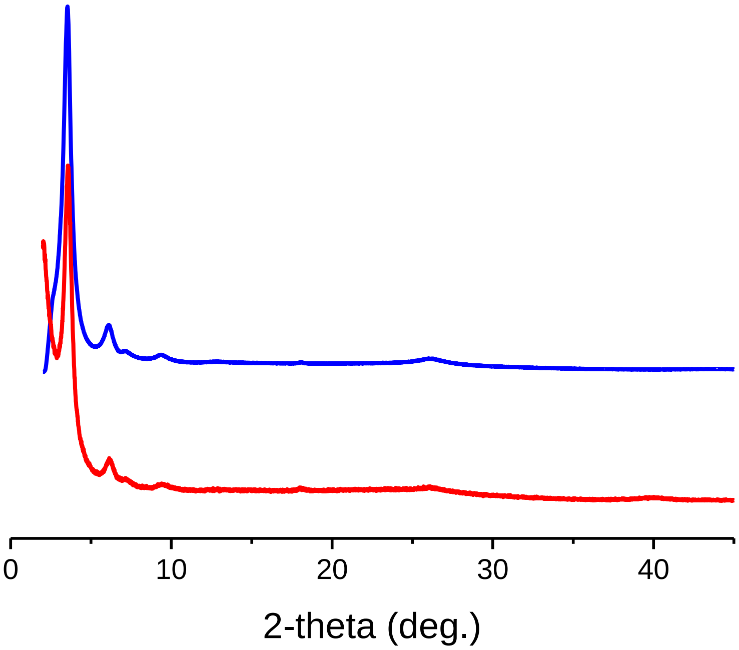

Supplementary Figure 58: PXRD of **N_3-COF** before (blue) and after (red) photocatalysis.

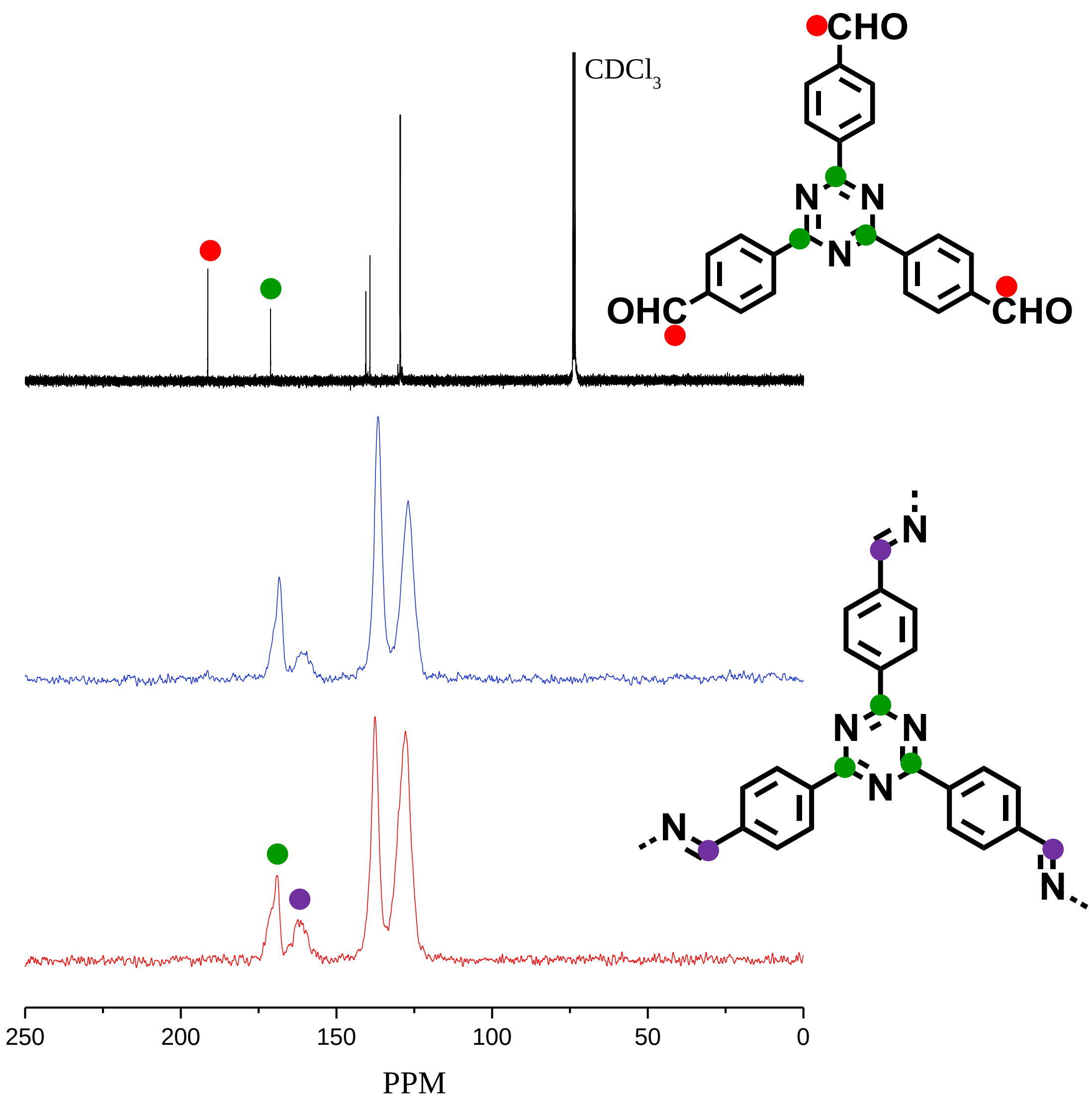

Supplementary Figure 59: Solution ^{13}C NMR of the **N_3-Ald** (black); ^{13}C ssNMR of **N_3-COF** before (blue) and after (red) photocatalysis. The peaks have been assigned to the carbons in the respective structures.

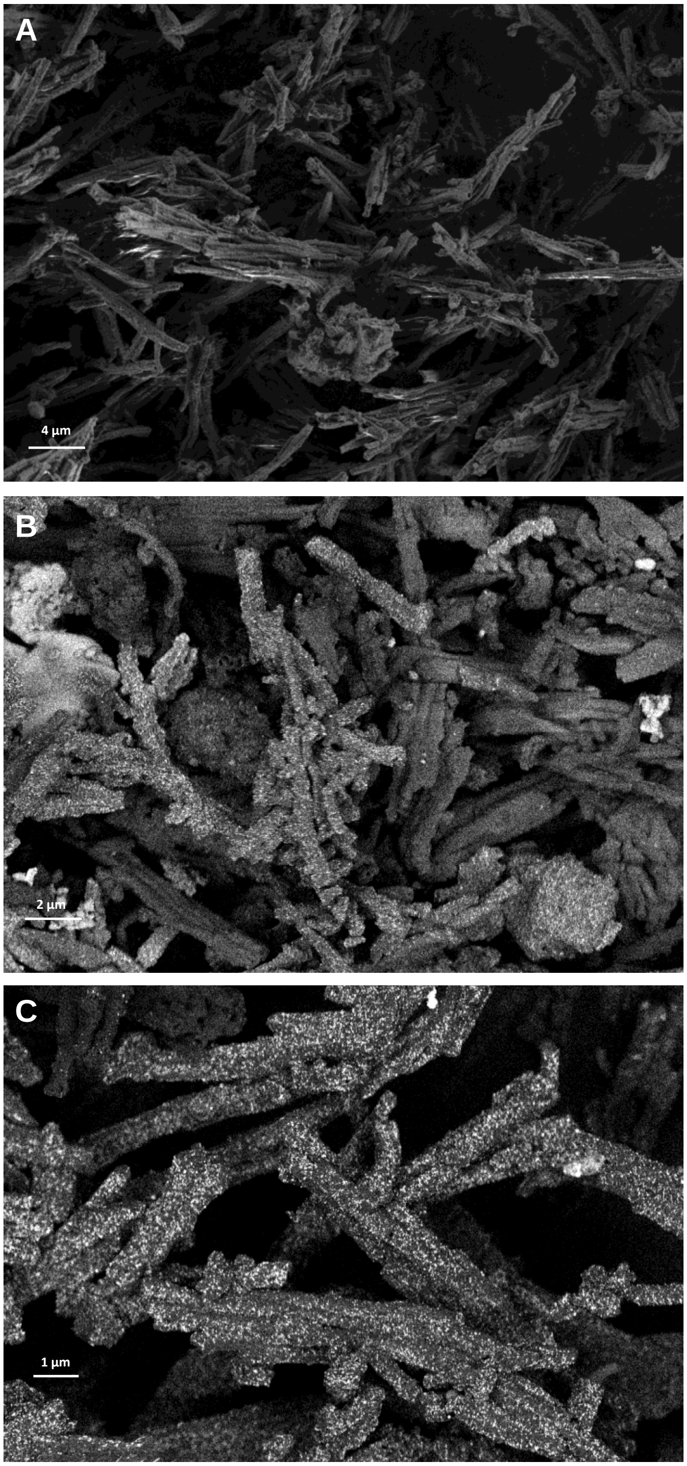

Supplementary Figure 60: SEM images of **N_3-COF** after photocatalysis: A. with secondary electron detector; B and C with backscatter detector to provide elemental contrast. The light spots are the Pt nanoparticles, which stay well-dispersed across the sample.

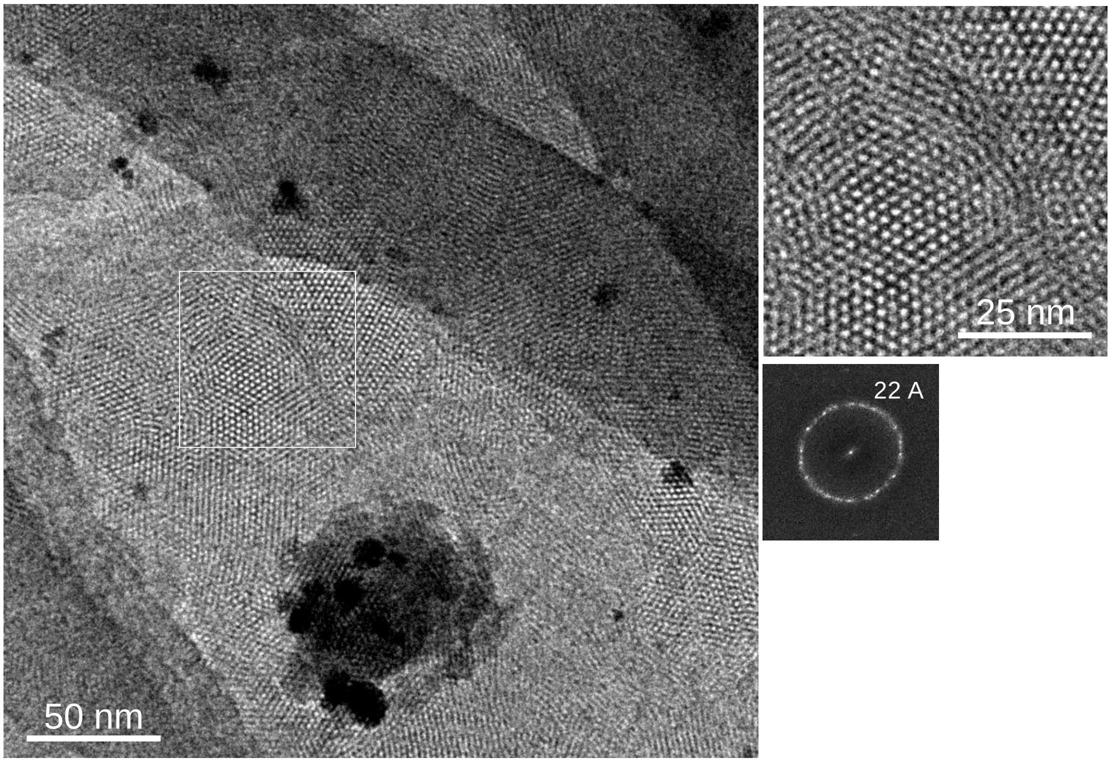

Supplementary Figure 61: TEM images of **N_3-COF** after photocatalysis. Bright field images and selected area diffraction pattern were recorded with a CMOS camera (TemCam-F216, TVIPS).

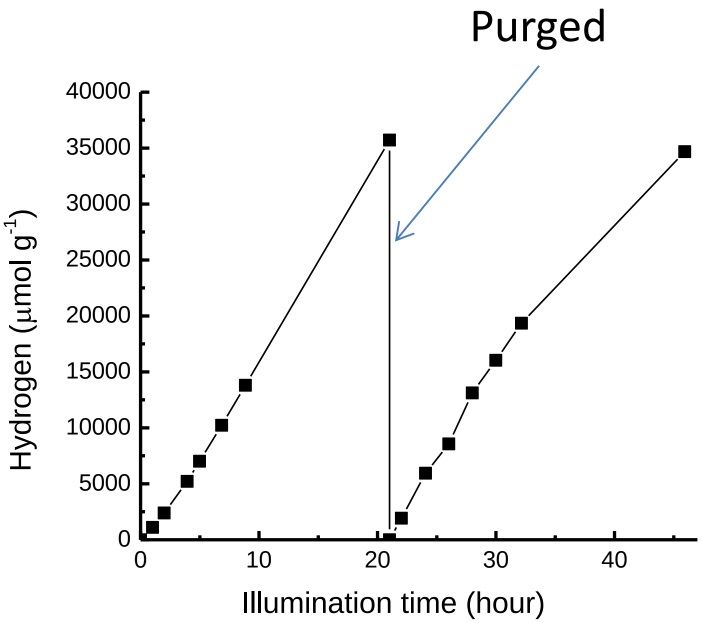

Supplementary Figure 62: Long term photocatalysis study of **N_3-COF** using triethanolamine as sacrificial electron donor.

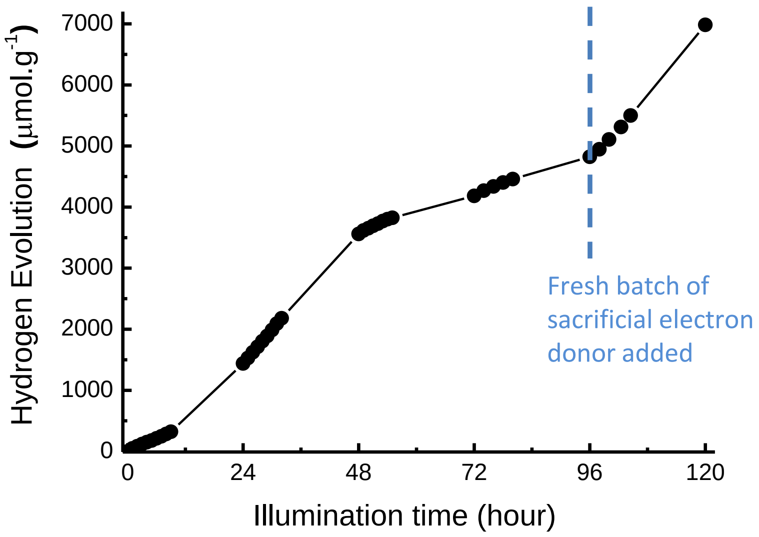

Supplementary Figure 63: Long term photocatalysis study of **N_3-COF** using ascorbic acid as sacrificial electron donor.

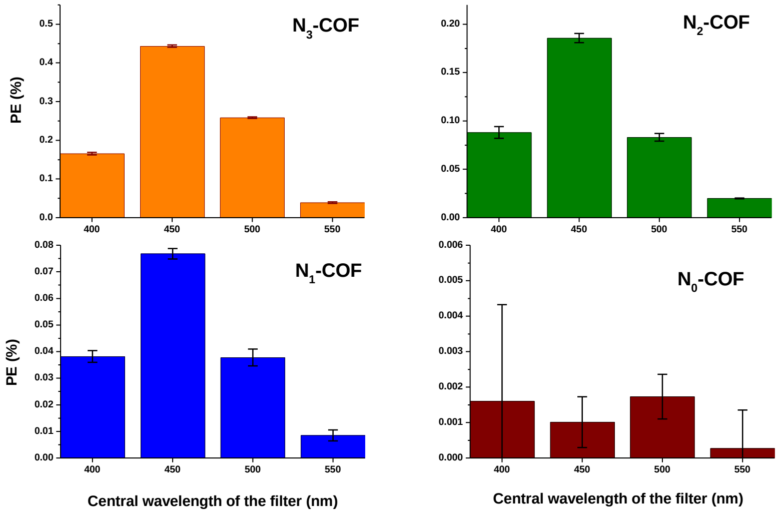

Supplementary Figure 64: Comparison of the photonic efficiency of N_x-COFs using different band-pass filters. The error bars represent the error of the gas chromatograph in integrating the amount of hydrogen evolved. The data for N_0-COF is of limited use due to its low photonic efficiency and, hence, large errors.

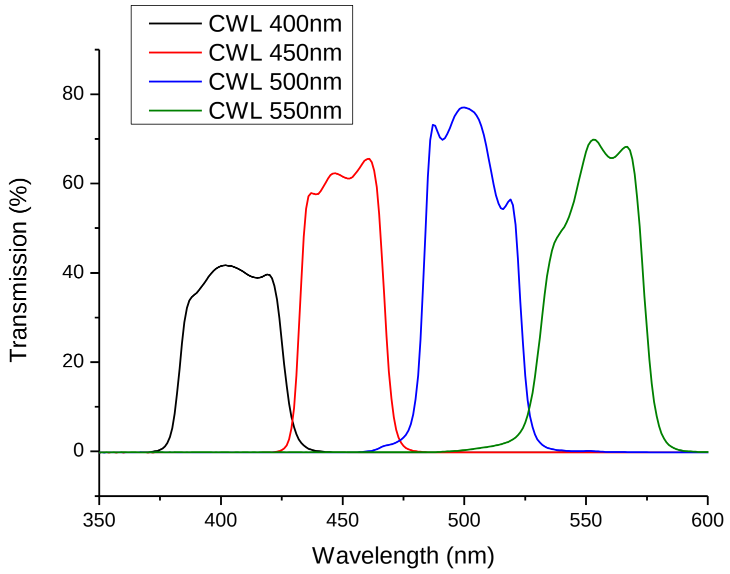

Supplementary Figure 65: Transmission spectra of the band-pass filters used for photonic efficiency calculations.

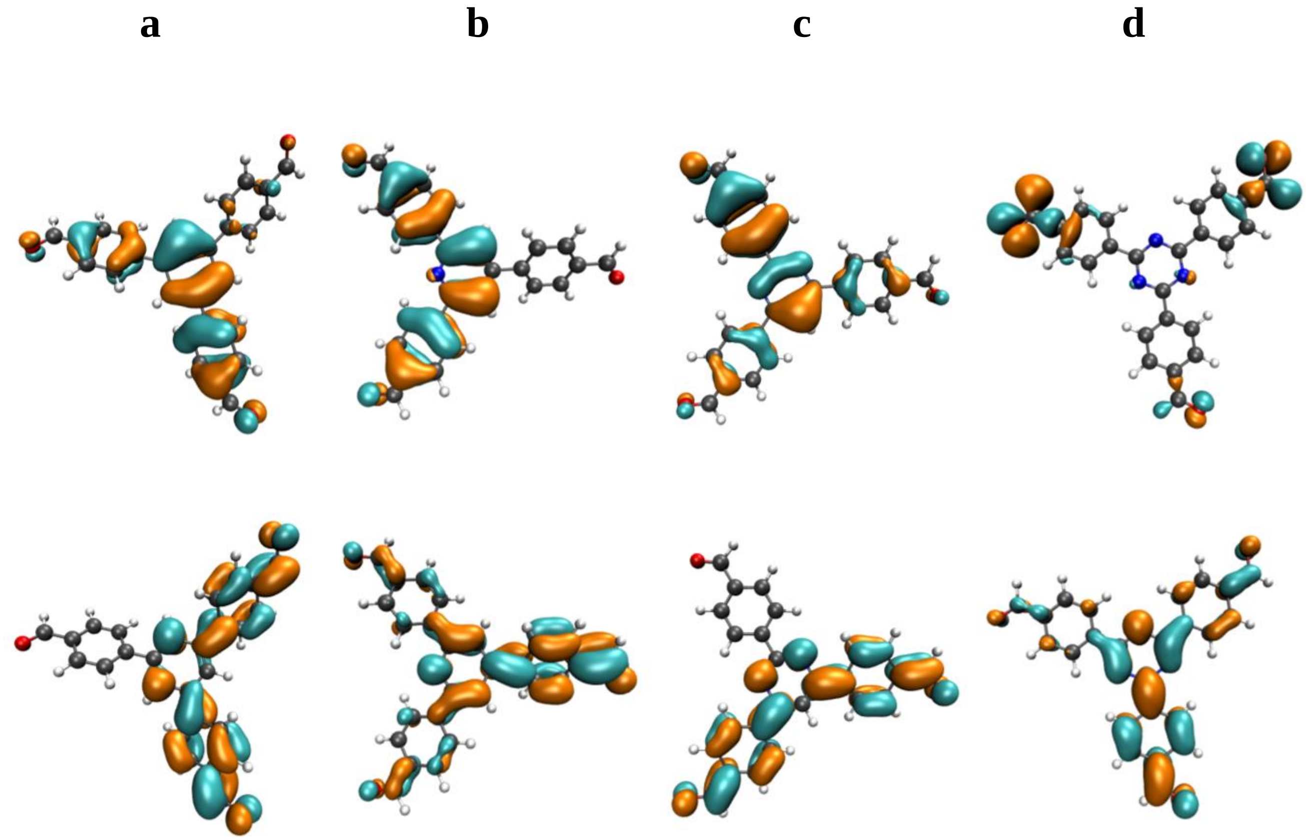

Supplementary Figure 66: HOMO and LUMO obtained at the PBE0-D3/def2-SVP level of theory for N_x-Aldehydes. (a) **N_0-Ald**, (b) **N_1- Ald**, (c) **N_2- Ald**, (d) **N_3- Ald**.

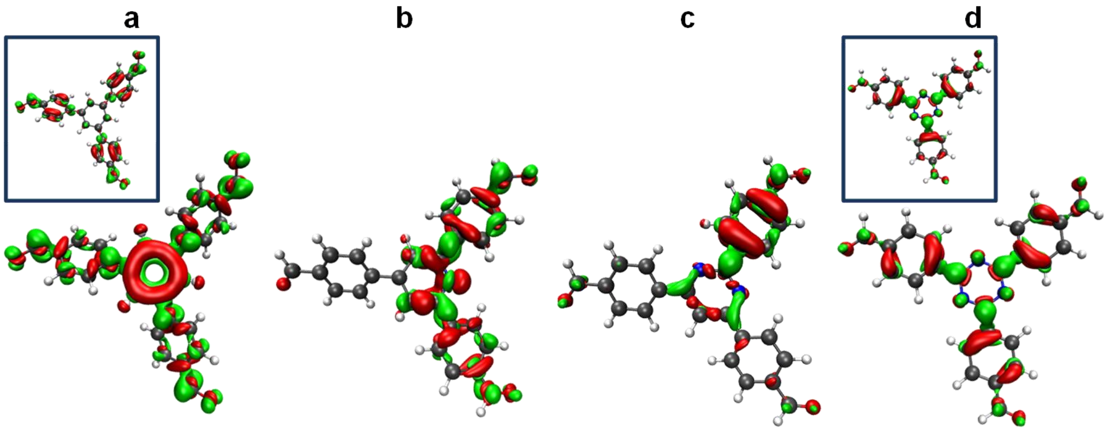

Supplementary Figure 67: Difference densities of vertical excitations with highest oscillator strength obtained at the time-dependent (TD)-PBE0/Def2-SVP level of theory for **Nx-Alds**. (a) **N_0-Ald**, (b) **N_1-Ald**, (c) **N_2-Ald**, (d) **N_3-Ald**. The red isosurfaces depict regions with lower electron density in the excited state whereas green isosurfaces represent higher electron density in the excited state, both in comparison to the electron density of the ground state. Inset shows the degenerate energy state.

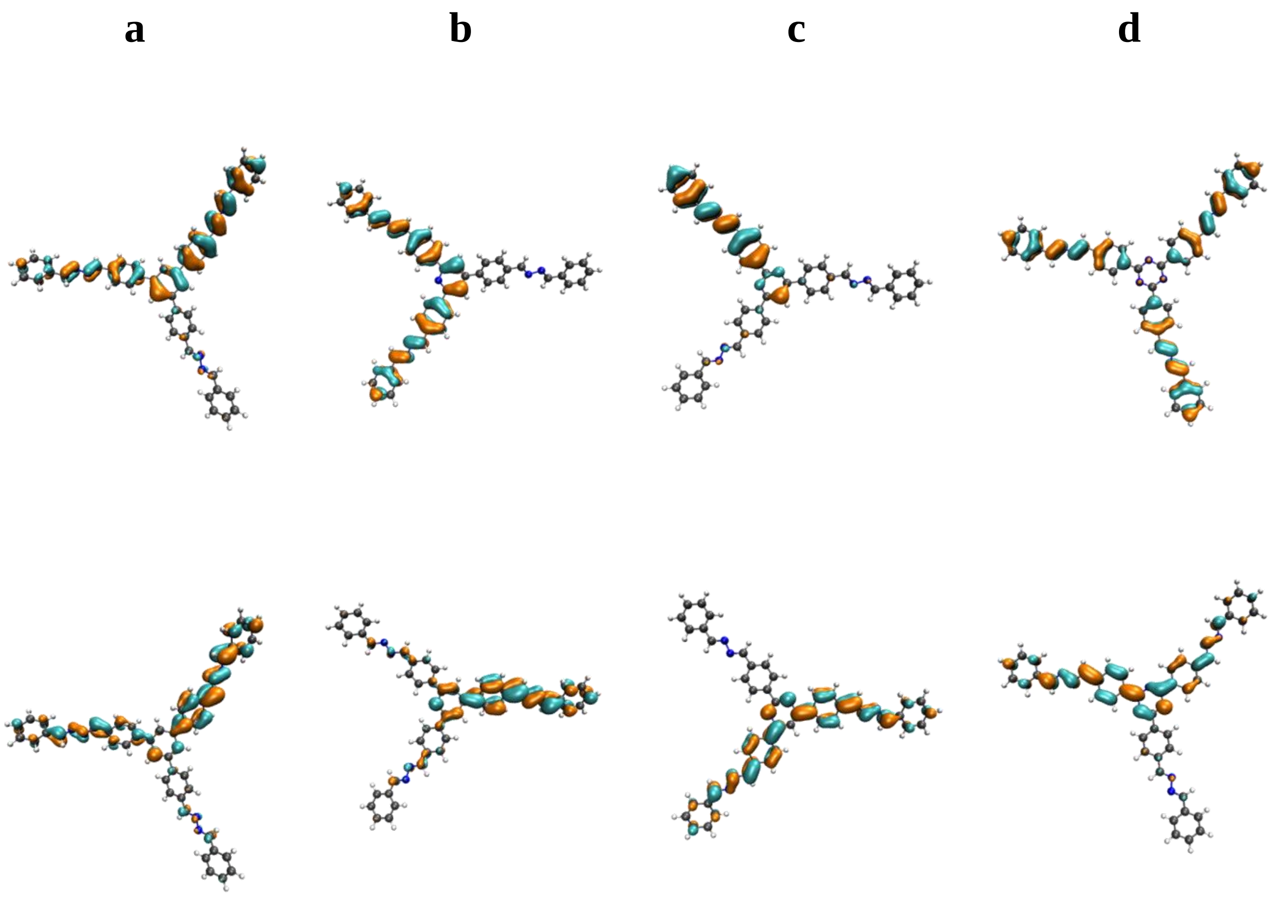

Supplementary Figure 68: HOMO and LUMO obtained at the PBE0-D3/def2-SVP level of theory for N_x-Phenylazines. (a) **N_0-PhAz**, (b) **N_1-PhAz**, (c) **N_2-PhAz**, (d) **N_3-PhAz**.

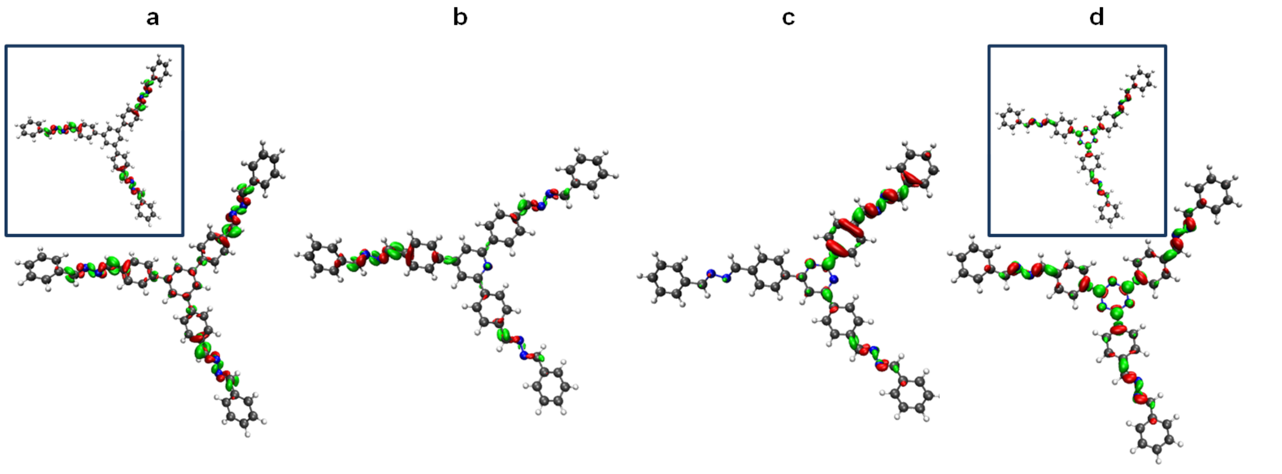

Supplementary Figure 69: Difference densities of vertical excitations with highest oscillator strength obtained at time-dependent (TD)-PBE0/def2-SVP level of theory for N_x-Phenylazines. (a) **N_0-PhAz**, (b) **N_1-PhAz**, (c) **N_2-PhAz**, (d) **N_3-PhAz**. The red isosurfaces depict regions with lower

electron density in the excited state whereas green isosurfaces represent higher electron density in the excited state, both in comparison to the electron density of the ground state. Inset shows the degenerate energy state.

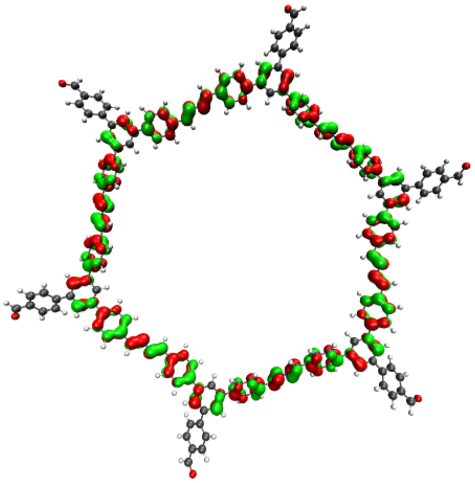

N_0-HxAl

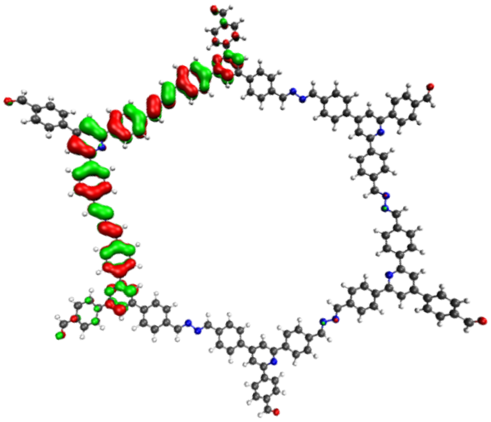

N_1-HxAl

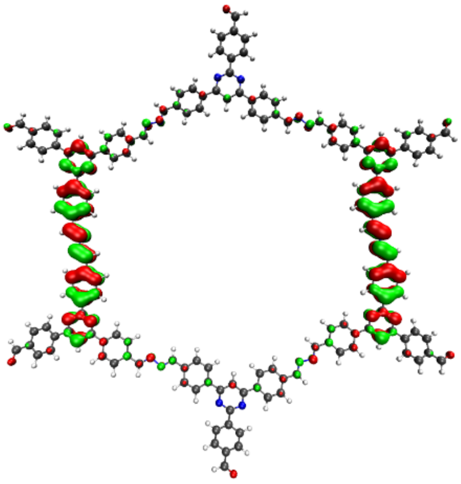

N_2-HxAl

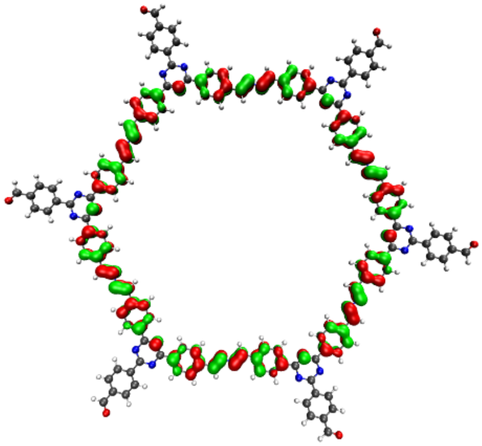

N_3-HxAl

Supplementary Figure 70: Localization of the HOMO for selected model hexagons with aldehyde-(HxAl) terminations, calculated at the PBE0-D3/def2-SVP level of theory.

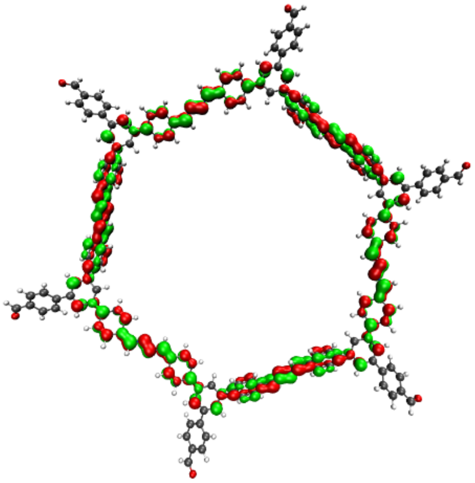

N_0-HxAl

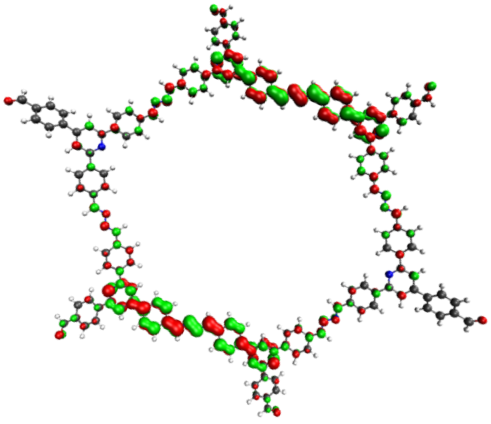

N_1-HxAl

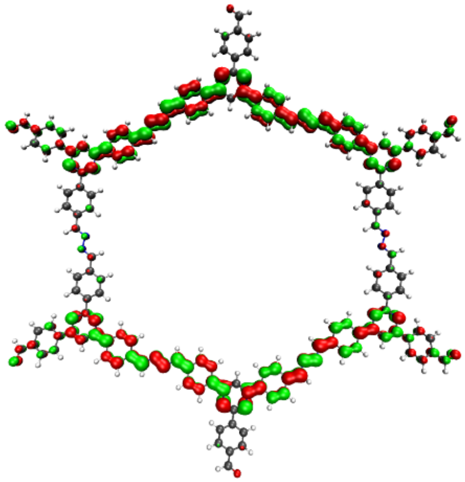

N_2-HxAl

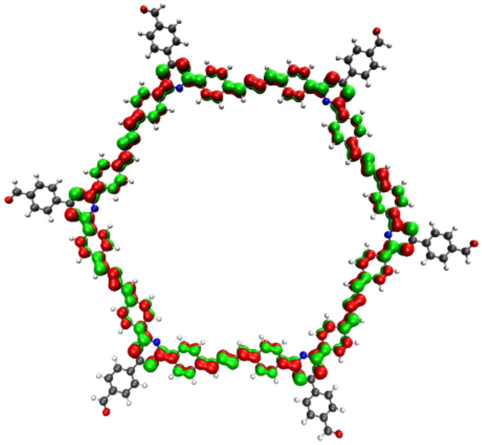

N_3-HxAl

Supplementary Figure 71: Localization of the LUMO for selected model hexagons with aldehyde- (HxAl) terminations, calculated at the PBE0-D3/def2-SVP level of theory.

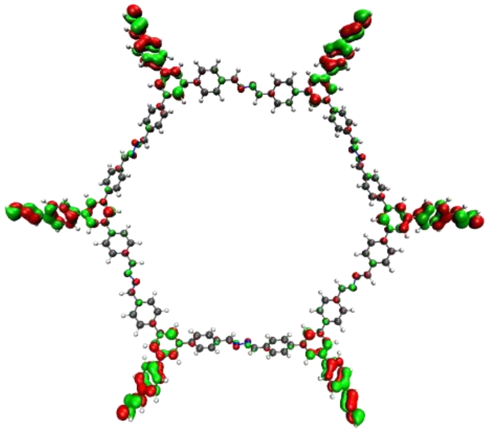

N_0-HxHz

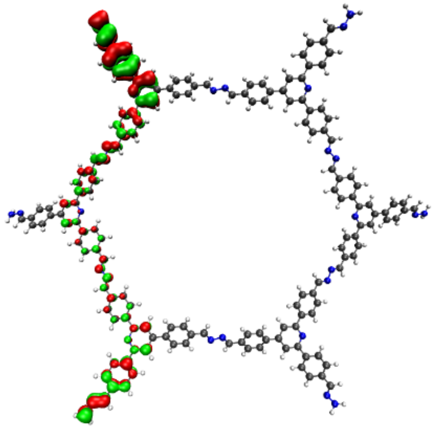

N_1-HxHz

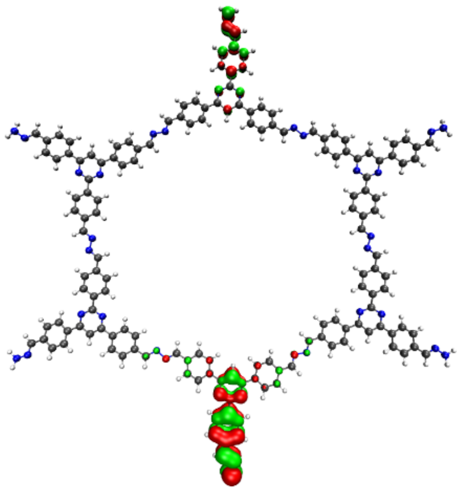

N_2-HxHz

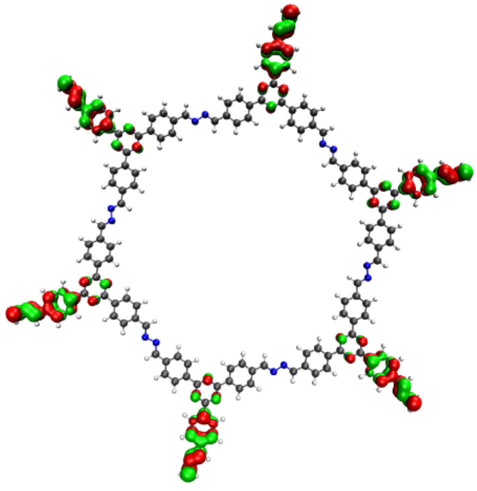

N_3-HxHz

Supplementary Figure 72: Localization of the HOMO for selected model hexagons with hydrazone- (HxHz) terminations, calculated at the PBE0-D3/def2-SVP level of theory.

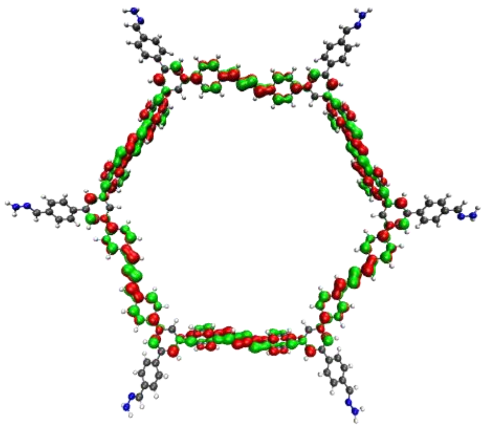

N_0-HxHz

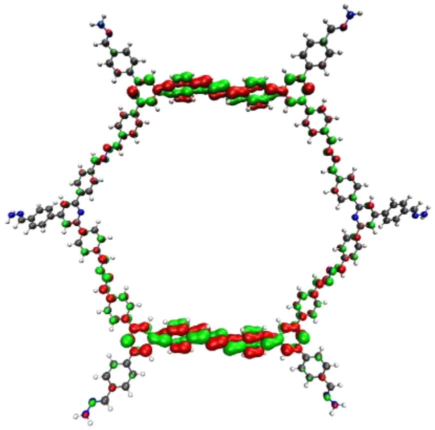

N_1-HxHz

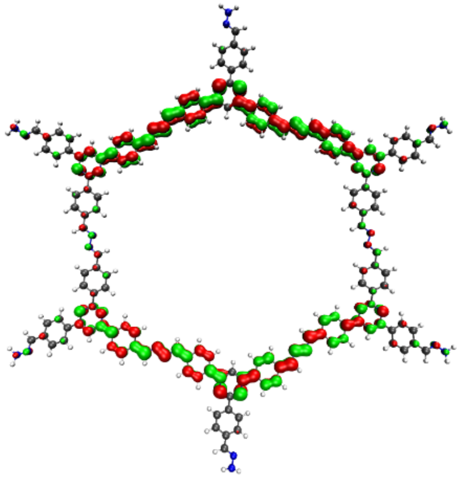

N_2-HxHz

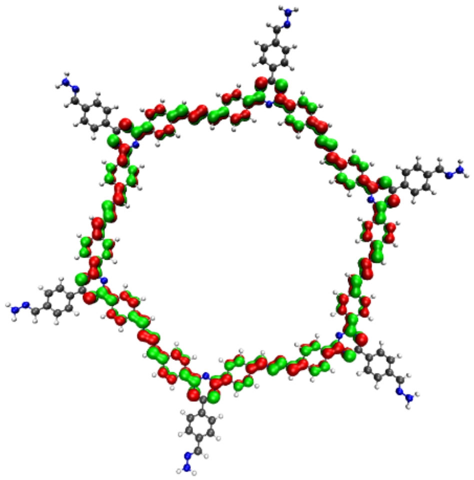

N_3-HxHz

Supplementary Figure 73: Localization of the LUMO for selected model hexagons with hydrazone- (HxHz) terminations, calculated at the PBE0-D3/def2-SVP level of theory.

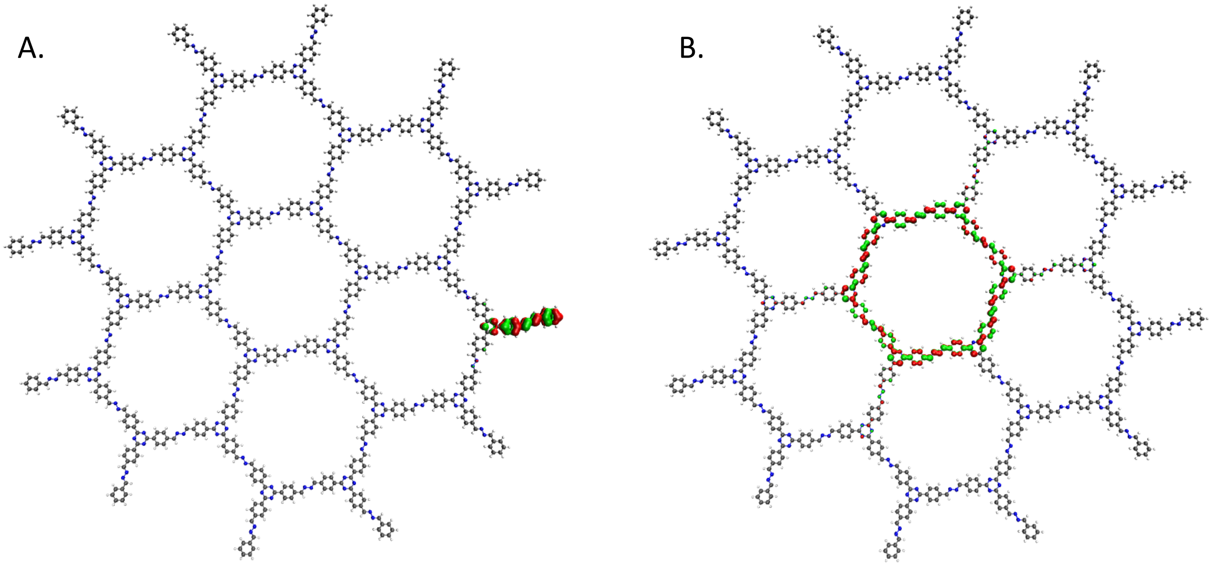

Supplementary Figure 74: Localization of the HOMO (left; A) and LUMO (right; B) for **N_3-7H** model hexagons with Phenylazine terminations, calculated at the PBE0-D3/def2-SVP level of theory.

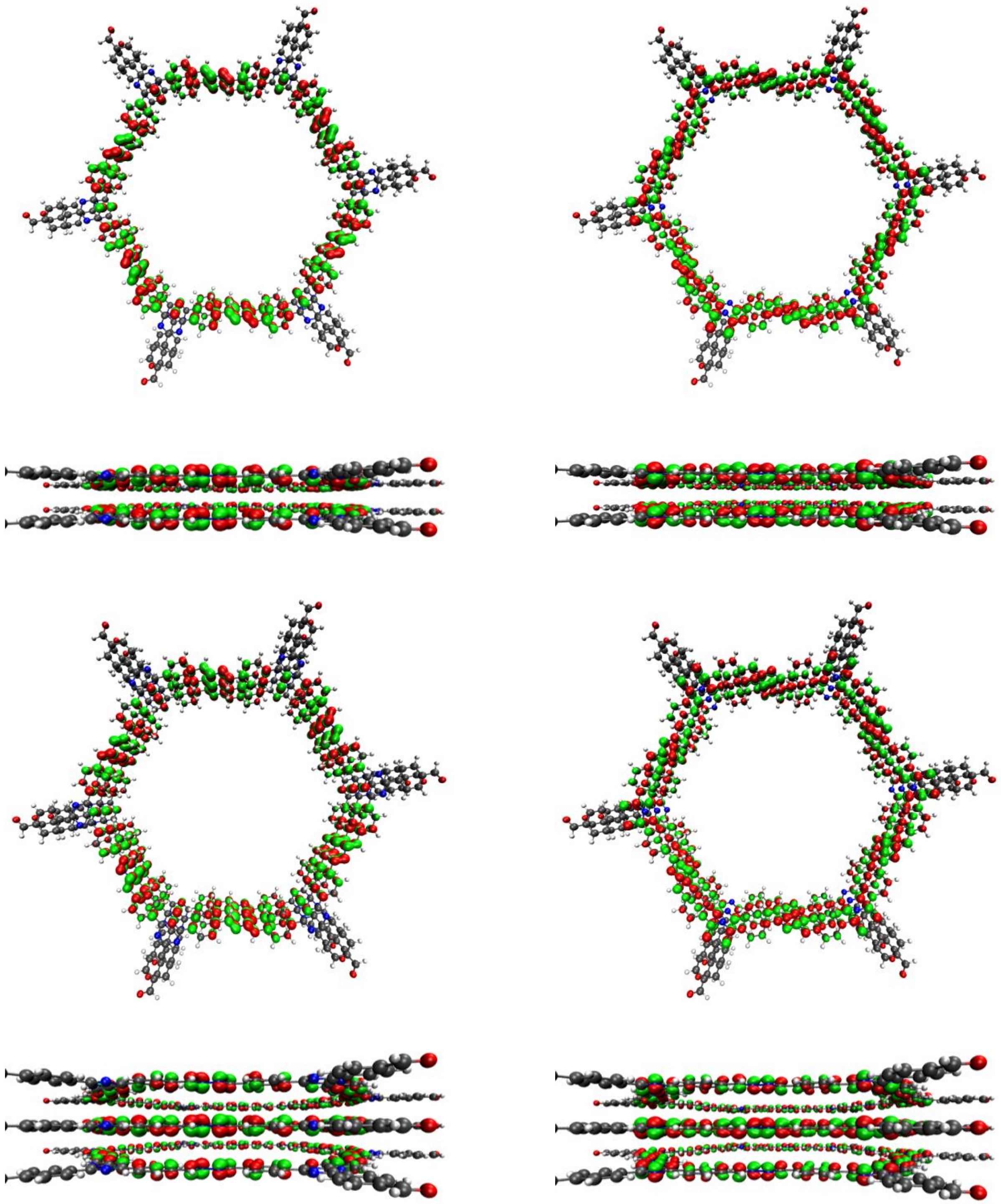

Supplementary Figure 75: Localization of the HOMO (left) and LUMO (right) for two/three layers of the **N_3-HxAl** model hexagons with aldehyde terminations, calculated at the PBE0-D3/def2-SVP level of theory.

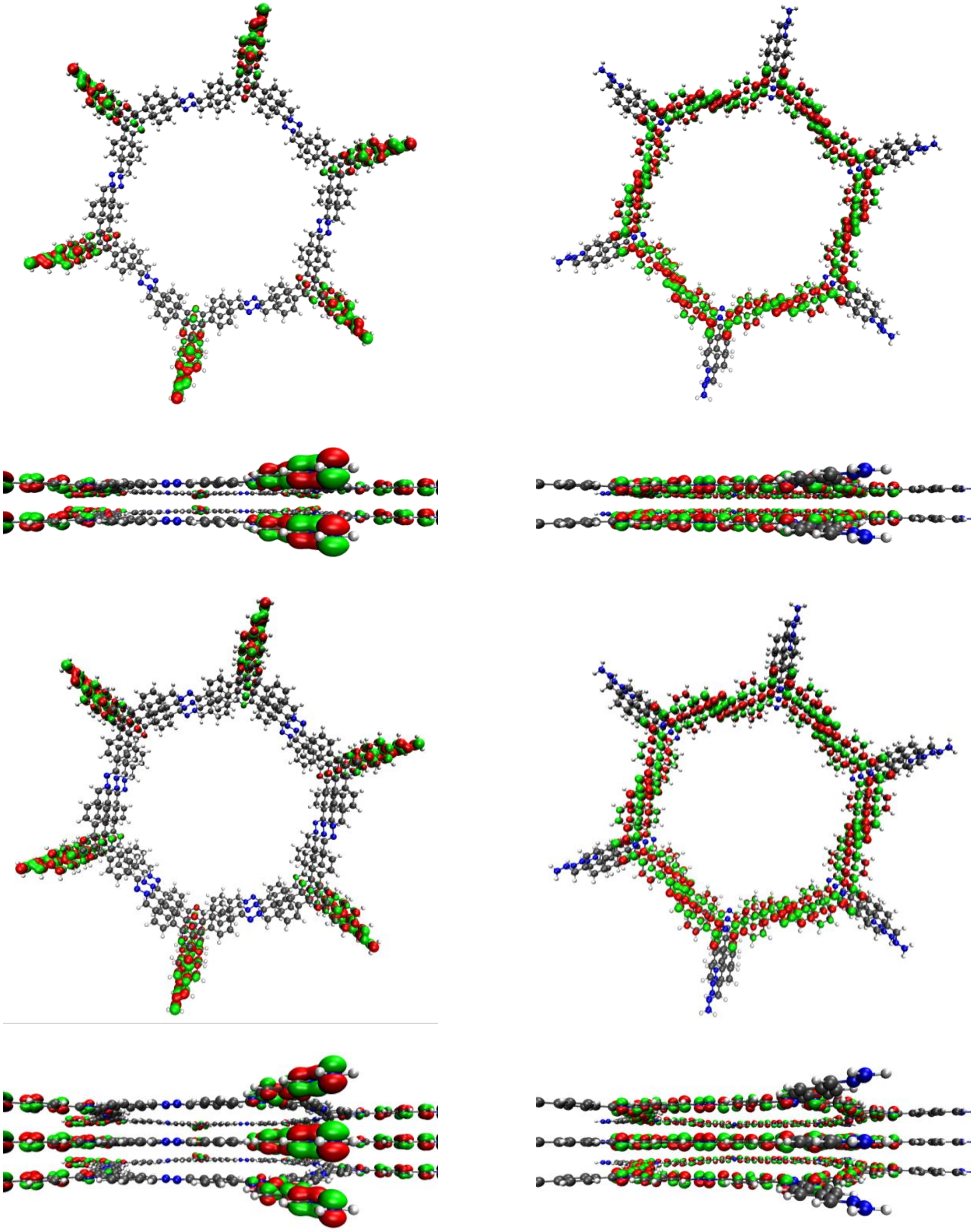

Supplementary Figure 76: Localization of the HOMO (left) and LUMO (right) for two/three layers of the $\mathbf{N_3}$**-HxHz** model hexagons with hydrazone terminations, calculated at the PBE0-D3/def2-SVP level of theory.

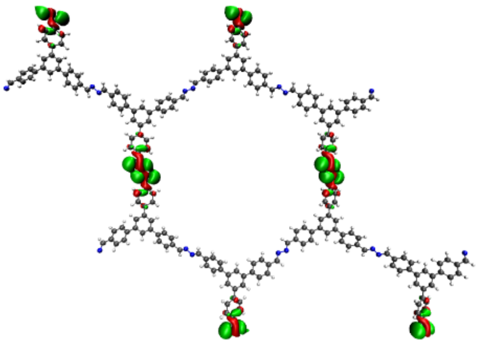

N_0

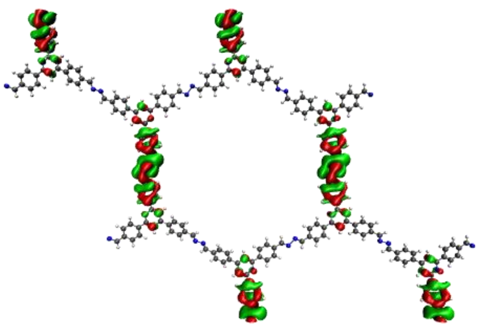

N_1

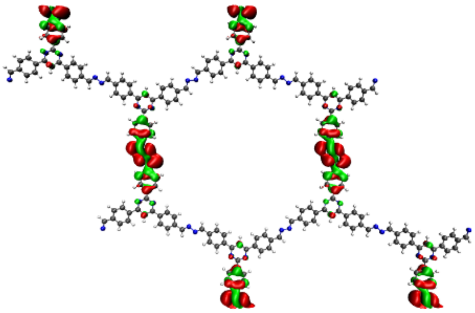

N_2

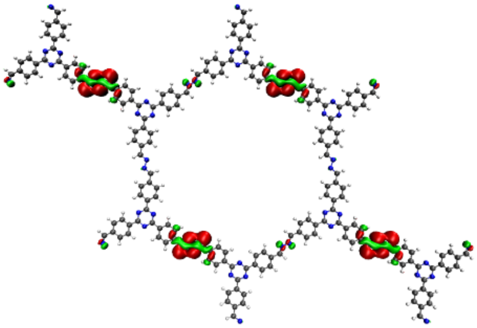

N_3

Supplementary Figure 77: Localization of the HOMO calculated from periodic single points using optimized unit cells at the DFTB+/mio-1-0 level of theory.

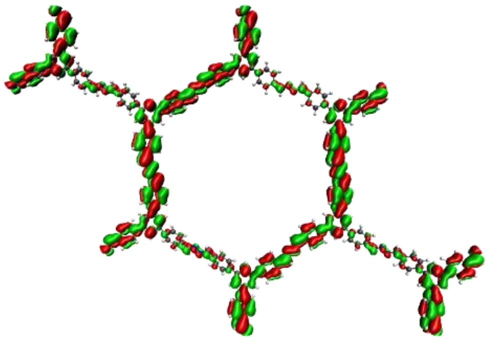

N_0

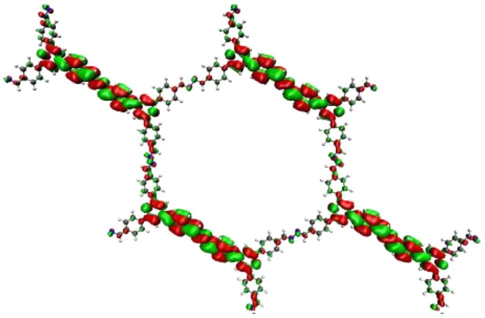

N_1

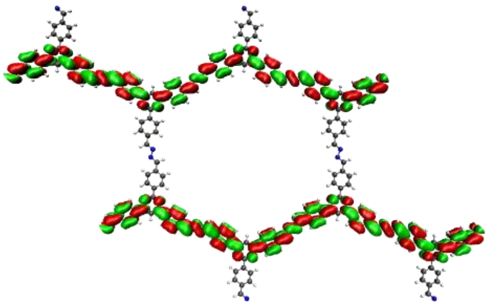

N_2

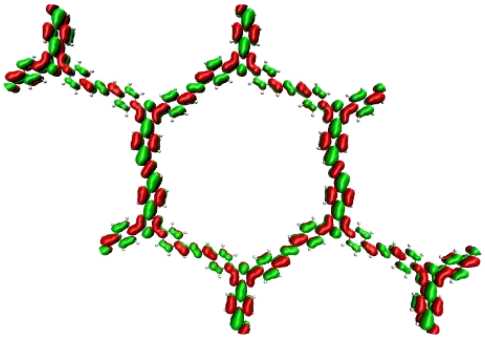

N_3

Supplementary Figure 78: Localization of the LUMO calculated from periodic single points using optimized unit cells at the DFTB+/mio-1-0 level of theory.

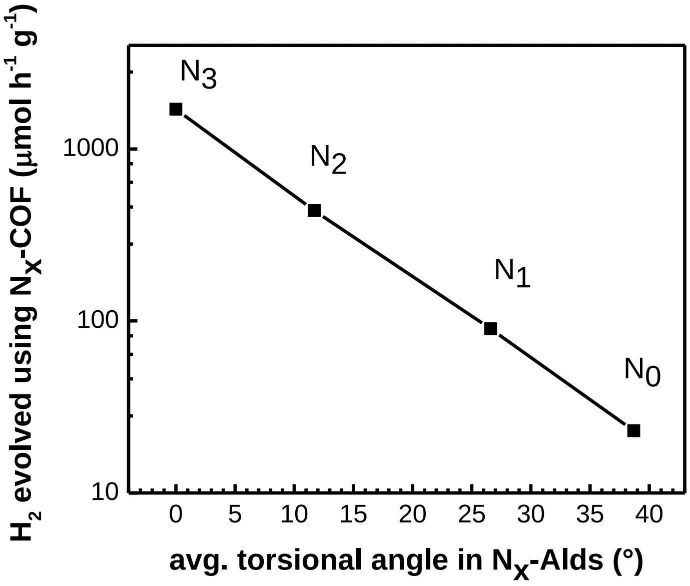

Supplementary Figure 79: Plot showing $\log_{10}$ of hydrogen produced (in µmol h^{-1} g^{-1}) by **N_x-COFs** against average dihedral angles obtained from the geometry optimized structure of their respective precursor aldehydes.

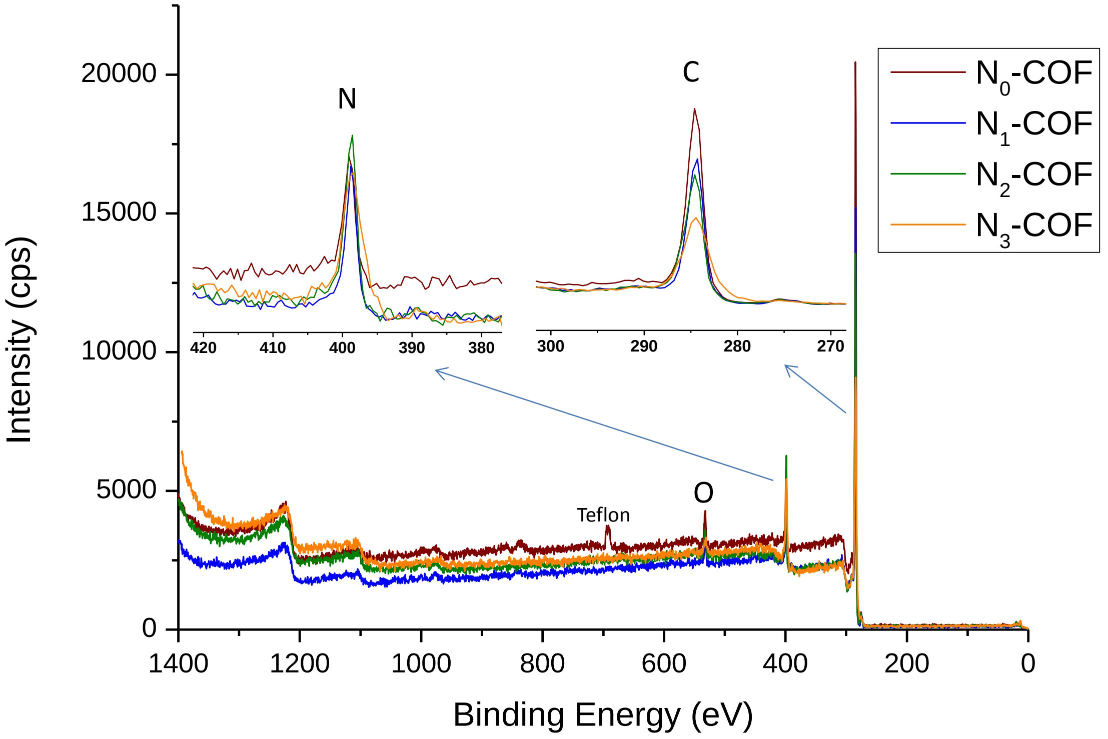

Supplementary Figure 80: XPS spectra of the N_x-COFs. Note that the C 1s peak at the lower binding energy is assigned to the adventitious carbon and is calibrated to 284.8 eV.

2. Supplementary Tables

Supplementary Table 1: Unit cell parameters of **N_x-COFs** obtained from the refinement of the PXRD pattern.

	a	b	c	symmetry
N_0	28.91(1)	28.92(1)	3.4	$P\bar{3}$
N_1	28.45(2)	28.45(2)	3.4	$P\bar{1}$
N_2	29.08(8)	29.08(8)	3.481(6)	$P\bar{1}$
N_3	28.541(5)	28.541(5)	3.438(3)	P6/m

Supplementary Table 2: PE of N_x-COFs using four band pass filters.

COF	Filter CWL (nm)	PE (%)
N_3	400	0.17
	450	0.44
	500	0.26
	550	0.039
N_2	400	0.088
	450	0.19
	500	0.083
	550	0.020
N_1	400	0.038
	450	0.077
	500	0.038
	550	0.0085
N_0	400	0.0016
	450	0.0010
	500	0.0017
	550	0.0003

Supplementary Table 3: Relationship between evolved hydrogen and the BET surface area of **N_x-COFs**.

1	2	3	4
N_x COF	H_2 evolved(Y) μmol h^{-1} g^{-1}	BET area(Z) m^2 g^{-1}	H_2 evolved normalized by surface area (Y/Z ratio)
N_0	23	702	0.0327
N_1	90	326	0.2761
N_2	438	1046	0.4187
N_3	1703	1537	1.1080

Supplementary Table 4: Calculated HOMO and LUMO levels for the $\mathbf{N_x}$-Aldehydes and the Kohn-Sham band gaps.

Compound	HOMO [eV]	LUMO [eV]	Kohn-Sham band gap [eV]
**$\mathbf{N_0}$ -Ald**	-7.16	-2.28	4.89
**$\mathbf{N_1}$ -Ald**	-7.11	-2.58	4.52
**$\mathbf{N_2}$ -Ald**	-7.36	-2.85	4.51
**$\mathbf{N_3}$ -Ald**	-7.71	-2.93	4.78

Supplementary Table 5: Calculated HOMO and LUMO levels for the $\mathbf{N_x}$-Phenylazines and the Kohn-Sham band gaps.

Compound	HOMO [eV]	LUMO [eV]	Kohn-Sham band gap [eV]
**$\mathbf{N_0}$-PhAz**	-6.27	-2.21	4.06
**$\mathbf{N_1}$-PhAz**	-6.20	-2.40	3.80
**$\mathbf{N_2}$-PhAz**	-6.30	-2.57	3.73
**$\mathbf{N_3}$-PhAz**	-6.50	-2.60	3.89

Supplementary Table 6: Calculated HOMO and LUMO levels for **N_x-HxAl** model hexagons and the Kohn-Sham band gaps.

Compound	HOMO [eV]	LUMO [eV]	Kohn-Sham band gap [eV]
**N_0-HxAl**	-5.84	-2.21	3.64
**N_1-HxAl**	-5.82	-2.45	3.37
**N_2-HxAl**	-5.88	-2.62	3.26
**N_3-HxAl**	-5.94	-2.66	3.28

Supplementary Table 7: Calculated HOMO and LUMO levels for **N_x**-HxHz model hexagons and the Kohn-Sham band gaps.

Compound	HOMO [eV]	LUMO [eV]	Kohn-Sham band gap [eV]
**N_0-HxHz**	-6.34	-2.50	3.85
**N_1-HxHz**	-6.32	-2.77	3.55
**N_2-HxHz**	-6.45	-2.98	3.47
**N_3-HxHz**	-6.77	-3.08	3.69

Supplementary Table 8: Calculated HOMO and LUMO levels for stacks of **N_x-HxAl** model hexagons and the Kohn-Sham band gaps.

Compound	HOMO [eV]	LUMO [eV]	Kohn-Sham band gap [eV]
1 Layer **N_3-HxAl**	-6.77	-3.08	3.69
2 Layer **N_3-HxAl**	-6.41	-3.47	2.93
3 Layer **N_3-HxAl**	-6.25	-3.63	2.62

Supplementary Table 9: Calculated HOMO and LUMO levels for stacks of **N_x-HxHz** model hexagons and the Kohn-Sham band gaps.

Compound	HOMO [eV]	LUMO [eV]	Kohn-Sham Band gap [eV]
1 Layer **N_3-HxHz**	-5.94	-2.66	3.28
2 Layer **N_3-HxHz**	-5.45	-2.82	2.63
3 Layer **N_3-HxHz**	-5.19	-2.80	2.39

Supplementary Table 10: Calculated vertical electron affinities as differences in total energies between radical anionic and neutral states of **N_x-HxHz** model systems at the PBE0-D3/def2-SVP level of theory.

Compound	ΔE [eV]	ΔΔE [eV]
**N_0-HxHz**	-1.80	0.00
**N_1-HxHz**	-1.99	-0.19
**N_2-HxHz**	-2.19	-0.39
**N_3-HxHz**	-2.25	-0.45

3. Supplementary Methods

3.1. Precursor Synthesis

Synthesis of 1,3,5-Tris(4-formylphenyl)benzene (**N_0-Ald**)

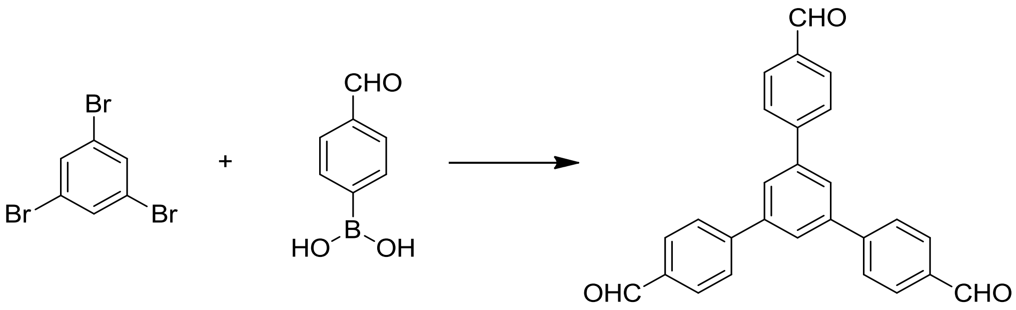

This compound was prepared as per modified literature procedure.[1] In a Schlenk flask equipped with a stir bar and reflux condenser, 1, 3, 5-tribromobenzene (1.0 g, 3.1 mmol), 4-formylphenyl boronic acid (2.2 g, 13.9 mmol), potassium carbonate (2.1 g, 15.0 mmol), and palladiumtetrakis(triphenylphosphine) (0.17 g, 0.15 mmol) were taken. The flask was evacuated and backfilled with argon three times. To this were added toluene (50 mL), ethanol (10 mL) and water (10 mL) and the contents were refluxed for 48 h. The reaction mixture was then cooled to room temperature and water (100 mL) was added. The organic compound was extracted with dichloromethane, dried over anhyd. $MgSO_4$ and then filtered. The solvent was removed under reduced pressure and after column chromatography (silica gel, dichloromethane) pure product was obtained as an off white solid (0.89 g, 73 %). ^{1}H NMR ($CDCl_3$, 300 MHz): δ ppm 10.11 (s, 3H), 8.02 (d, J = 8.3 Hz, 6H), 7.91 (s, 3H), 7.87 (d, J = 8.3 Hz, 6H). ^{13}C NMR ($CDCl_3$, 75 MHz): δ ppm 191.93, 146.49, 141.81, 135.99, 130.64, 128.19, 126.68. MALDI-TOF-MS for $C_{27}H_{18}O_3$: 389.8 M^+ (calculated: 390.1)

Synthesis of 2,4,6-Tris(4-bromophenyl)pyridine (**1**)

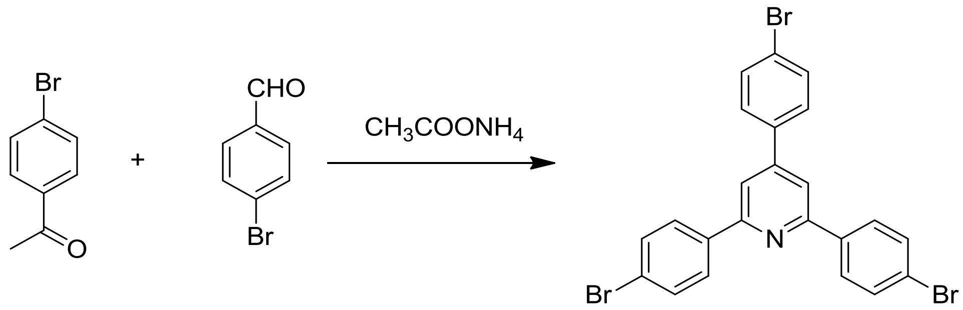

This compound was prepared as per modified literature procedure [2]. In a 20 ml microwave vial equipped with a stir bar was taken a mixture of 4-bromoacetophenone (2.07 g, 10.4 mmol), 4-bromobenzaldehyde (0.97 g, 5.2 mmol), ammonium acetate (14.0 g, 181.6 mmol) and glacial acetic acid (7.3 ml). The contents were flushed with Argon before sealing the vial. The contents were heated in the microwave under autogenous pressure at 220°C for 45 min. The reaction mixture was then cooled to room temperature and water (10 mL) was added. The aqueous suspension was then neutralized by saturated sodium bicarbonate solution. The organic compound was extracted with dichloromethane, dried over anhyd. $MgSO_4$ and then filtered. The solvent was removed under reduced pressure and after recrystallization (acetone), pure product was obtained as off white solid (0.90 g, 31%). ^1H NMR ($CDCl_3$, 300 MHz): δ ppm 8.04 (d, J = 8.4 Hz, 4H), 7.80 (s, 2H), 7.62 (m, 8H). ^{13}C NMR ($CDCl_3$, 75 MHz): δ ppm 156.87, 149.64, 138.25, 137.80, 132.62, 132.16, 128.93, 128.87, 124.05, 123.93, 116.97.

Synthesis of 2,4,6-Tris(4-formylphenyl)pyridine (**N_1-Ald**)

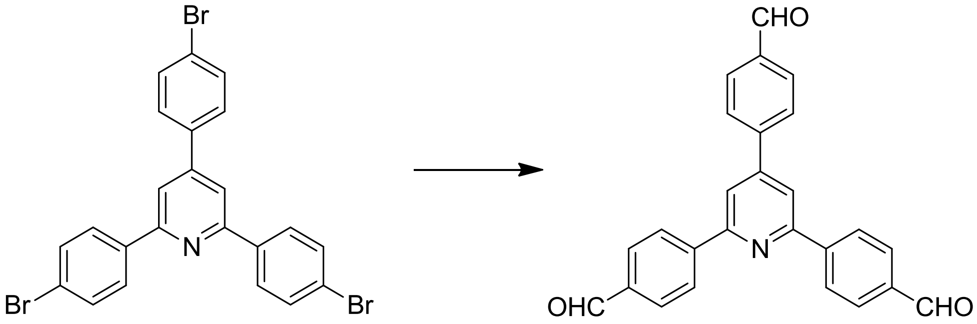

The synthesis was adapted from literature published procedure with modifications [3]. In a Schlenk flask under Argon flow, a suspension of **1** (2,4,6-tris(4-bromophenyl)pyridine) (0.5 g, 0.92 mmol) in dry THF (150 mL) was cooled to -78°C. To this was added a solution of *n*-BuLi (1.1 mL, 2.8 mmol, 2.5 M in hexane). The mixture was stirred for 1 hour and then N-formylpiperidine (0.32 mL, 2.9 mmol) was added. The mixture was stirred for 30 min at -78 °C and then warmed to room temperature. After stirring for an additional 1 h at room temperature, 5 mL of 0.5 M HCl was added. The mixture was extracted with dichloromethane, dried over anhyd. $MgSO_4$ and then filtered. After evaporating the solvent, the product was obtained as an off-white solid (0.34 g, 95 %). ^{1}H NMR ($CDCl_3$, 300 MHz): δ ppm 10.15 (s, 1H), 10.14 (s, 2H), 8.39 (d, J = 8.2 Hz, 4H), 8.05 (m, 8H), 7.92 (d, J = 8.2 Hz, 2H). ^{13}C NMR ($CDCl_3$, 75 MHz): δ ppm 191.77, 191.38, 157.15, 149.96, 144.65, 144.41, 137.41, 137.34, 130.75, 130.41, 128.23, 128.04, 118.89. MALDI-TOF-MS for $C_{26}H_{17}NO_3$: 391.0 M^+ (calculated: 391.1)

Synthesis of 2,4,6-Tris(4-formylphenyl)pyrimidine (**N_2-Ald**)

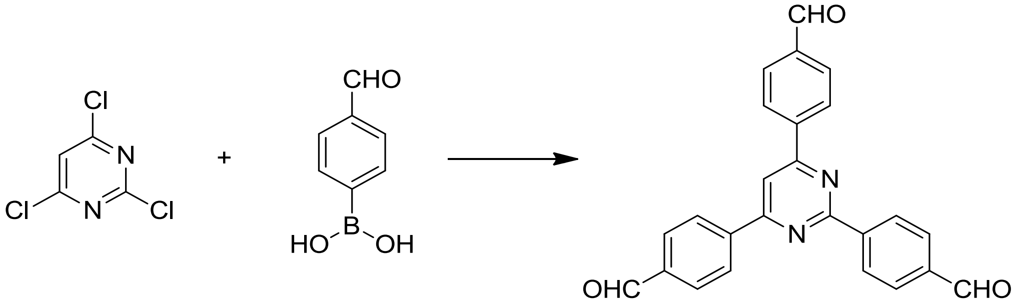

This was prepared as per modified literature procedure [4]. In a Schlenk flask equipped with a stir bar and reflux condenser, 2, 4, 6-trichloropyrimidine (0.15 g, 0.8 mmol), 4-formylphenylboronicacid (0.56 g, 3.6 mmol), potassium carbonate (0.3 g, 2.4 mmol), cesium carbonate (0.78 g, 2.4 mmol),and palladiumtetrakis(triphenylphosphine) (0.14 g, 0.12 mmol) were taken. The flask was evacuated and backfilled with argon three times. To this were added toluene (25 mL), ethanol (5 mL) and water (5 mL) and the contents were refluxed for 72 h. The reaction mixture was then cooled to room temperature and water (100 mL) was added. The organic compound was extracted with dichloromethane, dried over anhyd. $MgSO_4$ and then filtered. The solvent was removed under reduced pressure and after column chromatography [silica gel, ethylacetate:hexanes (1:3) followed by methanol] pure product was obtained that was further recrystallized from acetone to yield an off white solid (0.15 g, 48 %). ^{1}H NMR ($CDCl_3$, 300 MHz): δ ppm 10.17 (s, 3H), 8.90 (d, J = 8.2 Hz, 2H), 8.48 (d, J = 8.2 Hz, 4H), 8.12 (m, 7H). ^{13}C NMR ($CDCl_3$, 75 MHz): δ ppm 192.24, 191.80, 164.47, 164.16, 143.05, 142.44, 138.36, 138.30, 130.52, 130.16, 129.36, 128.26, 112.42. MALDI-TOF-MS for $C_{25}H_{16}N_2O_3$: 392.0 M^+ (calculated: 392.1).

Synthesis of 2,4,6-Tris(4-bromophenyl)-1,3,5-triazine (**2**)

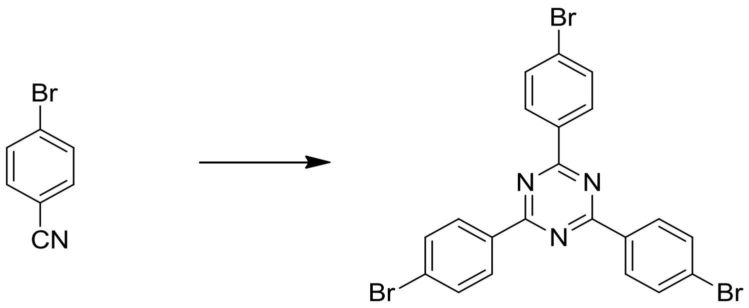

This compound was prepared as per modified literature procedure[5]. In a 15 ml Schlenk tube under Argon, was taken triflic acid (4.0 ml, 6.7 g, 44.6 mmol) and cooled to 0 °C. To this was added 4-bromobenzonitrile (1.5 g, 8.2 mmol) in portions and the solution was continued to stir for an hour at 0 °C and then 16 hours at room temperature. The workup was done by pouring the reaction mixture in 100 mL ice cold water and neutralizing the resulting suspension with 25 % ammonia solution. The precipitate was filtered off, washed with water (2 x 10 mL) acetone (3 x 5 mL) and dried in vacuum to afford the title compound as off white solid (1.4 g, 94 %). ^{1}H NMR ($CDCl_3$, 300 MHz): δ ppm 8.59 (d, *J* = 8.4 Hz, 6H), 7.72 (d, *J* = 8.4 Hz, 6H). ^{13}C NMR ($CDCl_3$, 75 MHz): *δ* ppm 171.37, 135.07, 132.10, 130.55, 127.88.

Synthesis of 2,4,6-Tris(4-formylphenyl)-1,3,5-triazine (**N_3-Ald**)

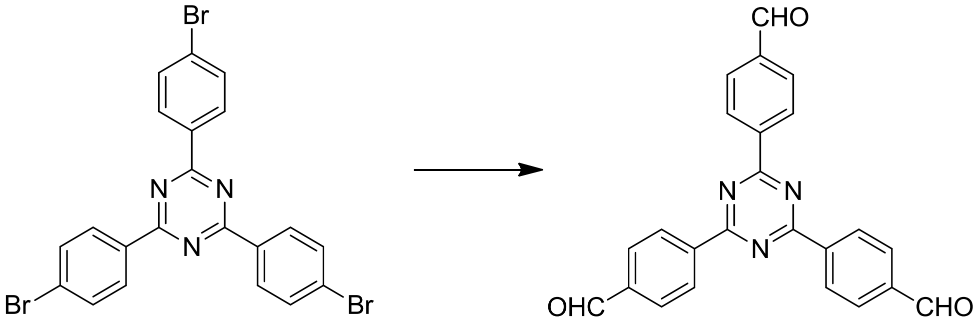

The synthesis was adapted from literature published procedure with modifications [3]. In a Schlenk flask under Argon flow, a suspension of 2,4,6-tris(4-bromophenyl)-1,3,5-triazine (**2**) (0.5 g, 0.9 mmol) in THF (150 mL) was cooled to -78 °C. To this was added a solution of *n*-BuLi (1.1 mL, 2.7 mmol, 2.5 M in hexane). The mixture was stirred for 1 hour and then N-formylpiperidine (0.32 mL, 2.9 mmol) was added. The mixture was stirred for 30 min at -78 °C and then warmed to room temperature. After stirring for an additional 1 h at room temperature, 5 mL of 0.5 M HCl was added. The mixture was extracted with dichloromethane, dried over anhyd. $MgSO_4$ and then filtered. After evaporating the solvent, the residue was obtained as an off-white solid (0.29 g, 81 %). ^{1}H NMR ($C_2D_2Cl_4$, 70 °C, 300 MHz): δ ppm 10.20 (s, 3H), 8.94 (d, J = 8.2 Hz, 6H), 8.13 (d, J = 8.2 Hz, 6H). ^{13}C NMR ($C_2D_2Cl_4$, 70 °C, 75 MHz): δ ppm 191.36, 171.16, 140.57, 139.25, 129.60, 129.45. MALDI-TOF-MS for $C_{24}H_{15}N_3O_3$: 393.0 M^+ (calculated: 393.1).

3.2. General Synthesis of COFs

All COFs were prepared by a procedure identical to the one described for the synthesis of **N_3-COF**.

Synthesis of **N_3-COF**

In a Biotage® 5 mL high precision glass vial, was suspended **N_3-Ald** (50 mg, 0.13 mmol) in a mixture 1.0 mL of mesitylene, 1.0 mL of 1,4-dioxane and 100 μL aqueous 6M acetic acid. To the

suspension was then added hydrazine hydrate (10 μL, 50-60 % solution, Sigma Aldrich). The vial was then sealed and heated in an oil bath at 120 °C for 3 days at autogenous pressure. Thereafter, the vial was opened and the suspension was filtered and washed with chloroform (2 x 5 mL), acetone (2 x 5 mL) and THF (2 x 5 mL). The solid was dried in an oven at 60 °C to afford **N_3-COF** as light yellow powder. Anal. Calcd. for **N_3-COF** $(C_{24}N_6H_{15})_n$: C, 74.40; N, 21.69; H, 3.90. Found: C, 72.36; N, 21.32; H, 4.07. Anal. Calcd. for N_2-COF $(C_{25}H_{16}N_5)_n$: C, 77.70; N, 18.12; H, 4.17. Found: C, 71.85; N, 16.21; H, 4.40. Anal. Calcd. for N_1-COF $(C_{26}H_{17}N_4)_n$: C, 81.02; N, 14.54; H, 4.45. Found: C, 72.48; N, 12.10; H, 4.55. Anal. Calcd. for **N_0-COF** $(C_{27}H_{18}N_3)_n$: C, 84.35; N, 10.93; H, 4.72. Found: C, 80.15; N, 9.99; H, 4.76.

3.3. Structure Simulation and Powder X-Ray Diffraction of COFs

Molecular modeling of the COF was carried out using the Materials Studio (v6.0.0) suite of programs by Accelrys.[6] The structure was geometry optimized using the MS Forcite molecular dynamics module (Universal force fields, Ewald summations), and the unit cell parameters were received from Pawley refinement of the powder pattern. The COFs were modeled with imposed symmetry that is conceived from the native connectivity and geometry of the building blocks. The length of the *c* axis in the refinement of **N_0** and **N_1** was fixed at 3.4 Å, since no stacking peak was observed for these materials that allowed refinement of this value. The **N_1-COF** and **N_2-COF** were simulated with the symmetry $P\overline{1}$, which is the highest symmetry in these COFs if the aryl nitrogens are ordered. Note that assuming disorder for the orientation of the pyridine and pyrimidine rings, a higher symmetry such as $P\overline{3}$ is conceivable.

The obtained unit cell parameters and symmetry constraints are summarized in table 1.

4. Supplementary References

1. Wang, Z.M., et al., *Phenanthro[9,10-d]imidazole as a new building block for blue light emitting materials.* Journal of Materials Chemistry, 2011. **21**(14): p. 5451-5456.

2. Mikroyannidis, J.A., S.H. Ye, and Y.Q. Liu, *Electroluminesent divinylene- and trivinylene-molecules with terminal naphthalimide or phthalimide segments.* Synthetic Metals, 2009. **159**(5-6): p. 492-500.

3. Hartman, G.D., W. Halczenko, and B.T. Phillips, *Iminium Ion Mediated Cyclizations of 4-Aryl-1,4-Dihydropyridines - Bridging with Olefins.* Journal of Organic Chemistry, 1985. **50**(14): p. 2427-2431.

4. Achelle, S., et al., *Star- and banana-shaped oligomers with a pyrimidine core: Synthesis and light-emitting properties.* European Journal of Organic Chemistry, 2008(18): p. 3129-3140.

5. Tanaka, H., et al., *Twisted Intramolecular Charge Transfer State for Long-Wavelength Thermally Activated Delayed Fluorescence.* Chemistry of Materials, 2013. **25**(18): p. 3766-3771.

6. *Materials Studio v6.0.0.*
